# Codon Pairs are Phylogenetically Conserved: A comprehensive analysis of codon pairing conservation across the Tree of Life

**DOI:** 10.1371/journal.pone.0232260

**Published:** 2020-05-13

**Authors:** Justin B. Miller, Lauren M. McKinnon, Michael F. Whiting, John S. K. Kauwe, Perry G. Ridge

**Affiliations:** 1 Department of Biology, Brigham Young University, Provo, UT, United States of America; 2 M.L. Bean Museum, Brigham Young University, Provo, UT, United States of America; Universite de Lausanne Faculte de biologie et medecine, SWITZERLAND

## Abstract

Identical codon pairing and co-tRNA codon pairing increase translational efficiency within genes when two codons that encode the same amino acid are translated by the same tRNA before it diffuses from the ribosome. We examine the phylogenetic signal in both identical and co-tRNA codon pairing across 23 428 species using alignment-free and parsimony methods. We determined that conserved codon pairing typically has a smaller window size than the length of a ribosome, and codon pairing tracks phylogenies across various taxonomic groups. We report a comprehensive analysis of codon pairing, including the extent to which each codon pairs. Our parsimony method generally recovers phylogenies that are more congruent with the established phylogenies than our alignment-free method. However, four of the ten taxonomic groups did not have sufficient orthologous codon pairings and were therefore analyzed using only the alignment-free methods. Since the recovered phylogenies using only codon pairing largely match phylogenies from the Open Tree of Life and the NCBI taxonomy, and are comparable to trees recovered by other algorithms, we propose that codon pairing biases are phylogenetically conserved and should be considered in conjunction with other phylogenomic techniques.

## Introduction

Phylogenies allow biologists to infer similar characteristics of closely related species and provide an evolutionary framework for analyzing biological patterns [1]. Phylogenies are statements of homology, and represent a continuity of biological information [2]. Although genetic data facilitate the analysis of diverse species, molecular data typically require data cleaning (e.g., alignment, annotation, and ortholog identification) before they become useful [3]. Furthermore, contaminations and deep unrecognized paralogy often cause single-gene trees and species trees to be incongruent [3]. However, when these issues are properly handled and orthologs are identified, phylogenies can be recovered through parsimony [4, 5], maximum likelihood [6], Bayesian inference [7], or distance-based techniques such as neighbor-joining [8].

Alignment-free methods have recently gained traction because they do not require a sequence alignment, which allows species with unannotated genes to be placed on the species tree. Furthermore, the alignment-free algorithms are usually computationally inexpensive, which allows for more species to be compared together. Proponents of alignment-free techniques claim that they are resistant to shuffling and recombination events and are not affected by assumptions regarding a high correlation between sequence changes and evolutionary time [9]. Alignment-free techniques typically use Chaos Theory to calculate distances of basic genomic features (e.g., GC content, oligomer frequency, etc.) that are then used to recover the phylogeny [10, 11]. Since genomic features are compared between species instead of sequence homology, these genomic features can be located on different chromosomes or encompass the whole genome. More recently, another technique limits the alignment-free search space to all genic regions within a species, comparing species-wide patterns of codon aversion and amino acid aversion independent of gene annotations [12]. Generally, alignment-free approaches can be grouped into three main types. The first group determines the frequency of words of a certain length (e.g., FFP [13, 14] and CVTree [15]). The second group finds match lengths between sequences (e.g., ACS [16], KMACS [17], and Kr [18]). The last group calculates informational content between sequences (e.g., Co-phylog [19], FSWM [20], andi [21], and CAM [12]).

Although sequence alignments are typically used in parsimony, other features, such as the aversion to certain codons, can also be used to recover phylogenies [22, 23]. In those analyses, each ortholog was encoded with 64 characters, one for each codon. Codons were given a binary representation of '1' if the codon was used within the ortholog and '0' if the codon was not used, each character was added to a matrix, and phylogenies were recovered using parsimony and alignment-free methods.

Codons are sequences of three consecutive nucleotides of coding DNA that are transcribed into mRNA, mRNA is translated into amino acids, and amino acids form proteins [24]. The 20 canonical amino acids are formed from 61 codons, with the other three codons encoding the stop signal [25]. Although multiple codons encode the same amino acid, both mutation and selection can cause an unequal distribution of synonymous codons to occur within species [26], suggesting that synonymous codons might play different roles in species fitness [27]. An unequal distribution of tRNA anticodons directly coupling codons led to the wobble hypothesis: tRNA anticodons do not need to bind to all three codon nucleotides for translation [28]. Codon usage is also highly associated with the most abundant tRNA present in the cell [29] and codon usage patterns affect gene expression [30].

Recharging a tRNA while the tRNA is still attached to the ribosome increases translational efficiency and decreases overall resource utilization. This process occurs when codons encoding the same amino acid are located in close proximity to each other on the mRNA strand [31]. Co-tRNA codon pairing is when two non-identical codons that encode the same amino acid are near each other in a gene and the tRNA is recharged to translate both codons before the tRNA diffuses. Similarly, identical codon pairing occurs when identical codons are near each other in a gene and the tRNA is recharged to translate both codons before the tRNA diffuses. Co-tRNA and identical codon pairing conserve resources and increase translational efficiency by approximately 30% [31]. Co-tRNA codon pairing has previously been reported as more prominent in eukaryotes, while identical codon pairing has been reported in eukaryotes, bacteria [32], and archaea [33].

Here, we present two novel approaches that capitalize on biases in codon pairing to determine species relationships using either a parsimony or alignment-free method. We perform a comprehensive analysis of codon pairing across the Tree of Life and explain how codon pairing can be implemented in a phylogenomic framework.

## Results

Table 1 report the number of species that were included in each analysis after the preprocessing filters were applied (e.g., each species in the parsimony analysis included at least 5% of the parsimony-informative characters). In total, we included 23 428 species, with each species generally containing thousands of genes. S1‒S3 Tables in S1 File report the number of species that were included for each ribosomal window size in each of the three parsimony analyses: identical codon pairing, co-tRNA codon pairing, and a combined approach. The alignment-free methods analyzed all species because the methods are not affected by missing ortholog calls.

**Table 1:** Number of species passing preprocessing filters and analyzed by each algorithm.

Taxonomic Group	Alignment-free	Parsimony	Maximum Likelihood	NCBI Taxonomy	OTL
All	23 428	0	0	22 794	12 337
Archaea	418	100	418	416	362
Bacteria*	15 068	0	0	14 612	11 227
Fungi	234	0	58	234	214
Invertebrates	149	57	57	149	147
Plants	89	61	60	89	87
Protozoa	75	15	24	75	75
Mammals	107	97	100	107	105
Other vertebrates	123	114	118	123	120
Viruses*	7 233	0	0	7 045	0

The alignment-free methods did not require any preprocessing of the coding sequences. Parsimony used a stricter preprocessing cutoff than maximum likelihood, and therefore used fewer species. The NCBI taxonomy includes viruses and more species than the OTL. We did not run ortholog-based phylogenetic methods on taxonomic groups when fewer than 5% of the total species remained after initial filtering.

*Sixty-eight bacteria and viruses overlap.

We used reference phylogenies from the National Center for Biotechnology Information (NCBI) Taxonomy Browser [34–37] and the Open Tree of Life (OTL) [38]. The NCBI taxonomy contains more species than the OTL, and the OTL does not contain any viruses. The species trees vary between the OTL and the NCBI taxonomy by 1–9%, with the mammal phylogenies being the most similar and the fungi phylogenies being the least similar. Descriptions on filters applied to parsimony and maximum likelihood are found in S1 Text in S1 File.

After filtering for parsimony-informative codons, we used parsimony to recover phylogenies with the highest percent overlap based on binary representations of codon pairings (i.e., if the codon occurred in a gene, it was encoded as '1' and if it did not occur, it was encoded as '0'). We opted to use the branch congruence (i.e., percentage of edge similarity) metric to compare our recovered phylogenies because it is less sensitive to polytomies, small changes in leaf nodes, unrooted trees, and large phylogenies such as the trees recovered in this study. Branch congruence was also used by Miller, McKinnon [12], which facilitates the direct comparison of our results with the results from their study. The number of parsimony-informative codon pairings used in each comparison are reported in S4‒S6 Tables in S1 File, with mammals typically having the most parsimony-informative characters and invertebrates typically having the fewest parsimony-informative characters.

**Fig 1:**
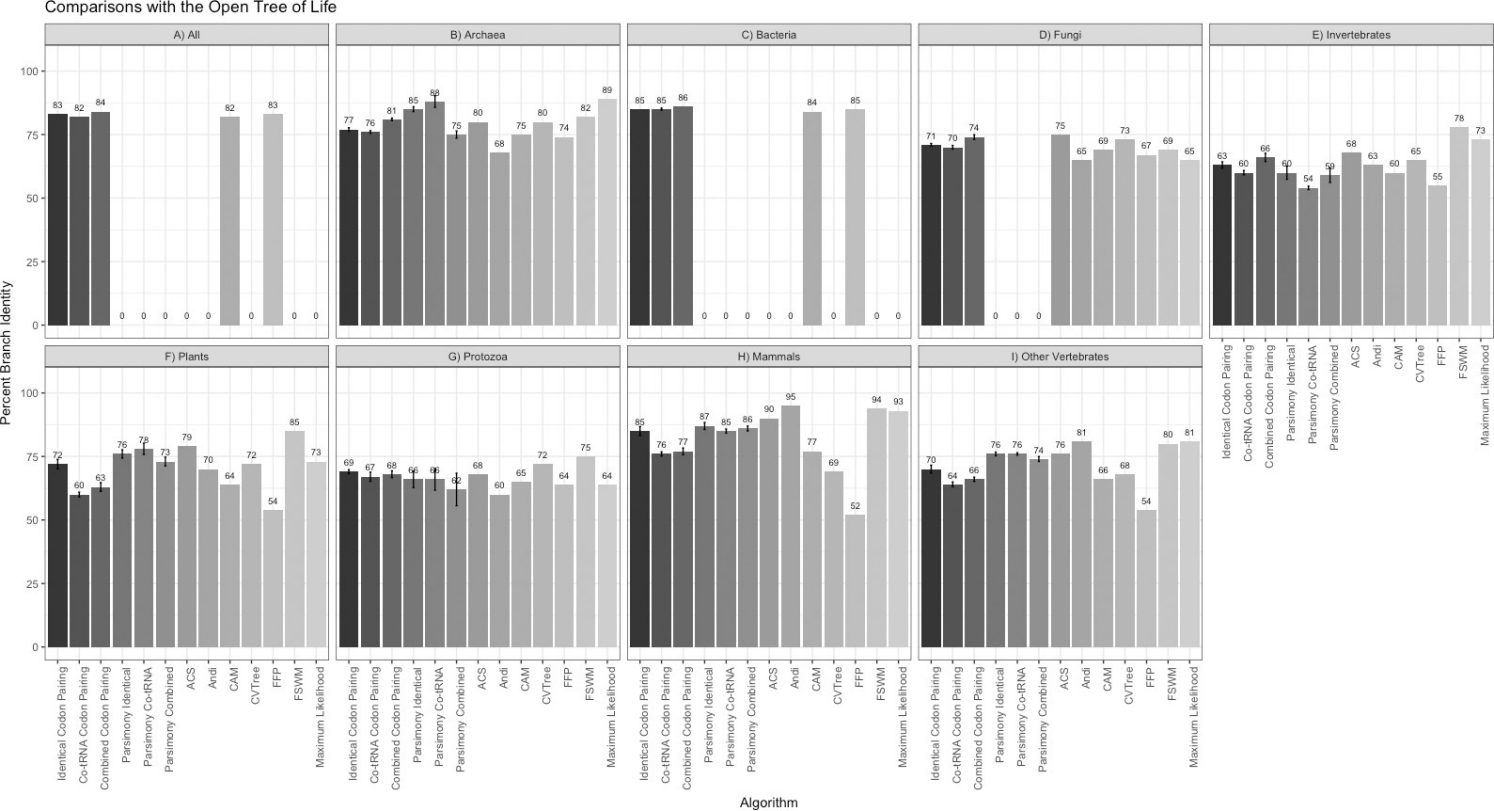
Percent edge overlap for comparisons of each algorithm against the OTL. The alignment-free and parsimony codon pairing methods report the mean percent edge overlap with the OTL based on using different ribosome windows from 2–11. Error bars are reported for the codon pairing methods, signifying one standard deviation from the mean. The other methods were previously reported in Miller, McKinnon [12] and are used for comparison.

**Fig 2:**
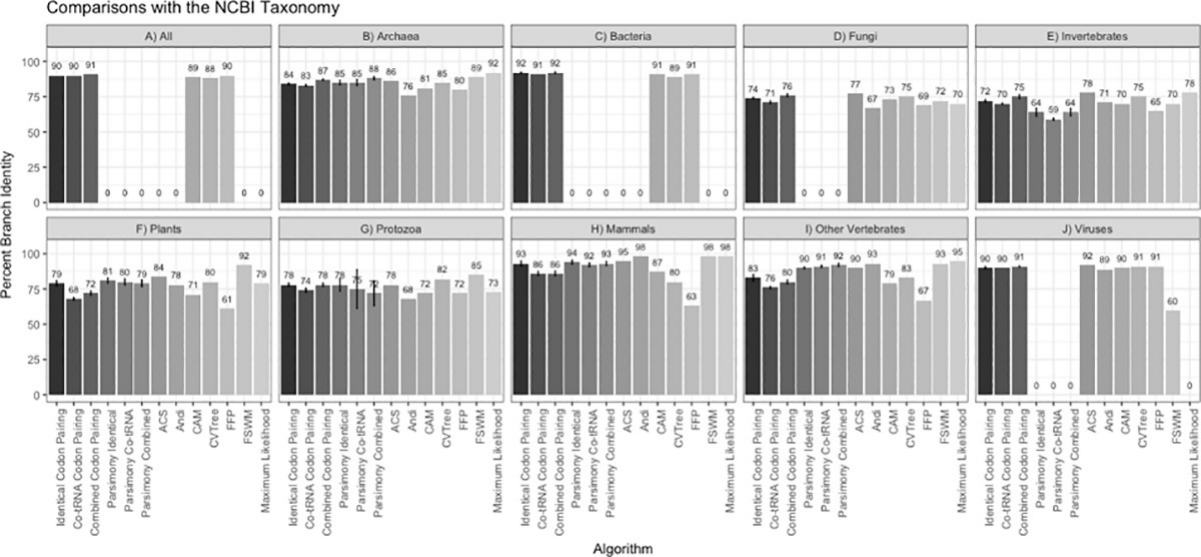
Percent edge overlap for comparisons of each algorithm against the NCBI taxonomy. The alignment-free and parsimony codon pairing methods report the mean percent edge overlap with the NCBI taxonomy based on using different ribosome windows from 2–11. Error bars are reported for the codon pairing methods, signifying one standard deviation from the mean. The other methods were previously reported in Miller, McKinnon [12] and are used for comparison.

Fig 1 shows the percent overlap of the unrooted trees recovered using the six codon pairing methods (identical codon pairing, co-tRNA codon pairing, and combined codon pairing for parsimony and alignment-free) compared to the OTL. For comparison, trees recovered from other alignment-free methods (CAM, FFP, CVTree, ACS, Andi, and FSWM) and maximum likelihood are also compared to the OTL in Fig 1. Fig 2 shows unrooted tree comparisons for each method compared to the NCBI taxonomy.

The alignment-free and parsimony codon pairing methods recovered phylogenetic relationships that are highly congruent with both the OTL and the NCBI taxonomy. The alignment-free method had a branch percent identity ranging from 62% to 86% with the OTL taxonomy, and a range of 68.4% to 92.6% for the NCBI taxonomy. The parsimony pairing method performed slightly better with branch percent identities ranging from 64% to 90% with the OTL, and 63.5% to 94% with the NCBI taxonomy. The alignment-free method outperformed parsimony when compared to the OTL only for invertebrates, which also had the fewest parsimony-informative characters and is paraphyletic.

The comparisons of the codon pairing algorithms to maximum likelihood and other alignment-free algorithms show that none of the phylogenetic algorithms consistently recovered phylogenies with the highest percent edge similarity with the OTL or the NCBI taxonomy. However, the codon pairing algorithms consistently outperformed CAM and FFP. The codon pairing methods were comparable to maximum likelihood, CVTree, ACS, Andi, and FSWM in all taxonomic groups.

S7 Table in S1 File shows the optimal window sizes and the method (identical, co-tRNA, or combined codon pairing) that recovered the most congruent tree with the established phylogenies, with a description of the results in S2 Text in S1 File. S8-S19 Tables in S1 File report the percent edge overlap for identical, co-tRNA, and combined codon pairing compared to the OTL and the NCBI taxonomy for both the alignment-free and parsimony approaches at each ribosome window size from 2–11 codons. For both the alignment-free and parsimony approaches, combining co-tRNA codon pairing with identical codon pairing produced the most congruent tree with the OTL and the NCBI Taxonomy in most taxonomic groups.

We also compared the codon pairing motifs (i.e., the set of codons that paired within a gene) across each taxonomic group. For example, a gene that has identical codon pairing for AAA and AAT would have a motif of {AAA, AAT}, regardless of how many times AAA or AAT paired. We found that fewer than 10% of codon pairing motifs were identified in multiple species in most taxonomic groups (see S1–S10 Figs in S1 File). Bacteria had the most repeated codon pairing motifs (13.7%) and fungi had the fewest repeated motifs (0.7%).

S3 Text in S1 File describes the frequency of codon pairing in each taxonomic group, with S11–S19 Figs in S1 File showing boxplots of the percentage of genes that have codon pairing for each codon. We further analyzed the number of codons that paired within each gene. We counted the number of codon pairing motifs that included 1, 2, 3,…, 61 codons and report the distribution for each taxonomic group in S20‒S29 Figs in S1 File. In most taxonomic groups, each motif contains ~10–40 codons. However, bacteria, archaea, and viruses are more likely to have fewer codons in each motif, while vertebrates typically have more codons in each motif.

We quantified the frequency of repeated motifs by counting the number of times each motif was used in each taxonomic group. S30‒S39 Figs in S1 File show the distribution of repeated motif frequencies in each taxonomic group. In most taxonomic groups, most repeated motifs are repeated 1–20 times with a steep decreasing slope as the motif is repeated more frequently. However, in archaea, the number of times a motif repeats quickly decreases between 1–30 and then the slope increases until 61 before sharply dropping to near zero. We also found that although the total number of codon pairings is highly correlated with gene length (R-squared = 0.973–1.0; see S40‒S48 Figs in S1 File), the number of codons that pair at least once in each gene is not correlated with gene length (R-squared = 0.0–0.08; see S49‒S57 Figs in S1 File). Therefore, the binary encoding of codon pairing is not driven by gene length. The scripts we used to create each supplementary Fig can be found at https://github.com/ridgelab/codon_pairing/supplementary_graphs.

Since we used a distance-based approach for the alignment-free method, we tested for saturation in each of the taxonomic groups by graphing the computed distance against the taxonomic distance of the compared species. As the taxonomic distance increases, we expect the computed distance to also increase. However, computed distances of 1.0 indicate that full saturation has occurred for the metric and additional species cannot be differentiated using that method. S58‒S66 Figs in S1 File show saturation often occurred for identical codon pairing, but the distances for co-tRNA and combined methods rarely fully saturated. Therefore, the co-tRNA and combined alignment-free methods have sufficient diversity of codon pairing motifs to recover the large phylogenies in our analyses, but the identical codon pairing alignment-free method may suffer from long branch attraction.

Additionally, we calculated the retention index of codon pairing for the parsimony method. A retention index of 1.0 indicates that the recovered tree contains no reversals, parallel gains, or parallel losses. We conducted 1 000 random permutations of species placement on the OTL tree topology, calculated the retention index of codon pairing for each of the random permutations, and then plotted the average codon pairing retention index from the OTL against the distribution of the average retention index of the permutated trees for each taxonomic group in S67‒S72 Figs in S1 File. If codon pairing has a strong phylogenetic signal, we would expect the observed average retention index to exceed the average from all permutated trees. The observed average retention index was significantly higher than the permutations in archaea, invertebrates, mammals, other vertebrates, and plants. The paraphyletic group, protozoa, was the only taxonomic group where a few permutations had slightly higher average retention indices. These results explain why the trees recovered using parsimony were largely congruent with the OTL.

## Discussion

We show that both identical and co-tRNA codon pairing are phylogenetically conserved across all domains of life. We further illustrate that combining identical and co-tRNA codon pairing improves the concordance of recovered phylogenies with the NCBI taxonomy and the OTL in most taxonomic groups. This comprehensive analysis shows that codon pairing is a novel phylogenetic character state that can be used in conjunction with other phylogenomic methods. Additionally, we provide tools for quickly analyzing thousands of species using our provided framework.

As opposed to common ortholog-based techniques that use shared character states to infer phylogenies, identical and co-tRNA codon pairing analyze sequence features that are associated with gene expression. Since gene expression plays a crucial role in adaptive divergence and ecological speciation [39], and codon pairing affects gene expression, we propose that detectable patterns in codon pairing not only inform phylogeny, but may track phenotypic variation between species. We show that codon pairing alone can recover phylogenies that are comparable to other alignment-free or maximum likelihood approaches, and patterns in codon pairing are largely conserved between species.

In some instances, codon pairing (or lack of codon pairing) might be due to protein structure instead of translational efficiency. Arginine (Arg) is very positively charged and highly repulsive to other like-charged amino acids. Although rarely pairing compared to other amino acid residues, arginine pairing is essential to some protein-protein interactions and occurs more frequently than expected by random chance [40]. In protein folding, coiled-coil interfaces often make asparagine (Asn)-Asn conformations that face away from the hydrophobic core [41]. Since coiled-coil proteins are present throughout all domains of life and occur in upwards of 10% of the proteome [42], it is likely that they play a nontrivial role in affecting codon pairing. Our analysis of codon pairing confirms that asparagine pairing occurs much more frequently than arginine pairing. These interactions suggest that asparagine and arginine pairing conservation might be based on structure instead of codon translational efficiency. However, the structural implications of codon usage do not rule out the additional effects of tRNA composition. Interestingly, although many species use only tRNA GTT [43],which may contribute to the bias for AAC codons and AAC codon pairing, AAT actually pairs more frequently in invertebrates, plants, protozoa, and viruses (see S11‒S19 Figs in S1 File), suggesting that structural implications may drive codon pairing in addition to tRNA composition.

Leucine zipper T cell receptors have the highest expression values [44]. Furthermore, the leucine zipper is a 60–80 amino acid protein domain that allows for faster gene expression, sequence-specific DNA-binding, and dimerization [45]. Our results show that leucin-encoding codons are among the most commonly paired codons. However, leucine-encoding CTA pairs significantly less frequently than other leucine-encoding codons. Further exploration into CTA interactions with other leucine-encoding codons may help determine why CTA pairs much less frequently.

Although co-tRNA codon pairing is less prominent in prokaryotes than in eukaryotes [26, 32, 33], we show that identical codon pairing and co-tRNA codon pairing are both phylogenetically conserved in all domains of life. However, we also show that using an alignment-free framework, the most congruent vertebrate and plant phylogenies are generally recovered using only identical codon pairing. Similarly, the parsimony method recovered the most congruent mammal phylogeny using only identical codon pairing. However, parsimony used only co-tRNA codon pairing in plants and the combined approach in non-mammalian vertebrates to recover the most congruent phylogenies. We show that although identical and co-tRNA codon pairing do not occur in equal frequencies, they are both phylogenetically conserved. We also show that combining identical and co-tRNA codon pairing recovers phylogenies that most support established phylogenies in seven out of ten taxonomic groups.

We recognize that systematic biases likely exist in RefSeq and may affect the results of our analyses. However, all algorithms are subject to the same limitations, and codon pairing performs comparably to these other algorithms. Furthermore, our filtering criteria for parsimony requires species to contain at least 5% of orthologs and orthologs to be called in at least 5% of the species to limit the effects of missing data. The number of species and parsimony-informative characters remaining after filtering are shown in S1-S6 Tables in S1 File. We opted to use this Big Data approach because we were interested in macro trends of codon pairing that we were able to identify through this comprehensive analysis. Furthermore, although codon pairing affects translational efficiency, the underlying mechanism governing codon pairing biases, like other codon usage biases, could be mutational biases or selection. Codon aversion biases, codon composition biases, mutational biases, and tRNA abundance might be the main source of the observed phylogenetic signal, although codon pairing appears to track that signal.

We used codon pairing to assess the controversial placement of falcons or pigeons as sister taxa to Neoaves, which was least supported by Shen, Hittinger [46]. The two phylogenies that we tested are found in S1 and S2 Phylogenies. We analyzed codon pairings using the conditional probability of the observed character states tracking a phylogeny, as established by Miller, McKinnon [23]. Our analyses add additional support to the current placement of pigeons on the Open Tree of Life as sister taxa to Neoaves because the conditional probability of codon pairings tracking the Open Tree of Life are higher than the probability of codon pairings tracking the alternative placement of falcons (see S20 Table in S1 File). Therefore, we propose keeping the current placement of falcons on the Open Tree of Life.

In taxonomic groups that have well-documented orthologous relationships, we show that codon pairing recovers parsimony trees that are largely congruent with the OTL and the NCBI taxonomy. Since maximum likelihood has been widely used to establish the reference phylogenies that we used for our comparisons, it is unsurprising that in the most established taxonomic groups, such as vertebrates, maximum likelihood recovers trees that are most congruent with the references. However, in plants and protozoa, the parsimony analysis elucidates a phylogenetic signal using only codon pairing that is sufficient to recover more congruent trees with the OTL and the NCBI taxonomy than maximum likelihood. Given the high degree of congruence between the established phylogenies, phylogenies recovered using other techniques, and the trees recovered using only codon pairing, we propose that codon pairing should be considered in future phylogenomic analyses.

## Materials and methods

### Data collection and processing

We downloaded all reference genomes and annotations from NCBI [47–49] in September, 2017. Reference genomes were used because they represent the most commonly accepted nucleotides in each species[48, 49]. We used the coding sequences (CDS) from the longest isoform of each gene, and we removed genes with previously-annotated exceptions (e.g., translational exception, unclassified transcription discrepancy, suspected errors, partial genes, etc.). A total of 23 428 species were divided into the following taxonomic groups based on RefSeq annotations, with some overlap between bacteria and viruses: 418 archaea, 15 063 bacteria, 234 fungi, 149 invertebrates, 89 plants, 75 protozoa, 107 mammalian vertebrates, 123 other vertebrates, and 7 233 viruses. While invertebrates, other vertebrates, and protozoa are paraphyletic outgroups, we opted to maintain these species classifications to facilitate analyses between different studies that use RefSeq. Furthermore, all algorithms will be subject to the same potential biases associated with analyzing paraphyletic groups.

### Accounting for differences in ribosomal footprint

Estimates of the ribosome footprint vary drastically and can range from 15 nucleotides (5 codons) to about 45 nucleotides (15 codons) with a commonly accepted length of 28 nucleotides (about nine codons) [50]. Since codon pairing requires at least two codons, we examined pairing lengths (i.e., a sliding window) of 2–11 codons. This technique allows for variations in the ribosomal footprint among different taxonomic groups and can determine if codon pairing is dispersed throughout the ribosomal footprint or is more phylogenetically conserved at a smaller window size.

### Calculating identical and co-tRNA codon pairing

For both the parsimony and alignment-free methods, we encoded identical codon pairings, co-tRNA codon pairings, and either identical and co-tRNA codon pairings with a binary representation (i.e., if a codon paired within a gene, it was given a value of '1' regardless of the number of times the pairing occurred). We determined which codons used identical codon pairing for each gene by adding each codon that occurred multiple times within the sliding window to a set of codons for that gene. Similarly, we created a set of amino acids for co-tRNA codon pairings for each gene by adding the amino acid product of the paired non-identical codons that encode that amino acid to the ordered set. Since the combined approach can use either identical or co-tRNA codon pairing, we calculated combined pairing by translating the gene sequence and identifying amino acids that paired within the ribosome window, adding each residue occurring multiple times in the sliding window to a set.

### Alignment-free codon pairing calculation

**Fig 3:**
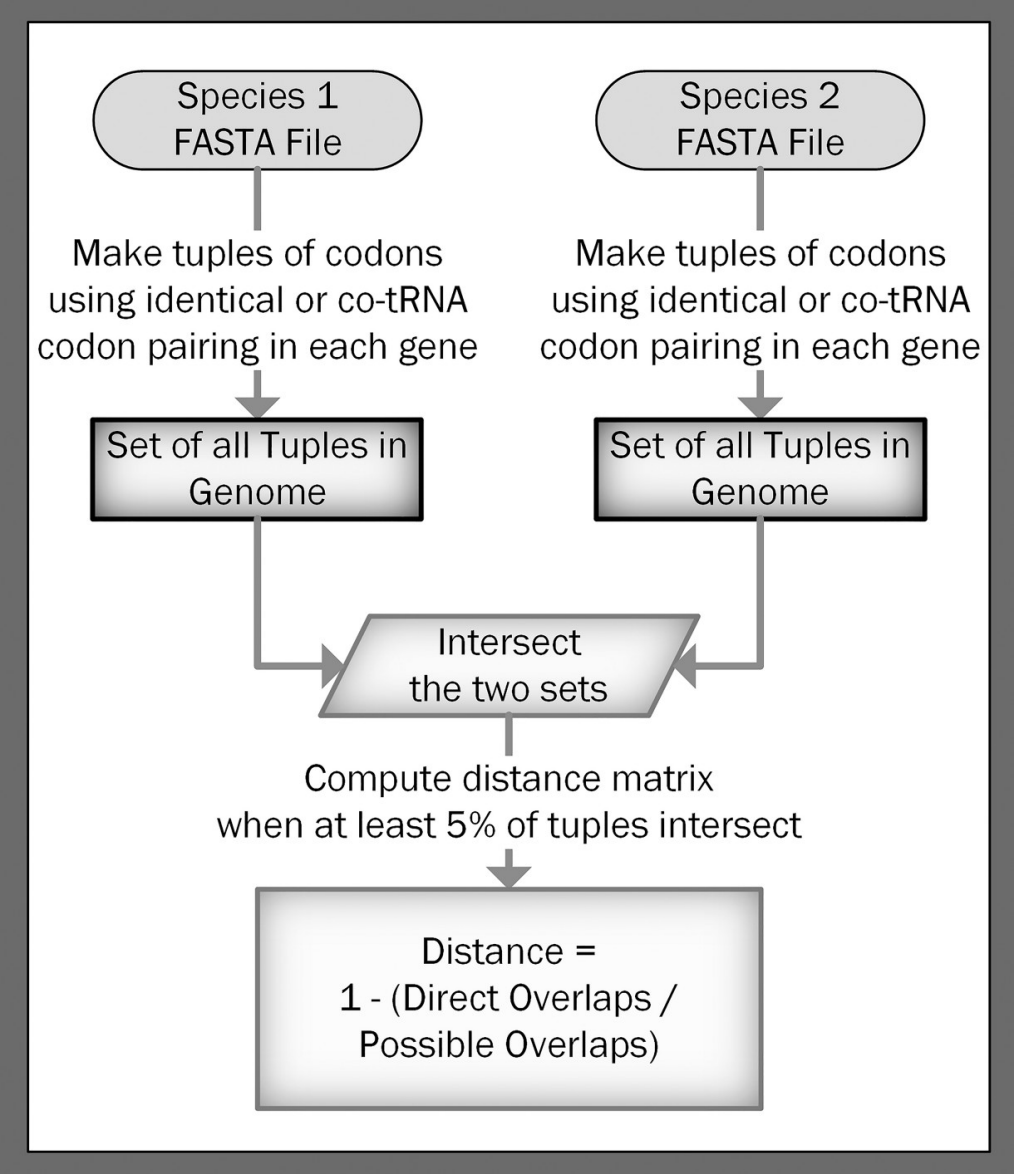
Process to calculate the distance matrix. Starting with the coding sequences of each gene in a species (FASTA file), codons that use codon pairing within the ribosomal footprint are included in a tuple that is then added to a set for that species. Sets of tuples are intersected to calculate the distance between species. These distances are then added to a distance matrix that can be used to recover phylogenies.

We present three alignment-free methods to calculate a distance matrix: 1) based on identical codon pairing, 2) based on co-tRNA codon pairing, and 3) based on a combination of either identical or co-tRNA codon pairing. Although genes must be assembled, orthologous relationships are not required or used in the distance matrix calculation. All three methods use a binary (occurs or does not occur) representation of codon pairing within a gene. First, if identical codon pairing occurs anywhere within a gene, the codons are added to a set for that gene. If co-tRNA codon pairing or the combined approach is selected, then amino acids are added to a set if they occur two or more times within the ribosomal footprint anywhere in the gene. Next, the sets are alphabetized and converted to a tuple (immutable list) so they can be added to a set for the entire species. This process is repeated for each gene within a species until all gene pairings have been made into tuples and added to a set for the species. We repeat this process for each species until all species have a set of tuples representing the codons (or amino acids) that are pairing within at least one gene. Finally, we calculate the distances between each species in a pairwise manner. This process is depicted in Fig 3.

Similar to the method used by Miller, McKinnon [12], the pairwise distance between two species, *A* and *B*, is calculated as one minus the relative similarity of the species. The relative similarity of the species is the number of overlapping tuples between the sets of tuples, *a* and *b*, from both species divided by the total number of tuples from *a* or *b* with the fewest number of tuples. This distance is given in Eq 1:

$$Dist(A,B)=1-\frac{\left|a\cap b\right|}{min(|a|,|b|)}$$ Eq 1

If the ratio of tuples in *a* and *b* does not exceed 5%, the species are assigned the maximum distance of 1.0. This filter limits small genome bias (e.g., without this cutoff, if one gene from a virus with two genes has the same codon pairing profile as a gene in a vertebrate with 20 000 genes, then the distance between the virus and the vertebrate would be 0.5). This process allows us to calculate a distance, with a maximum of 1.0, where more closely related species have a smaller distance because their overall codon pairing biases across all genes are more similar. A summary of alignment-free options is found in S4 Text in S1 File.

### Parsimony analysis

We used the NCBI gene annotations for our parsimony analysis, which includes annotations from species-specific nomenclature committees, NCBI staff curations, and the NCBI annotation pipeline. We used Python 3.5 to implement parsimony_pairing.py to create a character matrix of parsimony-informative codon pairings from a directory of FASTA files containing gene sequences for each species, one file per species. Each row in the matrix contains a record for a different species. Each column in the matrix represents a parsimony-informative codon (or amino acid) within a specific ortholog. For each species, each codon (or amino acid) in each ortholog is labelled '0' if it does not pair within a ribosomal window, '1' if it does pair, or '?' if the ortholog call is unavailable for that species.

**Fig 4:**
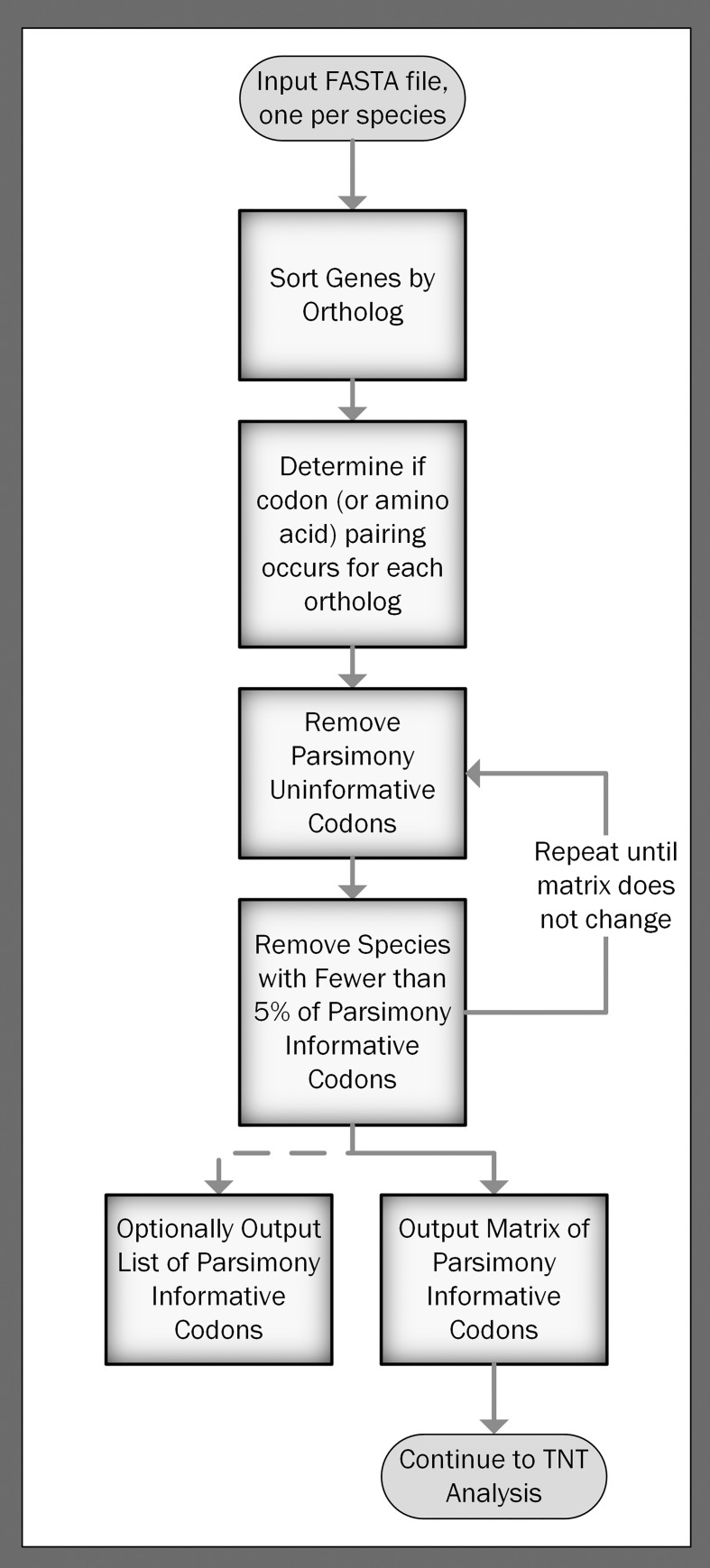
Flow chart for the parsimony analysis. We start with input FASTA files, one for each species. For each codon (or amino acid) within each ortholog, we assign a binary value of '0', '1', or '?' depending on if codon pairing for that codon (or amino acid) occurs. We then remove parsimony-uninformative characters. Next, we remove any species that do not contain at least 5% of the parsimony-informative codons, and we conduct the analysis only if at least 5% of the species pass the filter. Finally, we output the parsimony-informative character matrix for each codon (or amino acid) pairing to be used in a TNT analysis and an optional list of parsimony-informative characters.

To be considered parsimony-informative, each included ortholog was present in at least four species, each codon (or amino acid) paired in at least one species, and each codon (or amino acid) did not pair in at least one species. We further required all species to contain at least 5% of all the parsimony-informative codons (or amino acids) to limit the effect of missing data. We created this character matrix and a key file containing an ordered list of each parsimony-informative codon (or amino acid) that was included in the matrix in a single step at runtime (see Fig 4). The following command demonstrates typical usage for identical codon pairing, where $[51] is the path to a directory containing one FASTA file per species, ${MATRIX} is the path to the output matrix, and ${KEYS} is the path to the output key file containing the ordered list of parsimony-informative codons.

python getPairingMatrix.py -id $[51] -o ${MATRIX} -oc ${KEYS}

A summary of program options is found in S5 Text in S1 File.

### Constructing phylogenetic trees using parsimony

We used Tree Analysis Using New Technology (TNT) [52] to recover phylogenetic trees using parsimony. We selected TNT based on its ability to handle large datasets and its fast tree-searching algorithms. We found up to 100 most parsimonious trees, saving multiple trees recovered using tree bisection reconnection (tbr) branch swapping [53]. A discussion on the maximum number of species that can be placed on a tree using this method is found in S6 Text in S1 File.

### Reference phylogenies

We inferred subtrees of each taxonomic group from both the OTL and the NCBI Taxonomy Browser for each taxonomic group. The OTL combines phylogenetic relationships reported in primary literature and contains a web application programming interface (API) that allows for querying the OTL database. Although the NCBI Taxonomy Browser gathers information from a variety of sources and is therefore not considered a primary source for taxonomic relationships, it contains more species than the OTL and provides added insights into our analyses. We use both phylogenies as reference trees to compare the alignment-free and parsimony trees obtained from codon pairing.

### Open Tree of Life

We used getOTLtree.py [12] to obtain reference trees for each taxonomic group from the OTL in a single step at runtime. This program utilizes the OTL application programming interface (API) to programmatically query the OTL database to first obtain OTL taxonomy identifiers (OTT ids) for each species and then query the OTL database to retrieve the reference tree for the species found. The program also allows users to select the correct domain of life when multiple OTT ids are found for a species (e.g., *Nannospalax galili* is currently listed in the OTL database as both a eukaryote and a bacterium). The output file contains the inferred reference tree from the OTL and a list of any species that the OTL did not include in the tree. We ran this program using the following command, where ${INPUT} is a list of species, and ${OUTPUT} is the output file:

python getOTLtree.py -i ${INPUT} -o ${OUTPUT}

### NCBI taxonomy browser

We used the NCBI Taxonomy Browser (https://www.ncbi.nlm.nih.gov/Taxonomy/CommonTree/wwwcmt.cgi) to download the taxonomical relationships of each taxonomic group in PHYLIP [54] format. We included unranked taxa to maximize the number of included species for each taxonomic group.

### Tree comparison

We assessed the accuracy of our identical, co-tRNA, and combined codon pairing methods by comparing the trees we recovered to the reference trees from the OTL and the NCBI taxonomy. While both the OTL and the NCBI taxonomy combine phylogenetic trees presented by a variety of sources that may include source-specific biases, both phylogenies facilitate large-scale analyses across large and diverse taxonomic groups. We determined the similarity between trees by using the ete-compare module from the Environment for Tree Exploration toolkit (ETE3) [55], which computes the percentage of branch similarity between two trees. A higher percentage of branch similarity indicates higher congruence between trees. The branch similarity method has a relatively low computational cost for large datasets, and it allows for unrooted tree comparisons and comparisons of trees with polytomies. For the parsimony analysis, if any taxonomic comparison produced more than one equally parsimonious tree, we computed the percentage of edge similarity between each generated tree and the reference tree. We then reported the average percent overlap of all comparisons. Descriptions of the other methods used in our comparisons are found in S7 Text in S1 File.

## Supporting information

S1 File (DOCX)
