# Supplementary material for: Codon Pairs are Phylogenetically Conserved: A comprehensive analysis of codon pairing conservation across the Tree of Life
Source: PLoS One. 2020 May 13;15(5):e0232260. doi: 10.1371/journal.pone.0232260 (PMC7219770; doi:10.1371/journal.pone.0232260)
Supplement: S1 File — (DOCX) [file pone.0232260.s001.docx]

**Supplementary Information for: Codon Pairs are Phylogenetically Conserved:**

**A comprehensive analysis of codon pairing conservation across the Tree of Life**

Justin B. Miller^1,+^, Lauren M. McKinnon^1,+^, Michael F. Whiting^1,2^, John S.K. Kauwe^1^, and Perry G. Ridge^1,*^

^1^Department of Biology, Brigham Young University, Provo, UT 84602, USA

^2^M.L. Bean Museum, Brigham Young University, Provo, UT 84602, USA

^+^Contributed equally to this work

^*^To whom correspondence should be sent: [*perry.ridge@byu.edu*](mailto:perry.ridge@byu.edu)

Table of Contents

[Text 6](#_Toc34217094)

[S1 Text: Species Filter for Parsimony and Maximum Likelihood 6](#_Toc34217095)

[S2 Text: Description of the Optimal Window Size 6](#_Toc34217096)

[S3 Text: Frequency of Codon Pairing Within Taxonomic Groups 6](#_Toc34217097)

[S4 Text: Summary of Alignment-free Options 7](#_Toc34217098)

[S5 Text: Summary of Parsimony Options 7](#_Toc34217099)

[S6 Text: Maximum Number of Species in the Phylogeny 7](#_Toc34217100)

[S7 Text: Summary of Other Methods Used for Comparison 8](#_Toc34217101)

[Tables 10](#_Toc34217102)

[Parsimony 10](#_Toc34217103)

[S1 Table: Number of species used in identical codon pairing 10](#_Toc34217104)

[S2 Table: Number of species used in co-tRNA codon pairing 10](#_Toc34217105)

[S3 Table: Number of species used in combined comparison 11](#_Toc34217106)

[S4 Table: Number of parsimony-informative codons used in identical codon pairing 11](#_Toc34217107)

[S5 Table: Number of parsimony-informative codons used in co-tRNA codon pairing 11](#_Toc34217108)

[S6 Table: Number of parsimony-informative codons used in the combined analysis 12](#_Toc34217109)

[S7 Table: Optimal window size and options for each taxonomic group 12](#_Toc34217110)

[Alignment-free Method 13](#_Toc34217111)

[S8 Table: Identical codon pairing percent overlap with the NCBI Taxonomy 13](#_Toc34217112)

[S9 Table: Identical codon pairing percent overlap with the OTL 13](#_Toc34217113)

[S10 Table: Both co-tRNA and identical codon pairing percent overlap with the NCBI Taxonomy 13](#_Toc34217114)

[S11 Table: Both co-tRNA and identical codon pairing percent overlap with the OTL 13](#_Toc34217115)

[S12 Table: Co-tRNA codon pairing percent overlap with the NCBI Taxonomy 14](#_Toc34217116)

[S13 Table: Co-tRNA codon pairing percent overlap with the OTL 14](#_Toc34217117)

[Parsimony Method 15](#_Toc34217118)

[S14 Table: Identical codon pairing percent overlap with the NCBI Taxonomy 15](#_Toc34217119)

[S15 Table: Identical codon pairing percent overlap with the OTL 15](#_Toc34217120)

[S16 Table: Combined codon pairing percent overlap with the NCBI Taxonomy 16](#_Toc34217121)

[S17 Table: Combined codon pairing percent overlap with the OTL 16](#_Toc34217122)

[S18 Table: Co-tRNA codon pairing percent overlap with the NCBI Taxonomy 17](#_Toc34217123)

[S19 Table: Co-tRNA codon pairing percent overlap with the OTL 17](#_Toc34217124)

[S20 Table: Chi-squared analysis of the placement of *Falco* on the Tree of Life 17](#_Toc34217125)

[Figures 18](#_Toc34217126)

[Overlapping and Unique Motifs 18](#_Toc34217127)

[S1 Figure: All Species 18](#_Toc34217128)

[S2 Figure: Archaea 18](#_Toc34217129)

[S3 Figure: Bacteria 18](#_Toc34217130)

[S4 Figure: Fungi 19](#_Toc34217131)

[S5 Figure: Invertebrates 19](#_Toc34217132)

[S6 Figure: Mammals 19](#_Toc34217133)

[S7 Figure: Other Vertebrates 20](#_Toc34217134)

[S8 Figure: Plants 20](#_Toc34217135)

[S9 Figure: Protozoa 20](#_Toc34217136)

[S10 Figure: Viruses 21](#_Toc34217137)

[Codon Pairing Frequency 22](#_Toc34217138)

[S11 Figure: Archaea 22](#_Toc34217139)

[S12 Figure: Bacteria 22](#_Toc34217140)

[S13 Figure: Fungi 22](#_Toc34217141)

[S14 Figure: Invertebrates 23](#_Toc34217142)

[S15 Figure: Mammals 23](#_Toc34217143)

[S16 Figure: Other Vertebrates 23](#_Toc34217144)

[S17 Figure: Plants 24](#_Toc34217145)

[S18 Figure: Protozoa 24](#_Toc34217146)

[S19 Figure: Viruses 24](#_Toc34217147)

[Number of Codons Included in Each Motif 25](#_Toc34217148)

[S20 Figure: All Species 25](#_Toc34217149)

[S21 Figure: Archaea 25](#_Toc34217150)

[S22 Figure: Bacteria 26](#_Toc34217151)

[S23 Figure: Fungi 26](#_Toc34217152)

[S24 Figure: Invertebrates 27](#_Toc34217153)

[S25 Figure: Mammals 27](#_Toc34217154)

[S26 Figure: Other Vertebrates 28](#_Toc34217155)

[S27 Figure: Plants 28](#_Toc34217156)

[S28 Figure: Protozoa 29](#_Toc34217157)

[S29 Figure: Viruses 29](#_Toc34217158)

[Number of Times Motifs are Repeated 30](#_Toc34217159)

[S30 Figure: All Species 30](#_Toc34217160)

[S31 Figure: Archaea 31](#_Toc34217161)

[S32 Figure: Bacteria 31](#_Toc34217162)

[S33 Figure: Fungi 32](#_Toc34217163)

[S34 Figure: Invertebrates 32](#_Toc34217164)

[S35 Figure: Mammals 33](#_Toc34217165)

[S36 Figure: Other Vertebrates 33](#_Toc34217166)

[S37 Figure: Plants 34](#_Toc34217167)

[S38 Figure: Protozoa 35](#_Toc34217168)

[S39 Figure: Viruses 35](#_Toc34217169)

[Number of Codon Pairs versus Gene Length 36](#_Toc34217170)

[S40 Figure: Archaea 36](#_Toc34217171)

[S41 Figure: Bacteria 37](#_Toc34217172)

[S42 Figure: Fungi 38](#_Toc34217173)

[S43 Figure: Invertebrates 39](#_Toc34217174)

[S44 Figure: Mammals 40](#_Toc34217175)

[S45 Figure: Other Vertebrates 41](#_Toc34217176)

[S46 Figure: Plants 42](#_Toc34217177)

[S47 Figure: Protozoa 43](#_Toc34217178)

[S48 Figure: Viruses 44](#_Toc34217179)

[Number of Codons in Pairing Motif versus Gene Length 45](#_Toc34217180)

[S49 Figure: Archaea 45](#_Toc34217181)

[S50 Figure: Bacteria 46](#_Toc34217182)

[S51 Figure: Fungi 47](#_Toc34217183)

[S52 Figure: Invertebrates 48](#_Toc34217184)

[S53 Figure: Mammals 49](#_Toc34217185)

[S54 Figure: Other Vertebrates 50](#_Toc34217186)

[S55 Figure: Plants 51](#_Toc34217187)

[S56 Figure: Protozoa 52](#_Toc34217188)

[S57 Figure: Viruses 53](#_Toc34217189)

[Saturation Analysis 54](#_Toc34217190)

[S58 Figure: Archaea 54](#_Toc34217191)

[S59 Figure: Bacteria 55](#_Toc34217192)

[S60 Figure: Fungi 56](#_Toc34217193)

[S61 Figure: Invertebrates 57](#_Toc34217194)

[S62 Figure: Mammals 58](#_Toc34217195)

[S63 Figure: Other Vertebrates 59](#_Toc34217196)

[S64 Figure: Plants 60](#_Toc34217197)

[S65 Figure: Protozoa 61](#_Toc34217198)

[S66 Figure: Viruses 62](#_Toc34217199)

[Retention Index Analysis 63](#_Toc34217200)

[S67 Figure: Archaea 63](#_Toc34217201)

[S68 Figure: Invertebrates 64](#_Toc34217202)

[S69 Figure: Mammals 65](#_Toc34217203)

[S70 Figure: Other Vertebrates 65](#_Toc34217204)

[S71 Figure: Plants 66](#_Toc34217205)

[S72 Figure: Protozoa 67](#_Toc34217206)

[Phylogenies 68](#_Toc34217207)

[S1 Phylogeny: Phylogeny with falcons as sister taxa to Neoaves in other vertebrates 68](#_Toc34217208)

[S2 Phylogeny: Phylogeny with pigeons as sister taxa to Neoaves in other vertebrates 69](#_Toc34217209)

[Supplementary References 70](#_Toc34217210)

# Text

## S1 Text: Species Filter for Parsimony and Maximum Likelihood

Parsimony and maximum likelihood used similar numbers of species in each analysis. A stricter filter was applied to the parsimony analysis than the maximum likelihood analysis, which required the parsimony character matrix to include at least 5% of the parsimony-informative characters. After this filter was applied, we required that at least 5% of the total number of species be included in the analysis (e.g., if 100 species were analyzed, at least five species must pass the preprocessing step for the taxonomic group to be included). Applying this filter removed the results from all species, bacteria, and viruses from both the parsimony and maximum likelihood analyses. This filter also removed fungi from the parsimony analysis.

## S2 Text: Description of the Optimal Window Size

We define the minimum optimal window size as the smallest window size to recover the most congruent phylogeny when compared to the reference. Across all taxonomic groups, the minimum optimal window size was relatively small. Averaged for all minimum optimal window sizes that produced the highest congruence with the OTL, parsimony had a mean minimum optimal window size of 4.000 with a sample standard deviation of 3.033. The alignment-free method had a mean minimum optimal window size of 3.500 with a sample standard deviation of 1.509.

## S3 Text: Frequency of Codon Pairing Within Taxonomic Groups

The frequency of identical codon pairing was also calculated to determine if systematic biases in pairing based on the codon exist. We counted the number of genes in a species that used identical codon pairing for each codon. We then calculated the frequency of codon pairing for each codon by dividing the number of genes with identical codon pairing for that codon by the total number of genes in that same species. Bacteria, archaea, protozoa, and viruses had very wide distributions of codon pairing frequencies. Fungi and invertebrates had narrower distributions of codon pairing frequencies. Mammals, plants, and other vertebrates had very narrow distributions of codon pairing frequencies. Narrow distributions indicate less variability in codon pairing between species within the taxonomic group. Taxonomic groups have similar patterns of pairing usage (i.e., if a codon pairs frequently in one taxonomic group, it also pairs frequently in other taxonomic groups), although mammals have the least variation between species. Excluding stop codons, fewer genes have at least one instance of arginine pairing (occurs in ~20-25% of genes) than any other codon. Genes are most likely to have at least one instance of asparagine and leucine codon pairing (occurs in ~60-75% of genes), except leucine-encoding CTA, which pairs in only ~20-25% of genes.

## S4 Text: Summary of Alignment-free Options

We implemented pairing_distance.py in Python 3.5 to calculate the distance matrix based on the codon pairing algorithm outlined above. We provide several additional options for pairing_distance.py to give users greater flexibility in their research. Input FASTA files can be provided either as a list (standard bash expansion) with the -i option, or included in a single directory with the -id option. The program automatically handles gzipped compressed files with the .gz or .gzip file extension or uncompressed data with any other file extension. The output distance matrix by default is written to standard out, although an output file can be provided through the -o option. Although all available processing cores are used by default to calculate the distance, this can be modified with the -t option. RNA sequences can also be provided using the -rna flag. The -l option allows the user to specify an alternative codon table, with the standard codon table being used by default. By default, the ribosome footprint is set to nine codons, although this option can be modified using -f. In the same program, we also provide a flag, -c, to allow users to use co-tRNA codon pairing instead of identical codon pairing and the -b flag to signify both identical and co-tRNA codon pairing. These options are explained in more detail in the accompanying README file found in the GitHub repository: <https://github.com/ridgelab/codon_pairing/tree/master/alignment_free>.

## S5 Text: Summary of Parsimony Options

We provide the same options in parsimony_pairing.py as the alignment-free method, with a few notable exceptions. In addition to the options described in the alignment-free section, –oc optionally indicates the path to an output file containing the ordered parsimony-informative codons included in the character matrix. Optionally, –on will use a numbering system to create names for the species instead of using the names of the input files. This option is most useful when file names are very long or do not correlate to the species names.

## S6 Text: Maximum Number of Species in the Phylogeny

We aimed to determine how phylogenies recovered using identical codon pairing and/or co-tRNA pairing compare to traditional methods (i.e., parsimony and maximum likelihood) and other alignment-free methods. First, we determined the theoretical maximum number of character states for each gene using codon pairing in order to determine the maximum number of species we can differentiate using this technique. For identical codon pairing, there are 61 possible pairing combinations (64 codons – 3 stop codons), meaning each gene can separate a maximum of 2^61^ = 2.306 x 10^18^ species. For co-tRNA codon pairing, there are 18 amino acids that use more than one codon, meaning there are 18 possible pairing combinations. Using co-tRNA codon paring, each gene can separate a maximum of 2^18^ = 262,144 species. Using the combined approach, there are 20 possible pairing combinations, one for each of the 20 amino acids. This approach allows each gene to separate a maximum of 2^20^ = 1,048,576 species. Since orthologous genes are conserved between species, closely related species share a higher number of codon pairings than more distantly related species. Our parsimony analyses show that closely related species often have more similar codon pairings than distantly related species, which allows us to accurately recover phylogenetic relationships.

## S7 Text: Summary of Other Methods Used for Comparison

Comparison with Maximum Likelihood

We used the same maximum likelihood validation results as previously reported in Miller, McKinnon (1). The ortholog-based maximum likelihood technique first compiled all NCBI gene annotations and subsampled the most commonly used orthologs in each taxonomic group, where all gene annotations must be unique within a given species. Next, they used Clustal Omega [2] to perform a multiple sequence alignment (MSA) on each orthologous gene cluster. Finally, IQ-TREE [3] was used to perform a maximum likelihood analysis on the combined MSA super-matrix from all orthologs. ETE3 was used to compare the recovered phylogenies to the OTL and the NCBI taxonomy. Miller, McKinnon (1) excluded bacteria and viruses from their analyses because of the lack of orthologs spanning a sufficient number of species.

Comparison with Codon Aversion Motifs

Codon aversion motifs (CAM) are sets of codons that are not used within genes [1]. They have also been used to recover phylogenies using alignment-free techniques. Since our method using codon pairing is also a codon-based method, we included CAM in our comparisons to determine if the phylogenetic signal is more congruent with established phylogenies using codon use/aversion or codon pairing. We use the results reported in Miller, McKinnon (1) for our comparisons.

Comparison with Feature Frequency Profiles

Comparisons were also done with a k-mer based alignment-free phylogenomic approach, Feature Frequency Profiles (FFP) [4, 5]. The FFP method works by counting shared k-mers between species, with more directly overlapping k-mer counts being associated with closer species relatedness. Since we use the same dataset as previously reported in Miller, McKinnon (1), we also use their FFP validation set to compare the congruence of FFP with the OTL and the NCBI taxonomy.

Comparison with CVTree

CVTree is an alignment-free approach that uses composition vectors to calculate the frequency of words of a given length. The algorithm normalizes the composition vector frequencies by expected frequencies from random chance. Species with similar word frequencies would be considered to be closely related. We use the CVTree validation set previously reported in Miller, McKinnon (1).

Comparison with Average Common Substring (ACS)

ACS is an alignment-free approach that determines distances by calculating the average match lengths of substrings. The algorithm finds the longest substring at each index of a sequence that is also contained in a second sequence. It then calculates the average length of the matching substrings. We use the ACS validation set reported in Miller, McKinnon (1).

Comparison with andi

Andi is another alignment-free algorithm that searches two genes for areas of exact matches, referred to as anchors, that enclose mismatch areas. It then compares the mismatch areas to compute a distance between species. We use the andi validation set reported in Miller, McKinnon (1).

Comparison with Filtered Spaced-word Matches

Filtered Spaced-word Matches (FSWM) is an alignment-free approach that is similar to andi because it also searches for matching-spaced words between sequences. FSWM differs from andi by filtering out matches that could have been caused by random chance. We used the FSWM validation set reported in Miller, McKinnon (1).

# Tables

## Parsimony

### S1 Table: Number of species used in identical codon pairing

| Window Size | 2 | 3 | 4 | 5 | 6 | 7 | 8 | 9 | 10 | 11 | Average | Total Number of Species |
| --- | --- | --- | --- | --- | --- | --- | --- | --- | --- | --- | --- | --- |
| Taxonomic Group |  |  |  |  |  |  |  |  |  |  |  |  |
| Archaea | 106 | 95 | 95 | 95 | 95 | 95 | 95 | 95 | 95 | 95 | 96.1 | 418 |
| Fungi | 19 | 9 | 13 | 9 | 9 | 19 | 9 | 13 | 9 | 9 | 11.8 | 234 |
| Invertebrates | 65 | 55 | 57 | 55 | 55 | 55 | 63 | 55 | 57 | 55 | 57.2 | 149 |
| Plants | 60 | 60 | 60 | 61 | 61 | 61 | 61 | 61 | 61 | 61 | 60.7 | 89 |
| Protozoa | 15 | 15 | 15 | 16 | 17 | 16 | 16 | 20 | 15 | 15 | 16 | 75 |
| Mammals | 97 | 97 | 97 | 97 | 97 | 97 | 97 | 97 | 97 | 97 | 97 | 107 |
| Other vertebrates | 114 | 114 | 114 | 114 | 114 | 114 | 114 | 114 | 114 | 114 | 114 | 123 |
| Viruses | 168 | 137 | 152 | 188 | 177 | 174 | 174 | 220 | 176 | 184 | 175 | 7 233 |

### S2 Table: Number of species used in co-tRNA codon pairing

| Window Size | 2 | 3 | 4 | 5 | 6 | 7 | 8 | 9 | 10 | 11 | Average | Total Number of Species |
| --- | --- | --- | --- | --- | --- | --- | --- | --- | --- | --- | --- | --- |
| Taxonomic Group |  |  |  |  |  |  |  |  |  |  |  |  |
| Archaea | 107 | 107 | 107 | 107 | 107 | 107 | 107 | 107 | 107 | 106 | 106.9 | 418 |
| Fungi | 9 | 10 | 10 | 20 | 9 | 20 | 9 | 14 | 13 | 10 | 12.7 | 234 |
| Invertebrates | 65 | 65 | 66 | 55 | 65 | 57 | 65 | 55 | 57 | 55 | 60.5 | 149 |
| Plants | 61 | 59 | 59 | 59 | 59 | 59 | 59 | 59 | 59 | 59 | 59.2 | 89 |
| Protozoa | 17 | 16 | 19 | 17 | 15 | 18 | 15 | 16 | 16 | 16 | 16.5 | 75 |
| Mammals | 97 | 97 | 97 | 97 | 97 | 97 | 97 | 97 | 97 | 97 | 97 | 107 |
| Other vertebrates | 114 | 114 | 114 | 114 | 114 | 114 | 114 | 114 | 114 | 114 | 114 | 123 |
| Viruses | 282 | 279 | 262 | 247 | 243 | 256 | 256 | 245 | 236 | 244 | 255 | 7 233 |

### S3 Table: Number of species used in combined comparison

| Window Size | 2 | 3 | 4 | 5 | 6 | 7 | 8 | 9 | 10 | 11 | Average | Total Number of Species |
| --- | --- | --- | --- | --- | --- | --- | --- | --- | --- | --- | --- | --- |
| Taxonomic Group |  |  |  |  |  |  |  |  |  |  |  |  |
| Archaea | 96 | 100 | 96 | 100 | 101 | 101 | 101 | 95 | 105 | 106 | 100.1 | 418 |
| Fungi | 13 | 13 | 10 | 14 | 11 | 11 | 13 | 10 | 11 | 14 | 12 | 234 |
| Invertebrates | 55 | 57 | 57 | 69 | 93 | 58 | 57 | 65 | 65 | 58 | 63.4 | 149 |
| Plants | 61 | 61 | 59 | 59 | 59 | 59 | 59 | 59 | 59 | 59 | 59.4 | 89 |
| Protozoa | 15 | 15 | 25 | 27 | 17 | 18 | 21 | 20 | 20 | 21 | 19.9 | 75 |
| Mammals | 97 | 97 | 97 | 97 | 97 | 97 | 97 | 97 | 97 | 97 | 97 | 107 |
| Other vertebrates | 114 | 114 | 114 | 114 | 114 | 114 | 114 | 114 | 114 | 114 | 114 | 123 |
| Viruses | 199 | 224 | 180 | 190 | 190 | 180 | 278 | 262 | 261 | 257 | 222.1 | 7 233 |

### S4 Table: Number of parsimony-informative codons used in identical codon pairing

| Window Size | 2 | 3 | 4 | 5 | 6 | 7 | 8 | 9 | 10 | 11 | Average |
| --- | --- | --- | --- | --- | --- | --- | --- | --- | --- | --- | --- |
| Taxonomic Group |  |  |  |  |  |  |  |  |  |  |  |
| Archaea | 6151 | 8450 | 9902 | 10544 | 11035 | 11254 | 11518 | 11687 | 11664 | 11842 | 10404.7 |
| Fungi | N/A | N/A | N/A | N/A | N/A | N/A | N/A | N/A | N/A | N/A | N/A |
| Invertebrates | 794 | 988 | 1081 | 1160 | 1236 | 1263 | 1329 | 1427 | 1353 | 1423 | 1205.4 |
| Plants | 6230 | 8033 | 9036 | 9842 | 10153 | 10517 | 10607 | 10691 | 10725 | 10693 | 9652.7 |
| Protozoa | 12449 | 14864 | 16051 | 16253 | 16103 | 16171 | 15837 | 15838 | 15764 | 15532 | 15486.2 |
| Mammals | 197074 | 311796 | 319490 | 381908 | 404078 | 335058 | 398896 | 386949 | 436474 | 380879 | 355260.2 |
| Other vertebrates | 228194 | 277024 | 347408 | 388789 | 355121 | 376798 | 400771 | 380813 | 390161 | 15532 | 316061.1 |
| Viruses | 16622 | 23528 | 28145 | 28776 | 30176 | 30768 | 32248 | 31580 | 33374 | 33082 | 28829.9 |

### S5 Table: Number of parsimony-informative codons used in co-tRNA codon pairing

| Window Size | 2 | 3 | 4 | 5 | 6 | 7 | 8 | 9 | 10 | 11 | Average |
| --- | --- | --- | --- | --- | --- | --- | --- | --- | --- | --- | --- |
| Taxonomic Group |  |  |  |  |  |  |  |  |  |  |  |
| Archaea | 3293 | 3294 | 3087 | 2921 | 2783 | 2725 | 2689 | 2661 | 2560 | 2579 | 2859.2 |
| Fungi | N/A | N/A | N/A | N/A | N/A | N/A | N/A | N/A | N/A | N/A | N/A |
| Invertebrates | 418 | 461 | 475 | 455 | 450 | 429 | 422 | 428 | 410 | 382 | 433 |
| Plants | 3219 | 3188 | 3157 | 3082 | 2945 | 2940 | 2929 | 2819 | 2808 | 2723 | 2981 |
| Protozoa | 5415 | 5319 | 5020 | 4840 | 4589 | 4358 | 4198 | 4067 | 3976 | 2819 | 4460.1 |
| Mammals | 94018 | 10195 | 93587 | 93627 | 82729 | 74208 | 70805 | 71310 | 69678 | 55666 | 71582.3 |
| Other vertebrates | 90872 | 86618 | 82126 | 73534 | 74286 | 70704 | 63995 | 62945 | 60569 | 57241 | 72289 |
| Viruses | 11409 | 12103 | 11556 | 11421 | 11248 | 11068 | 10812 | 10550 | 10325 | 10115 | 11060.7 |

### S6 Table: Number of parsimony-informative codons used in the combined analysis

| Window Size | 2 | 3 | 4 | 5 | 6 | 7 | 8 | 9 | 10 | 11 | Average |
| --- | --- | --- | --- | --- | --- | --- | --- | --- | --- | --- | --- |
| Taxonomic Group |  |  |  |  |  |  |  |  |  |  |  |
| Archaea | 2823 | 2568 | 2292 | 2105 | 1974 | 1851 | 1756 | 1577 | 1612 | 1527 | 2008.5 |
| Fungi | N/A | N/A | N/A | N/A | N/A | N/A | N/A | N/A | N/A | N/A | N/A |
| Invertebrates | 463 | 464 | 417 | 411 | 359 | 351 | 319 | 302 | 293 | 272 | 365.1 |
| Plants | 3236 | 2813 | 2571 | 2347 | 2214 | 2085 | 1977 | 1849 | 1789 | 1709 | 2259 |
| Protozoa | 4488 | 3603 | 3021 | 2813 | 2381 | 2311 | 2122 | 1951 | 1862 | 1730 | 2628.2 |
| Mammals | 72029 | 64087 | 56125 | 44448 | 44984 | 37830 | 45077 | 41319 | 34711 | 35801 | 44089.2 |
| Other vertebrates | 6937 | 61754 | 48110 | 45789 | 43517 | 41519 | 34718 | 33856 | 30895 | 27919 | 37501.4 |
| Viruses | 12737 | 12003 | 11587 | 10946 | 10352 | 9924 | 9691 | 9247 | 8921 | 8578 | 10398.6 |

### S7 Table: Optimal window size and options for each taxonomic group

| Taxonomic Group | Alignment-free identical codon pairing (I), co-tRNA codon pairing (C), or both (B) | Alignment-free minimum optimal window sizes | Maximum parsimony identical codon pairing (I), co-tRNA codon pairing (C), or both (B) | Maximum parsimony minimum optimal window sizes |
| --- | --- | --- | --- | --- |
| All | B | 2 | N/A | N/A |
| Archaea | B | 4 | B | 3 |
| Bacteria^*^ | B | 2 | N/A | N/A |
| Fungi | B | 5 | N/A | N/A |
| Invertebrates | B | 2 | B | 4 |
| Plants | I | 4 | C | 10 |
| Protozoa | B | 2 | B | 2 |
| Mammals | I | 6 | I | 2 |
| Other vertebrates | I | 5 | B | 3 |
| Viruses^*^ | B | 3 | N/A | N/A |

## Alignment-free Method

### S8 Table: Identical codon pairing percent overlap with the NCBI Taxonomy

| Window Size | 2 | 3 | 4 | 5 | 6 | 7 | 8 | 9 | 10 | 11 |
| --- | --- | --- | --- | --- | --- | --- | --- | --- | --- | --- |
| Taxonomic Group |  |  |  |  |  |  |  |  |  |  |
| All | 90 | 90 | 90 | 90 | 90 | 90 | 90 | 90 | 90 | 90 |
| Archaea | 84 | 84 | 84 | 84 | 85 | 84 | 84 | 83 | 83 | 83 |
| Bacteria | 91 | 92 | 92 | 92 | 92 | 92 | 92 | 92 | 92 | 92 |
| Fungi | 74 | 73 | 74 | 74 | 74 | 75 | 75 | 74 | 74 | 74 |
| Invertebrates | 72 | 72 | 72 | 72 | 73 | 74 | 72 | 72 | 72 | 71 |
| Plants | 75 | 78 | 81 | 80 | 80 | 79 | 80 | 80 | 80 | 80 |
| Protozoa | 79 | 78 | 79 | 79 | 77 | 78 | 76 | 77 | 77 | 77 |
| Mammals | 92 | 92 | 94 | 94 | 95 | 94 | 93 | 91 | 90 | 91 |
| Other Vertebrates | 81 | 85 | 86 | 87 | 85 | 83 | 81 | 82 | 82 | 81 |
| Viruses | 89 | 89 | 89 | 90 | 90 | 91 | 91 | 91 | 90 | 90 |

### S9 Table: Identical codon pairing percent overlap with the OTL

| Window Size | 2 | 3 | 4 | 5 | 6 | 7 | 8 | 9 | 10 | 11 |
| --- | --- | --- | --- | --- | --- | --- | --- | --- | --- | --- |
| Taxonomic Group |  |  |  |  |  |  |  |  |  |  |
| All | 83 | 83 | 83 | 83 | 83 | 83 | 83 | 83 | 83 | 83 |
| Archaea | 78 | 78 | 77 | 78 | 78 | 77 | 77 | 77 | 77 | 76 |
| Bacteria | 85 | 85 | 85 | 85 | 85 | 85 | 85 | 85 | 85 | 85 |
| Fungi | 71 | 72 | 71 | 71 | 71 | 72 | 72 | 71 | 71 | 72 |
| Invertebrates | 63 | 64 | 63 | 63 | 65 | 65 | 64 | 63 | 62 | 61 |
| Plants | 68 | 71 | 73 | 72 | 72 | 71 | 74 | 72 | 74 | 74 |
| Protozoa | 69 | 70 | 70 | 70 | 68 | 70 | 69 | 69 | 69 | 68 |
| Mammals | 83 | 85 | 86 | 86 | 89 | 87 | 86 | 84 | 84 | 84 |
| Other Vertebrates | 68 | 71 | 72 | 73 | 72 | 71 | 69 | 70 | 70 | 69 |

### S10 Table: Both co-tRNA and identical codon pairing percent overlap with the NCBI Taxonomy

| Window Size | 2 | 3 | 4 | 5 | 6 | 7 | 8 | 9 | 10 | 11 |
| --- | --- | --- | --- | --- | --- | --- | --- | --- | --- | --- |
| Taxonomic Group |  |  |  |  |  |  |  |  |  |  |
| All | 91 | 91 | 91 | 91 | 91 | 91 | 91 | 91 | 91 | 91 |
| Archaea | 88 | 87 | 88 | 88 | 87 | 87 | 87 | 87 | 87 | 87 |
| Bacteria | 93 | 93 | 93 | 92 | 92 | 92 | 92 | 92 | 92 | 92 |
| Fungi | 77 | 77 | 77 | 78 | 77 | 77 | 76 | 75 | 76 | 75 |
| Invertebrates | 78 | 76 | 75 | 75 | 75 | 74 | 74 | 74 | 74 | 73 |
| Plants | 74 | 73 | 73 | 73 | 73 | 71 | 72 | 70 | 71 | 70 |
| Protozoa | 80 | 79 | 79 | 78 | 77 | 77 | 77 | 77 | 78 | 78 |
| Mammals | 89 | 87 | 87 | 87 | 85 | 85 | 85 | 85 | 84 | 84 |
| Other Vertebrates | 79 | 78 | 77 | 80 | 81 | 80 | 81 | 81 | 80 | 80 |
| Viruses | 90 | 91 | 91 | 91 | 91 | 91 | 91 | 91 | 91 | 91 |

### S11 Table: Both co-tRNA and identical codon pairing percent overlap with the OTL

| Window Size | 2 | 3 | 4 | 5 | 6 | 7 | 8 | 9 | 10 | 11 |
| --- | --- | --- | --- | --- | --- | --- | --- | --- | --- | --- |
| Taxonomic Group |  |  |  |  |  |  |  |  |  |  |
| All | 84 | 84 | 84 | 84 | 84 | 84 | 84 | 84 | 84 | 84 |
| Archaea | 81 | 81 | 82 | 81 | 81 | 81 | 81 | 81 | 80 | 81 |
| Bacteria | 86 | 86 | 86 | 86 | 86 | 86 | 86 | 86 | 86 | 86 |
| Fungi | 74 | 75 | 74 | 76 | 74 | 74 | 73 | 73 | 74 | 73 |
| Invertebrates | 69 | 67 | 67 | 65 | 66 | 65 | 64 | 65 | 64 | 64 |
| Plants | 65 | 65 | 65 | 65 | 63 | 62 | 62 | 61 | 62 | 61 |
| Protozoa | 70 | 69 | 70 | 69 | 67 | 67 | 67 | 67 | 67 | 67 |
| Mammals | 79 | 76 | 78 | 78 | 76 | 76 | 76 | 77 | 75 | 75 |
| Other Vertebrates | 67 | 66 | 65 | 66 | 66 | 67 | 67 | 68 | 67 | 66 |

### S12 Table: Co-tRNA codon pairing percent overlap with the NCBI Taxonomy

| Window Size | 2 | 3 | 4 | 5 | 6 | 7 | 8 | 9 | 10 | 11 |
| --- | --- | --- | --- | --- | --- | --- | --- | --- | --- | --- |
| Taxonomic Group |  |  |  |  |  |  |  |  |  |  |
| All | 90 | 90 | 90 | 90 | 90 | 90 | 90 | 90 | 90 | 90 |
| Archaea | 83 | 83 | 83 | 83 | 83 | 82 | 82 | 82 | 83 | 82 |
| Bacteria | 91 | 91 | 91 | 91 | 91 | 91 | 91 | 91 | 91 | 91 |
| Fungi | 72 | 72 | 72 | 73 | 72 | 71 | 71 | 70 | 71 | 70 |
| Invertebrates | 70 | 70 | 70 | 70 | 70 | 70 | 69 | 69 | 69 | 69 |
| Plants | 70 | 69 | 69 | 68 | 68 | 68 | 68 | 69 | 67 | 68 |
| Protozoa | 76 | 75 | 77 | 75 | 73 | 74 | 73 | 74 | 73 | 75 |
| Mammals | 87 | 87 | 86 | 87 | 85 | 86 | 87 | 84 | 84 | 84 |
| Other Vertebrates | 76 | 76 | 76 | 76 | 76 | 76 | 75 | 74 | 77 | 75 |
| Viruses | 90 | 90 | 90 | 90 | 90 | 90 | 90 | 90 | 90 | 90 |

### S13 Table: Co-tRNA codon pairing percent overlap with the OTL

| Window Size | 2 | 3 | 4 | 5 | 6 | 7 | 8 | 9 | 10 | 11 |
| --- | --- | --- | --- | --- | --- | --- | --- | --- | --- | --- |
| Taxonomic Group |  |  |  |  |  |  |  |  |  |  |
| All | 82 | 82 | 82 | 82 | 82 | 82 | 82 | 82 | 82 | 82 |
| Archaea | 75 | 76 | 75 | 76 | 76 | 75 | 75 | 75 | 76 | 76 |
| Bacteria | 84 | 84 | 85 | 85 | 85 | 85 | 85 | 85 | 85 | 85 |
| Fungi | 69 | 70 | 71 | 70 | 70 | 69 | 69 | 69 | 69 | 69 |
| Invertebrates | 62 | 61 | 61 | 61 | 60 | 60 | 60 | 61 | 60 | 59 |
| Plants | 62 | 60 | 60 | 60 | 59 | 59 | 59 | 60 | 59 | 59 |
| Protozoa | 70 | 68 | 69 | 68 | 65 | 66 | 65 | 66 | 65 | 66 |
| Mammals | 77 | 77 | 76 | 77 | 76 | 77 | 77 | 76 | 75 | 75 |
| Other Vertebrates | 66 | 65 | 63 | 64 | 64 | 65 | 65 | 64 | 65 | 63 |

## Parsimony Method

### S14 Table: Identical codon pairing percent overlap with the NCBI Taxonomy

| Window Size | 2 | 3 | 4 | 5 | 6 | 7 | 8 | 9 | 10 | 11 |
| --- | --- | --- | --- | --- | --- | --- | --- | --- | --- | --- |
| Taxonomic Group |  |  |  |  |  |  |  |  |  |  |
| All | N/A | N/A | N/A | N/A | N/A | N/A | N/A | N/A | N/A | N/A |
| Archaea | 84 | 83 | 87 | 88 | 86 | 85 | 86 | 84 | 85 | 84 |
| Bacteria | N/A | N/A | N/A | N/A | N/A | N/A | N/A | N/A | N/A | N/A |
| Fungi | N/A | N/A | N/A | N/A | N/A | N/A | N/A | N/A | N/A | N/A |
| Invertebrates | 58 | 63 | 67 | 65 | 67 | 64 | 61 | 65 | 62 | 63 |
| Plants | 80 | 80 | 82 | 82 | 81 | 82 | 84 | 79 | 79 | 79 |
| Protozoa | 81 | 81 | 81 | 77 | 73 | 77 | 77 | 68 | 81 | 81 |
| Mammals | 96 | 96 | 95 | 94 | 93 | 92 | 93 | 94 | 94 | 93 |
| Other Vertebrates | 91 | 91 | 90 | 90 | 91 | 90 | 90 | 91 | 90 | 91 |
| Viruses | N/A | N/A | N/A | N/A | N/A | N/A | N/A | N/A | N/A | N/A |

### S15 Table: Identical codon pairing percent overlap with the OTL

| Window Size | 2 | 3 | 4 | 5 | 6 | 7 | 8 | 9 | 10 | 11 |
| --- | --- | --- | --- | --- | --- | --- | --- | --- | --- | --- |
| Taxonomic Group |  |  |  |  |  |  |  |  |  |  |
| All | N/A | N/A | N/A | N/A | N/A | N/A | N/A | N/A | N/A | N/A |
| Archaea | 84 | 84 | 86 | 86 | 86 | 86 | 85 | 84 | 85 | 84 |
| Bacteria | N/A | N/A | N/A | N/A | N/A | N/A | N/A | N/A | N/A | N/A |
| Fungi | N/A | N/A | N/A | N/A | N/A | N/A | N/A | N/A | N/A | N/A |
| Invertebrates | 55 | 58 | 62 | 61 | 64 | 59 | 57 | 61 | 59 | 60 |
| Plants | 77 | 75 | 77 | 78 | 78 | 77 | 79 | 75 | 74 | 75 |
| Protozoa | 68 | 68 | 68 | 66 | 63 | 66 | 66 | 58 | 68 | 68 |
| Mammals | 90 | 89 | 87 | 86 | 86 | 86 | 86 | 88 | 87 | 87 |
| Other Vertebrates | 77 | 76 | 75 | 76 | 76 | 76 | 75 | 76 | 77 | 77 |

### S16 Table: Combined codon pairing percent overlap with the NCBI Taxonomy

| Window Size | 2 | 3 | 4 | 5 | 6 | 7 | 8 | 9 | 10 | 11 |
| --- | --- | --- | --- | --- | --- | --- | --- | --- | --- | --- |
| Taxonomic Group |  |  |  |  |  |  |  |  |  |  |
| All | N/A | N/A | N/A | N/A | N/A | N/A | N/A | N/A | N/A | N/A |
| Archaea | 87 | 90 | 89 | 89 | 88 | 87 | 88 | 88 | 89 | 87 |
| Bacteria | N/A | N/A | N/A | N/A | N/A | N/A | N/A | N/A | N/A | N/A |
| Fungi | N/A | N/A | N/A | N/A | N/A | N/A | N/A | N/A | N/A | N/A |
| Invertebrates | 63 | 65 | 71 | 63 | 63 | 63 | 63 | 63 | 62 | 64 |
| Plants | 77 | 77 | 79 | 77 | 84 | 81 | 78 | 80 | 77 | 78 |
| Protozoa | 87 | 85 | 67 | 66 | 76 | 75 | 67 | 67 | 61 | 66 |
| Mammals | 95 | 92 | 95 | 91 | 94 | 92 | 92 | 93 | 94 | 94 |
| Other Vertebrates | 93 | 94 | 92 | 93 | 93 | 93 | 91 | 91 | 91 | 91 |
| Viruses | N/A | N/A | N/A | N/A | N/A | N/A | N/A | N/A | N/A | N/A |

### S17 Table: Combined codon pairing percent overlap with the OTL

| Window Size | 2 | 3 | 4 | 5 | 6 | 7 | 8 | 9 | 10 | 11 |
| --- | --- | --- | --- | --- | --- | --- | --- | --- | --- | --- |
| Taxonomic Group |  |  |  |  |  |  |  |  |  |  |
| All | N/A | N/A | N/A | N/A | N/A | N/A | N/A | N/A | N/A | N/A |
| Archaea | 86 | 87 | 90 | 89 | 89 | 86 | 88 | 88 | 87 | 86 |
| Bacteria | N/A | N/A | N/A | N/A | N/A | N/A | N/A | N/A | N/A | N/A |
| Fungi | N/A | N/A | N/A | N/A | N/A | N/A | N/A | N/A | N/A | N/A |
| Invertebrates | 57 | 61 | 66 | 57 | 56 | 58 | 59 | 59 | 58 | 57 |
| Plants | 73 | 71 | 74 | 72 | 77 | 75 | 73 | 74 | 72 | 72 |
| Protozoa | 73 | 71 | 57 | 57 | 66 | 66 | 59 | 59 | 54 | 60 |
| Mammals | 87 | 86 | 86 | 85 | 87 | 85 | 84 | 85 | 85 | 85 |
| Other Vertebrates | N/A | N/A | N/A | N/A | N/A | N/A | N/A | N/A | N/A | N/A |

### S18 Table: Co-tRNA codon pairing percent overlap with the NCBI Taxonomy

| Window Size | 2 | 3 | 4 | 5 | 6 | 7 | 8 | 9 | 10 | 11 |
| --- | --- | --- | --- | --- | --- | --- | --- | --- | --- | --- |
| Taxonomic Group |  |  |  |  |  |  |  |  |  |  |
| All | N/A | N/A | N/A | N/A | N/A | N/A | N/A | N/A | N/A | N/A |
| Archaea | 88 | 88 | 86 | 86 | 83 | 84 | 84 | 83 | 85 | 82 |
| Bacteria | N/A | N/A | N/A | N/A | N/A | N/A | N/A | N/A | N/A | N/A |
| Fungi | N/A | N/A | N/A | N/A | N/A | N/A | N/A | N/A | N/A | N/A |
| Invertebrates | 59 | 60 | 59 | 60 | 59 | 58 | 59 | 59 | 58 | 59 |
| Plants | 76 | 82 | 80 | 80 | 80 | 82 | 81 | 80 | 84 | 80 |
| Protozoa | 38 | 77 | 74 | 75 | 86 | 74 | 86 | 77 | 81 | 81 |
| Mammals | 94 | 92 | 91 | 92 | 93 | 90 | 94 | 92 | 92 | 92 |
| Other Vertebrates | 92 | 91 | 91 | 91 | 90 | 90 | 91 | 91 | 90 | 90 |
| Viruses | N/A | N/A | N/A | N/A | N/A | N/A | N/A | N/A | N/A | N/A |

### S19 Table: Co-tRNA codon pairing percent overlap with the OTL

| Window Size | 2 | 3 | 4 | 5 | 6 | 7 | 8 | 9 | 10 | 11 |
| --- | --- | --- | --- | --- | --- | --- | --- | --- | --- | --- |
| Taxonomic Group |  |  |  |  |  |  |  |  |  |  |
| All | N/A | N/A | N/A | N/A | N/A | N/A | N/A | N/A | N/A | N/A |
| Archaea | 89 | 88 | 86 | 87 | 83 | 85 | 85 | 82 | 83 | 84 |
| Bacteria | N/A | N/A | N/A | N/A | N/A | N/A | N/A | N/A | N/A | N/A |
| Fungi | N/A | N/A | N/A | N/A | N/A | N/A | N/A | N/A | N/A | N/A |
| Invertebrates | 53 | 54 | 54 | 54 | 53 | 55 | 54 | 53 | 53 | 53 |
| Plants | 73 | 75 | 77 | 76 | 79 | 79 | 79 | 79 | 80 | 78 |
| Protozoa | 59 | 66 | 63 | 62 | 72 | 65 | 72 | 66 | 69 | 69 |
| Mammals | 86 | 85 | 85 | 86 | 85 | 84 | 86 | 84 | 85 | 84 |
| Other Vertebrates | 76 | 76 | 77 | 76 | 75 | 76 | 76 | 76 | 76 | 76 |

## S20 Table: Chi-squared analysis of the placement of *Falco* on the Tree of Life

| Type of Codon Pairing | Chi-squared OTL | Degrees of Freedom OTL | Chi-squared Falco Changed | Degrees of Freedom Falco Changed | Number of Significant Pairs OTL | Number of Significant Pairs Falco Changed |
| --- | --- | --- | --- | --- | --- | --- |
| Identical | 4.70x10^18^ | 2628 | 4.36x10^18^ | 2580 | 263 | 261 |
| Co-tRNA | 4.70x10^18^ | 2628 | 3.29x10^17^ | 2500 | 56 | 56 |
| Combined | 9.83x10^19^ | 2418 | 9.43x10^19^ | 2381 | 143 | 143 |

# Figures

## Overlapping and Unique Motifs

### S1 Figure: All Species


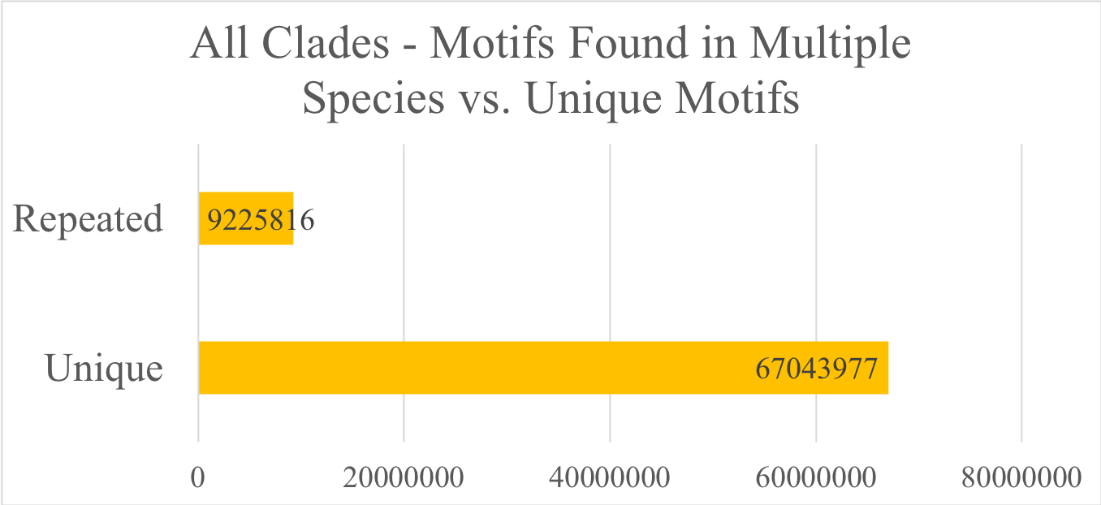


### S2 Figure: Archaea


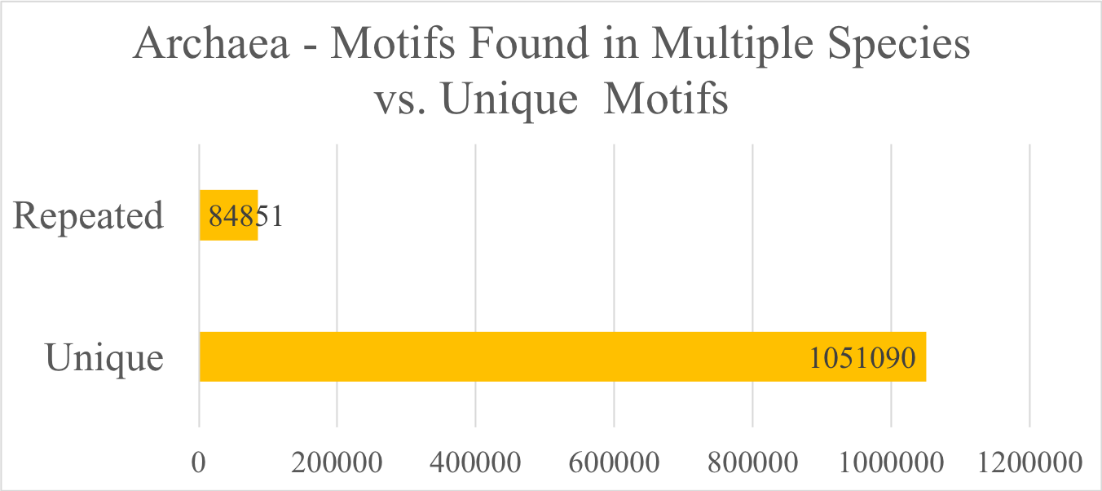


### S3 Figure: Bacteria


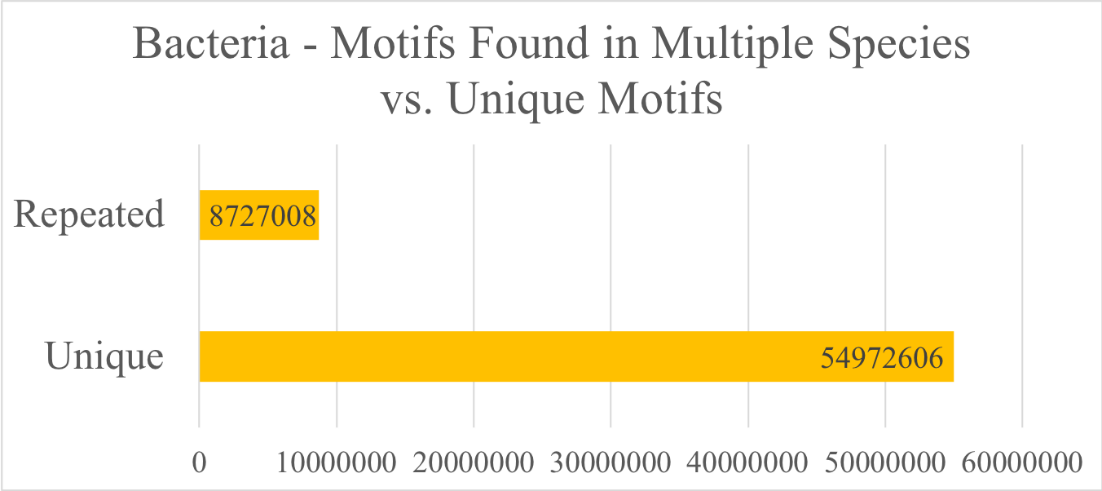


### S4 Figure: Fungi


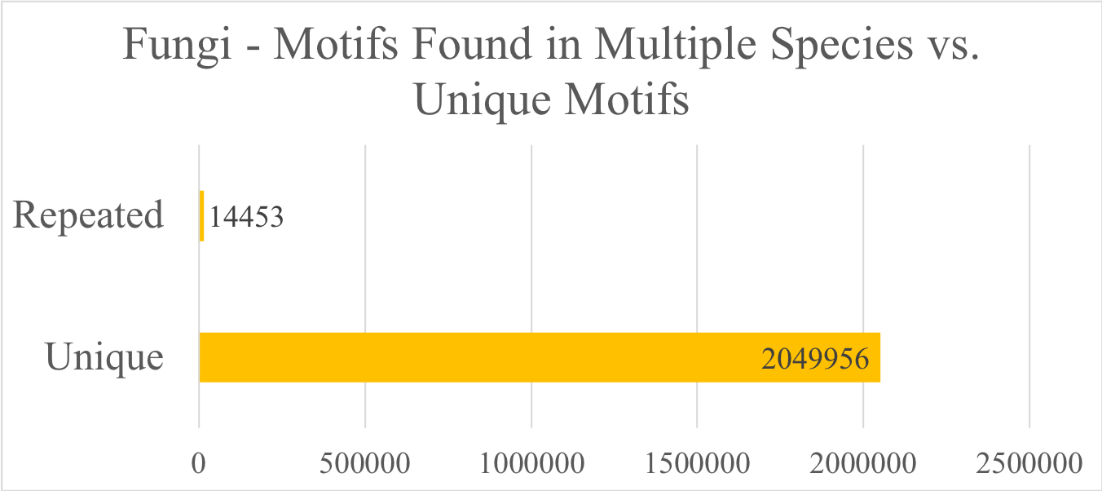


### S5 Figure: Invertebrates


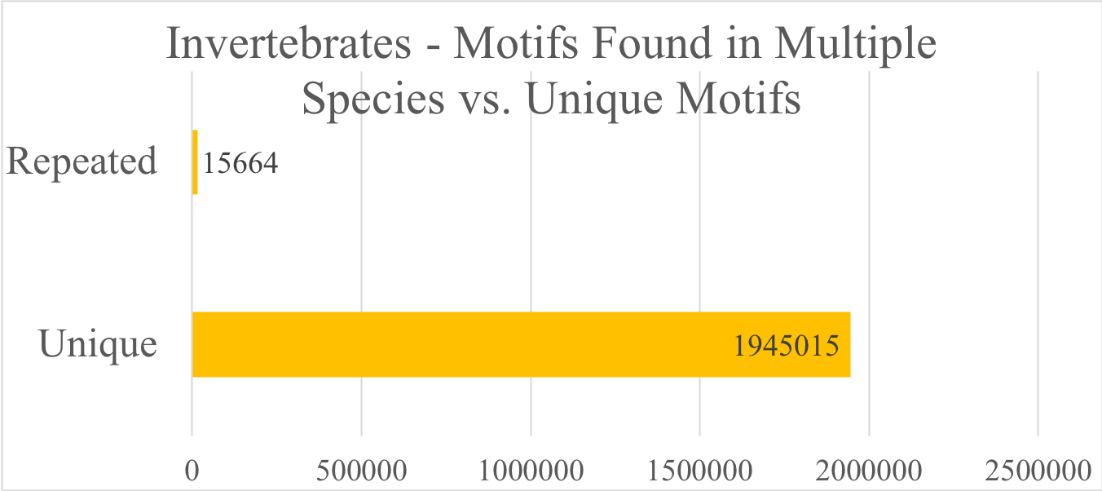


### S6 Figure: Mammals


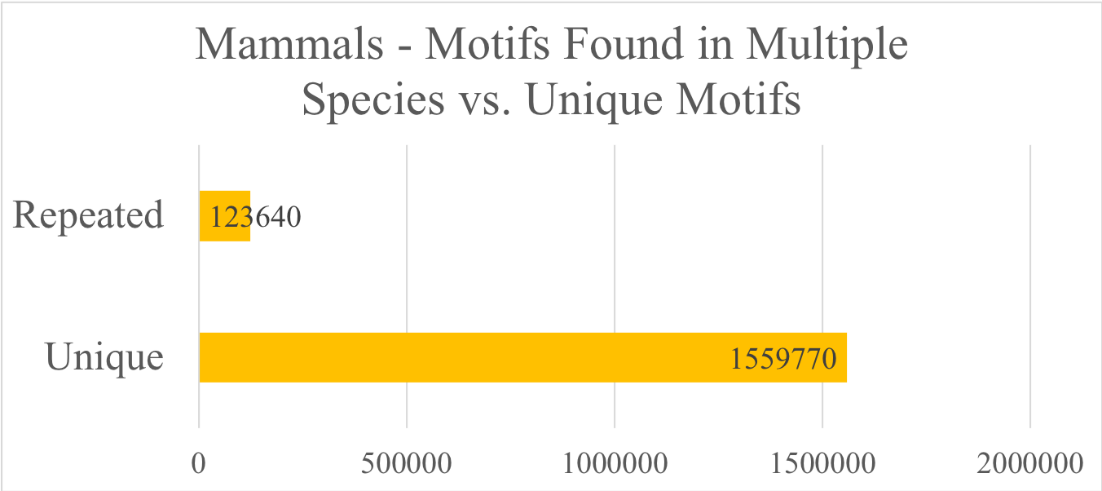


### S7 Figure: Other Vertebrates


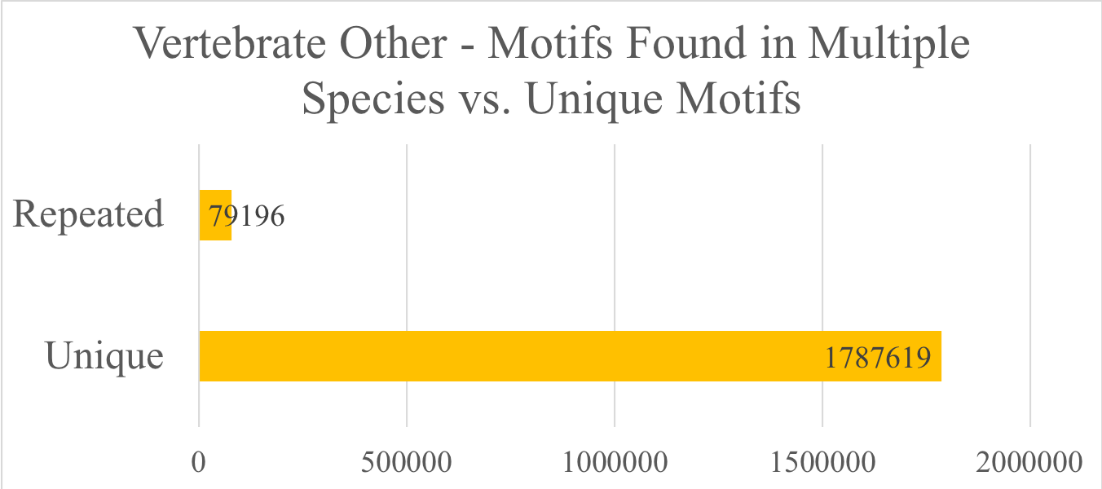


### S8 Figure: Plants


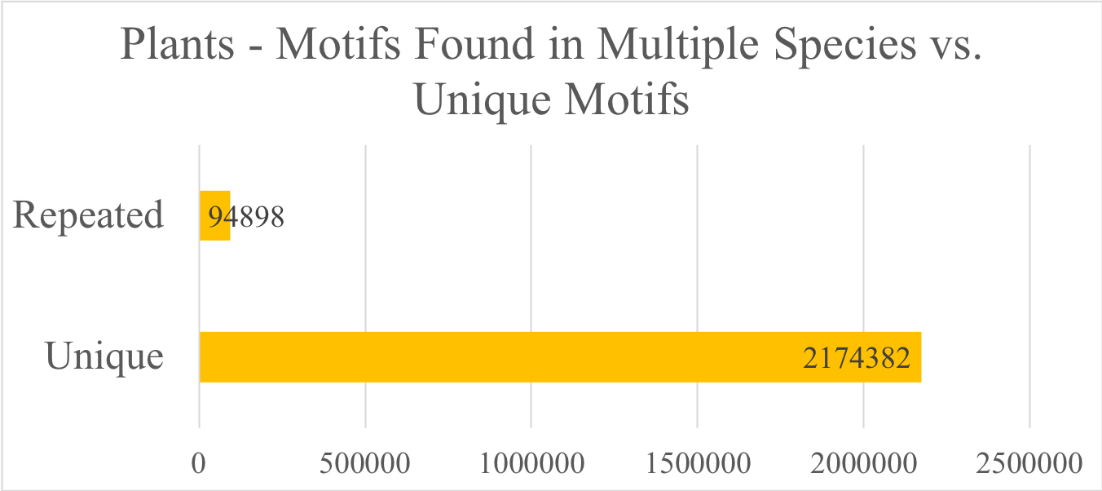


### S9 Figure: Protozoa


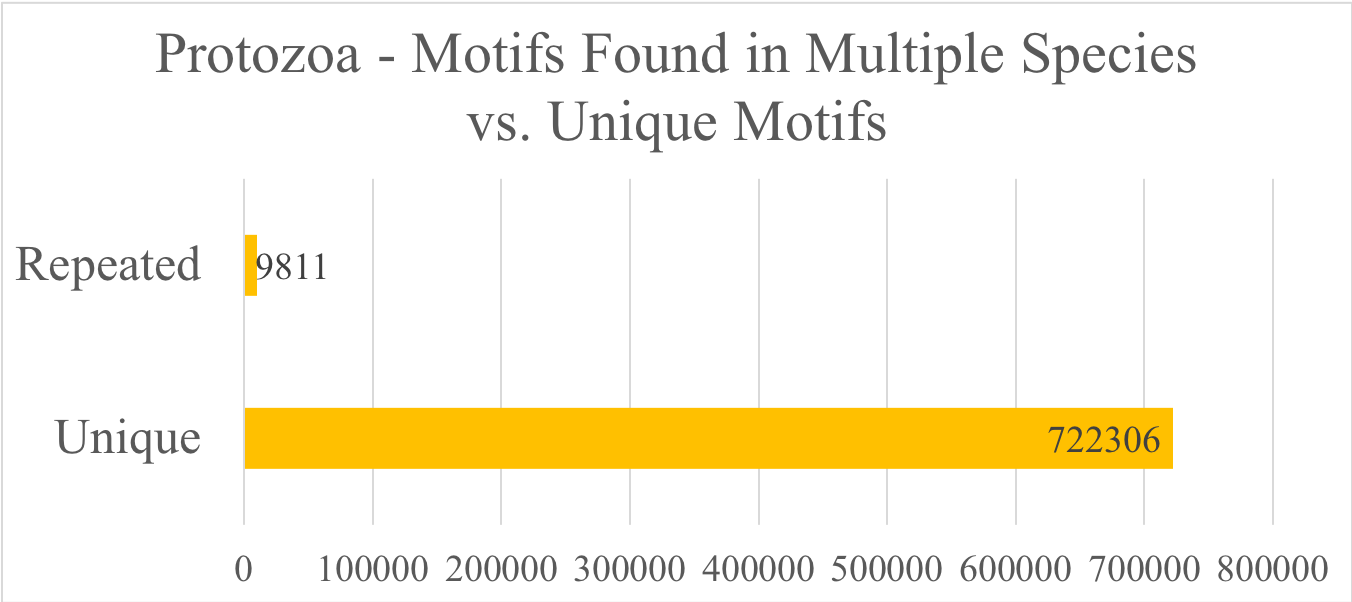


### S10 Figure: Viruses


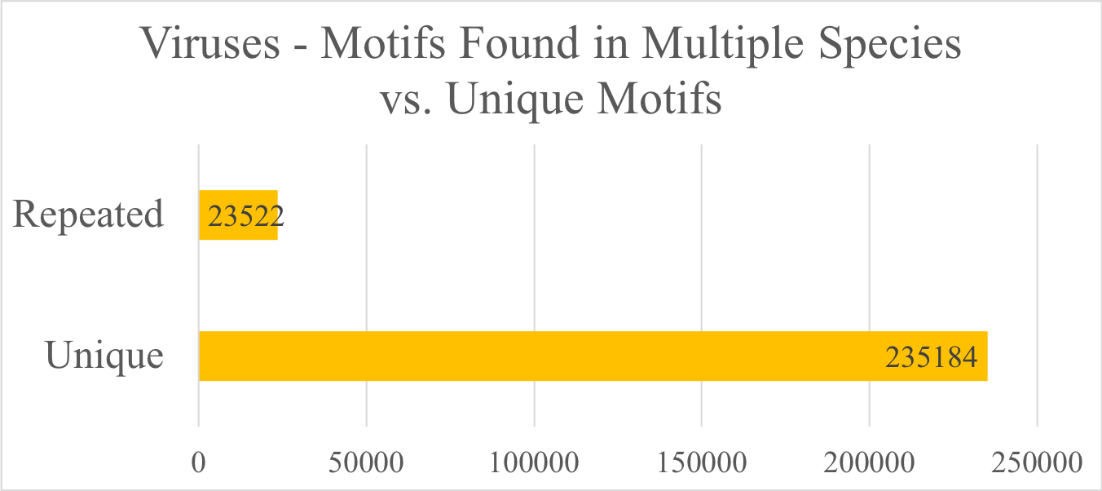


## Codon Pairing Frequency

### S11 Figure: Archaea


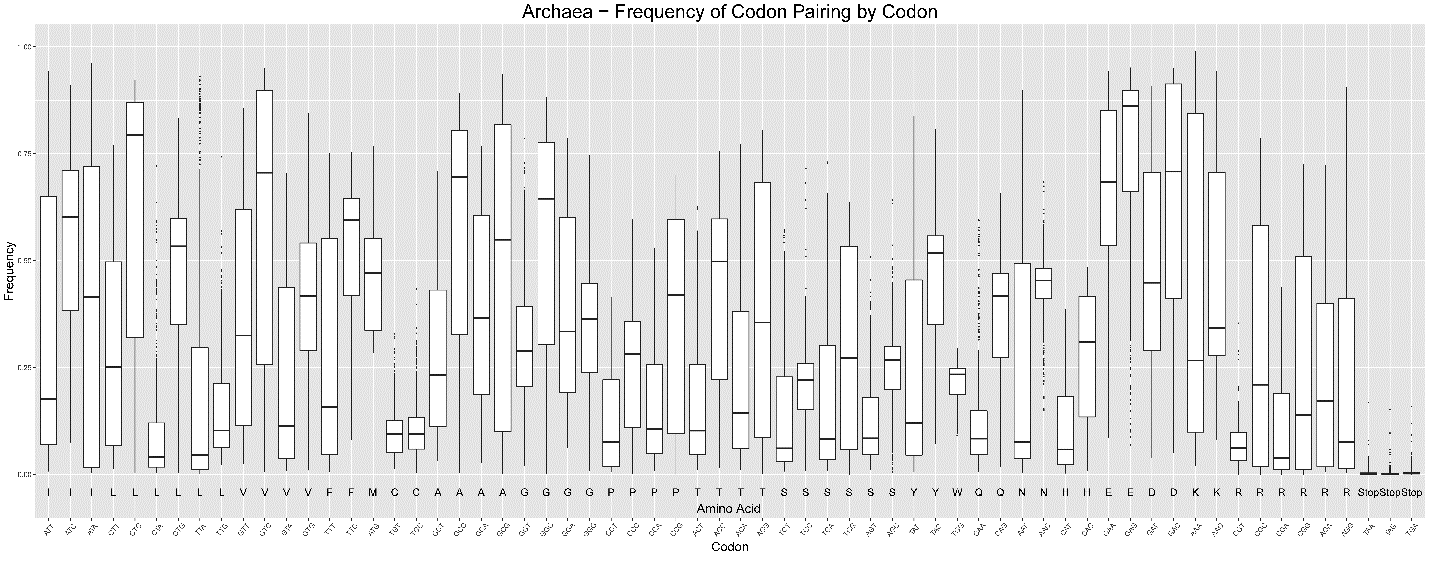


### S12 Figure: Bacteria


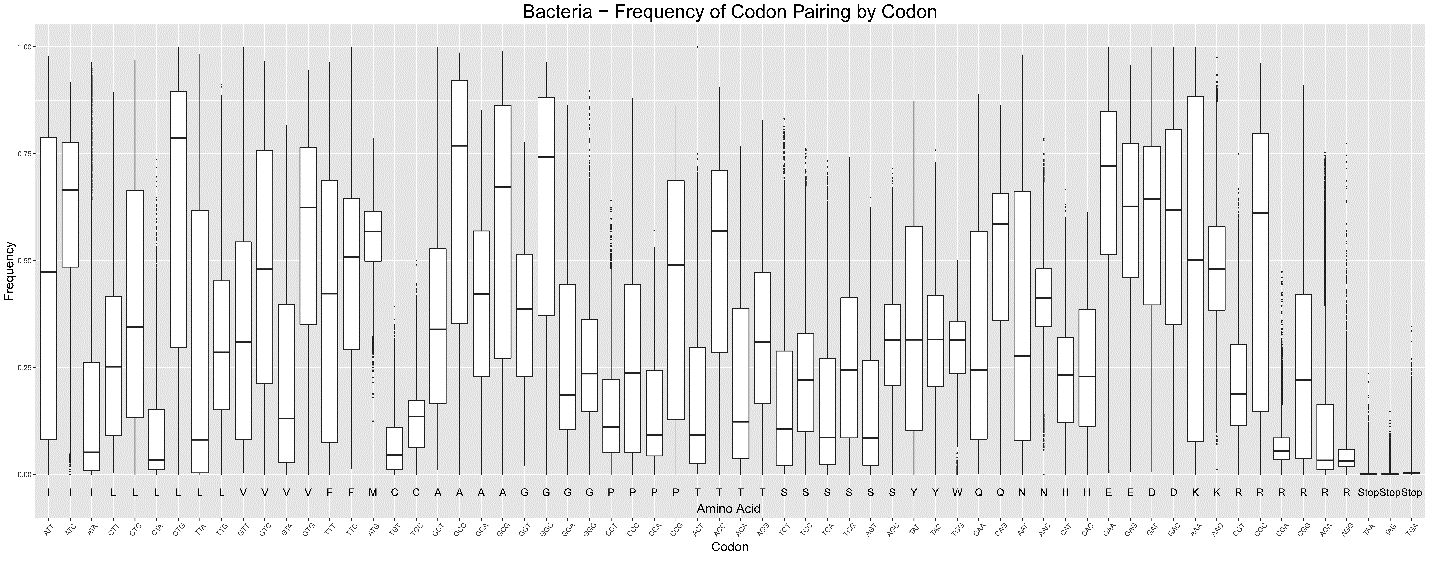


S13 Figure: Fungi
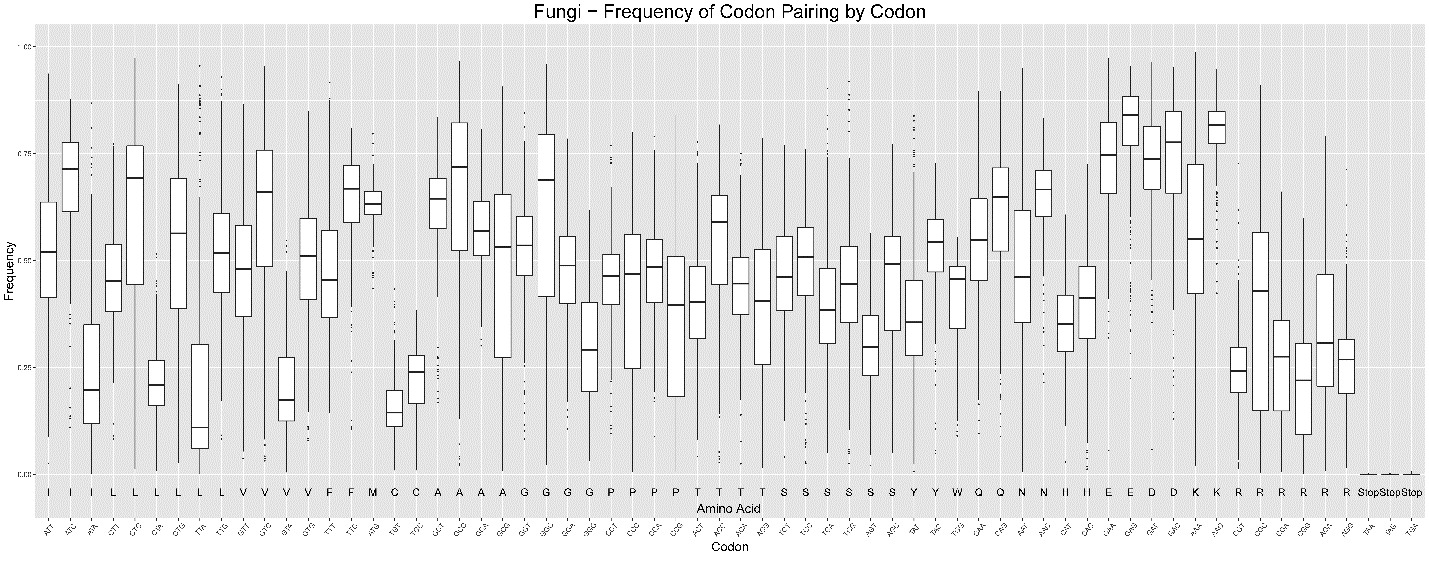


### S14 Figure: Invertebrates


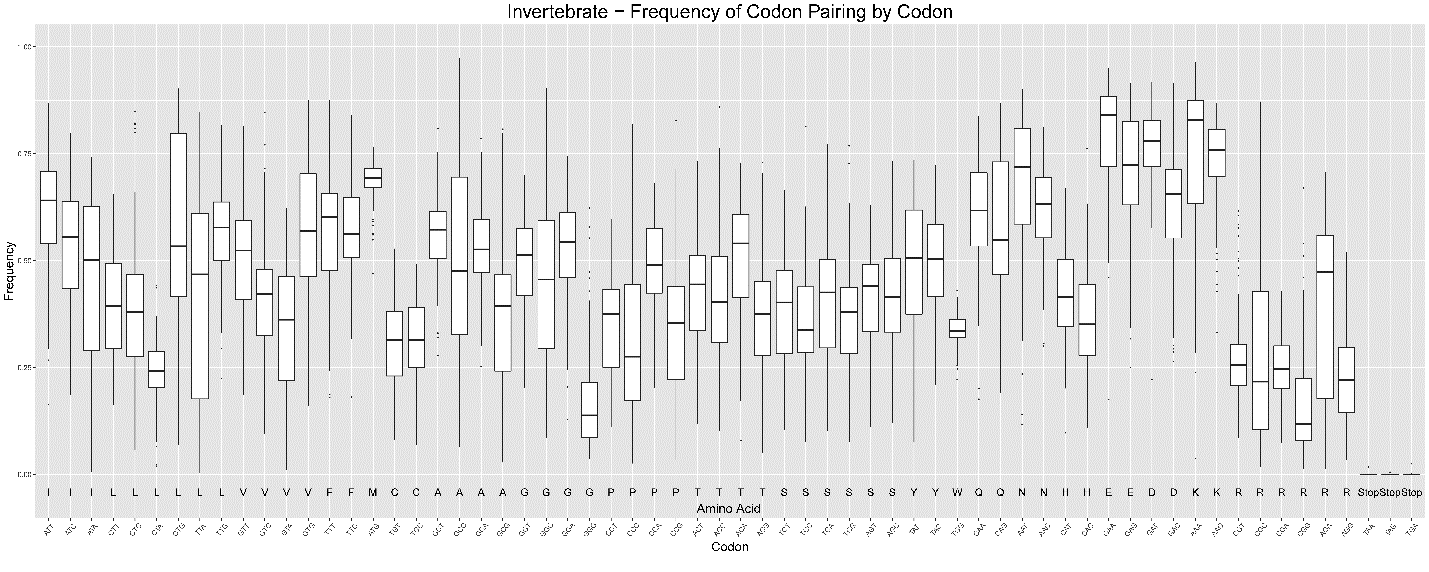


### S15 Figure: Mammals


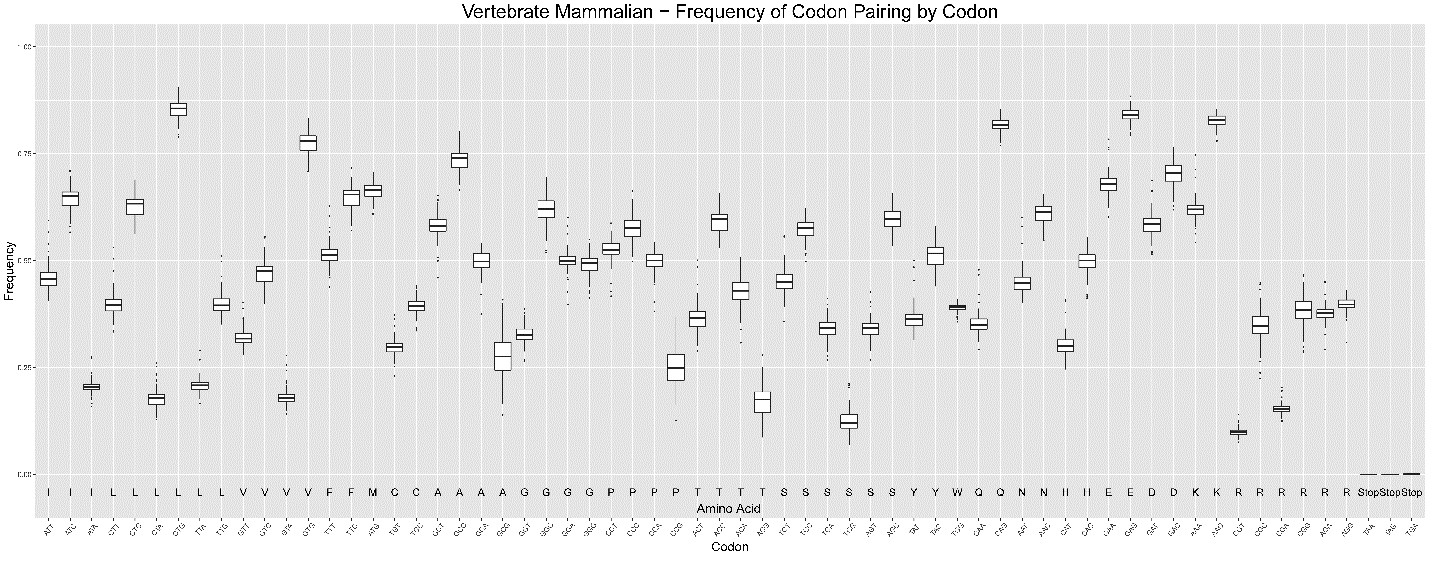


### S16 Figure: Other Vertebrates


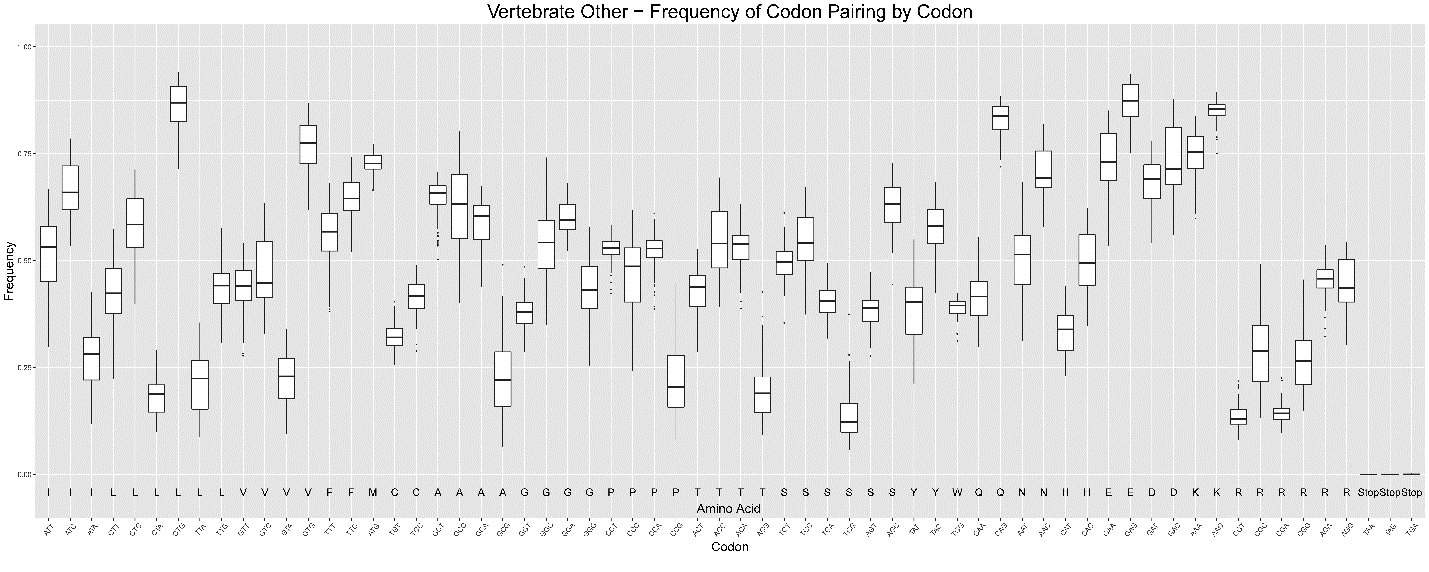


### S17 Figure: Plants


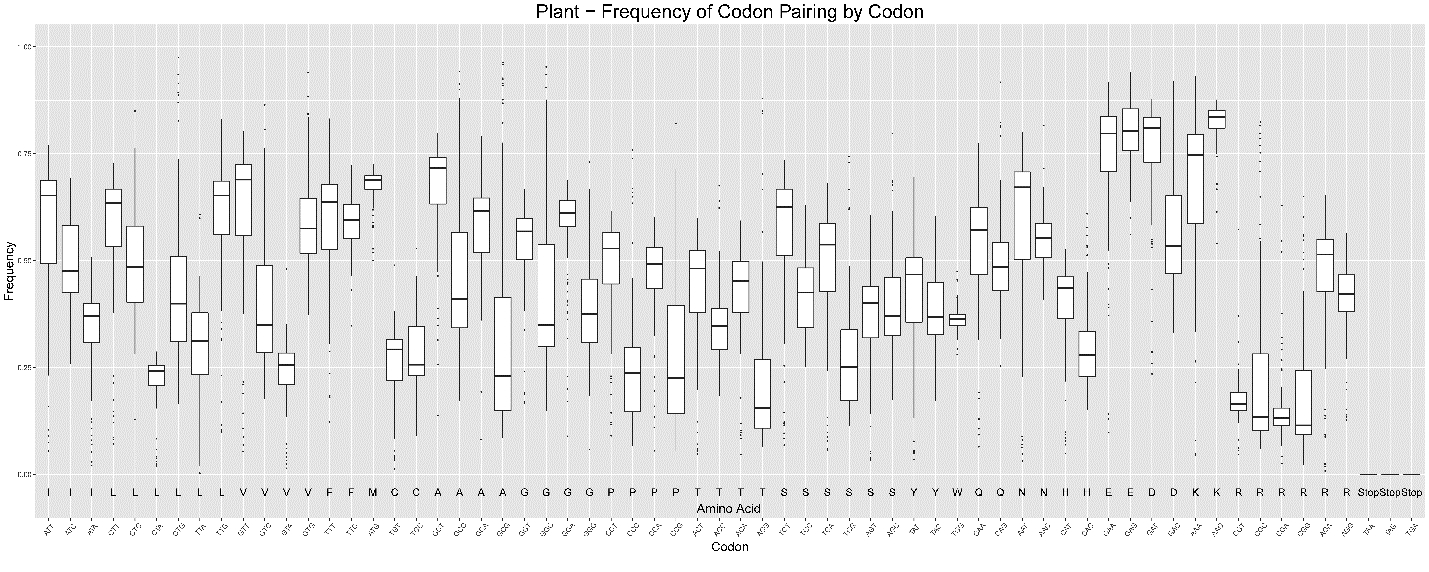


### S18 Figure: Protozoa


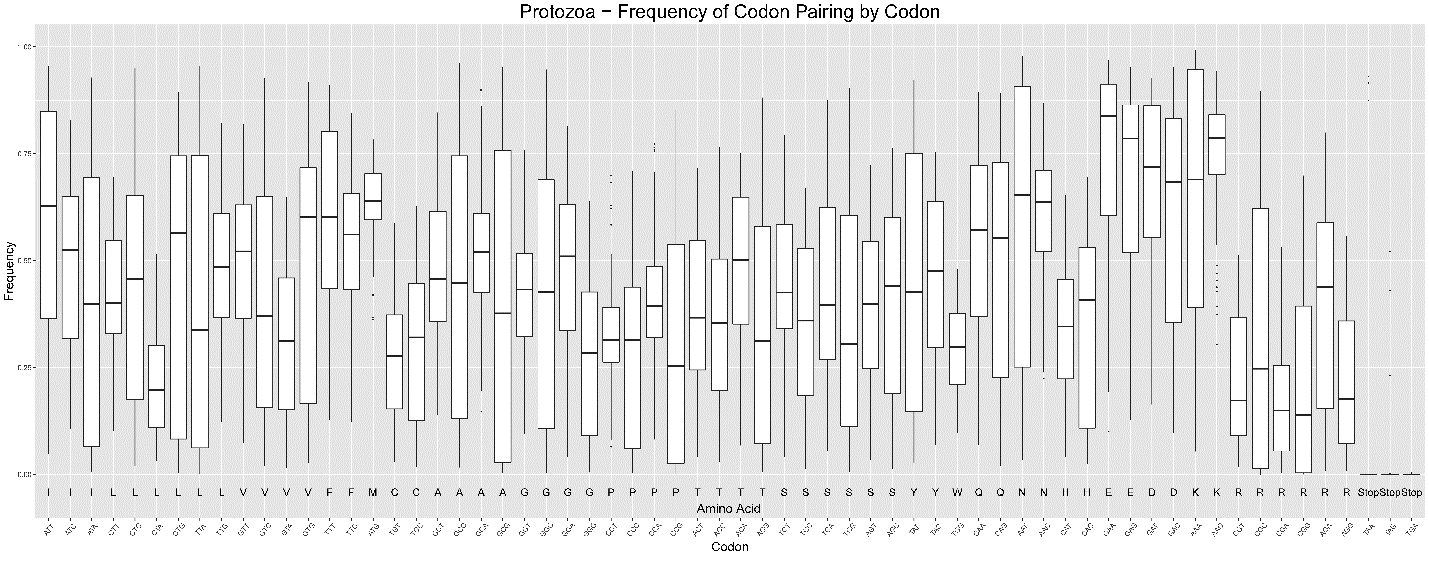


### S19 Figure: Viruses


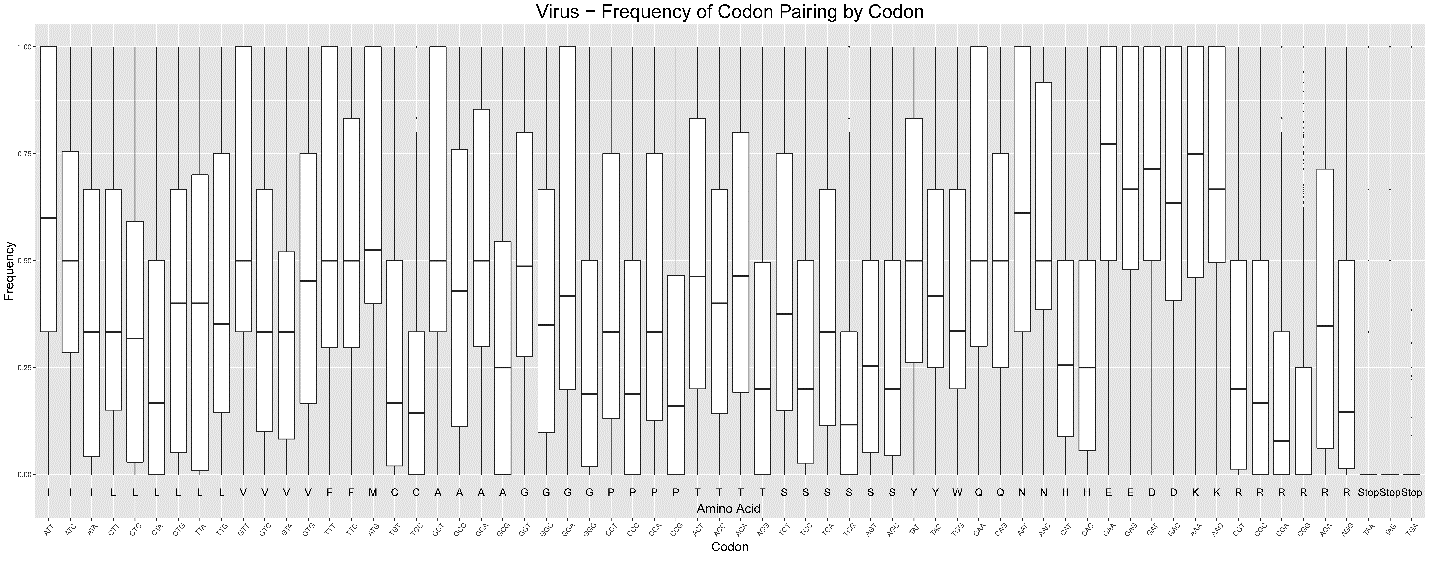


## Number of Codons Included in Each Motif

### S20 Figure: All Species


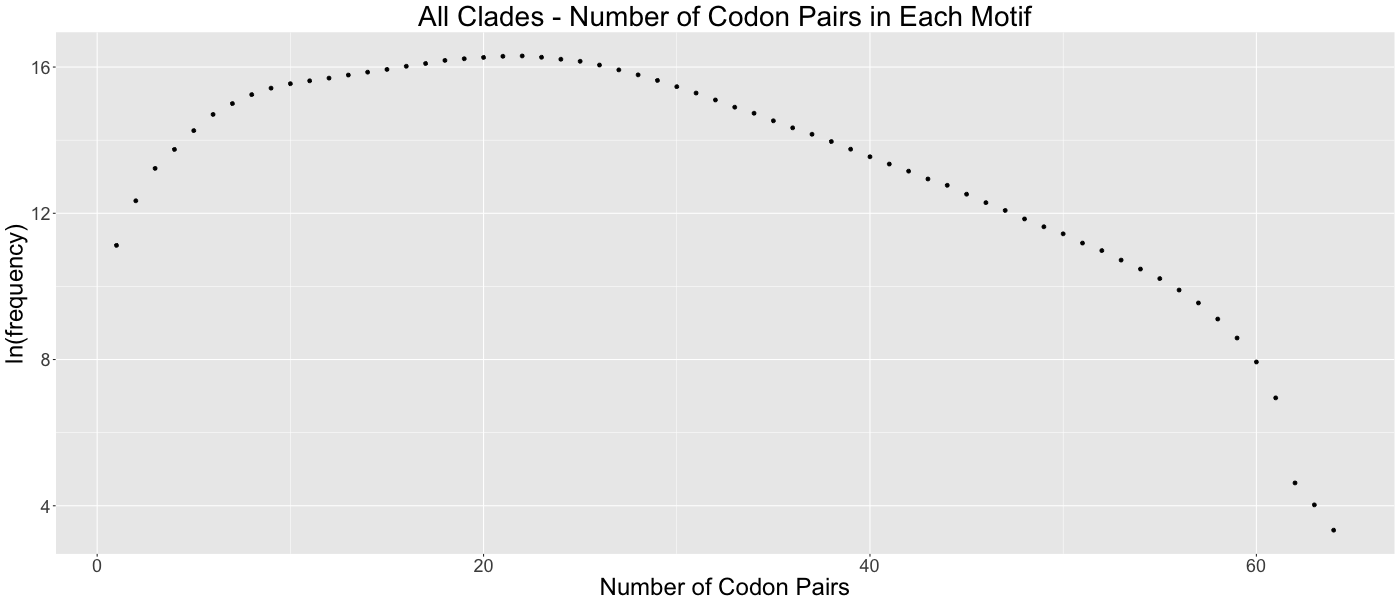


### S21 Figure: Archaea


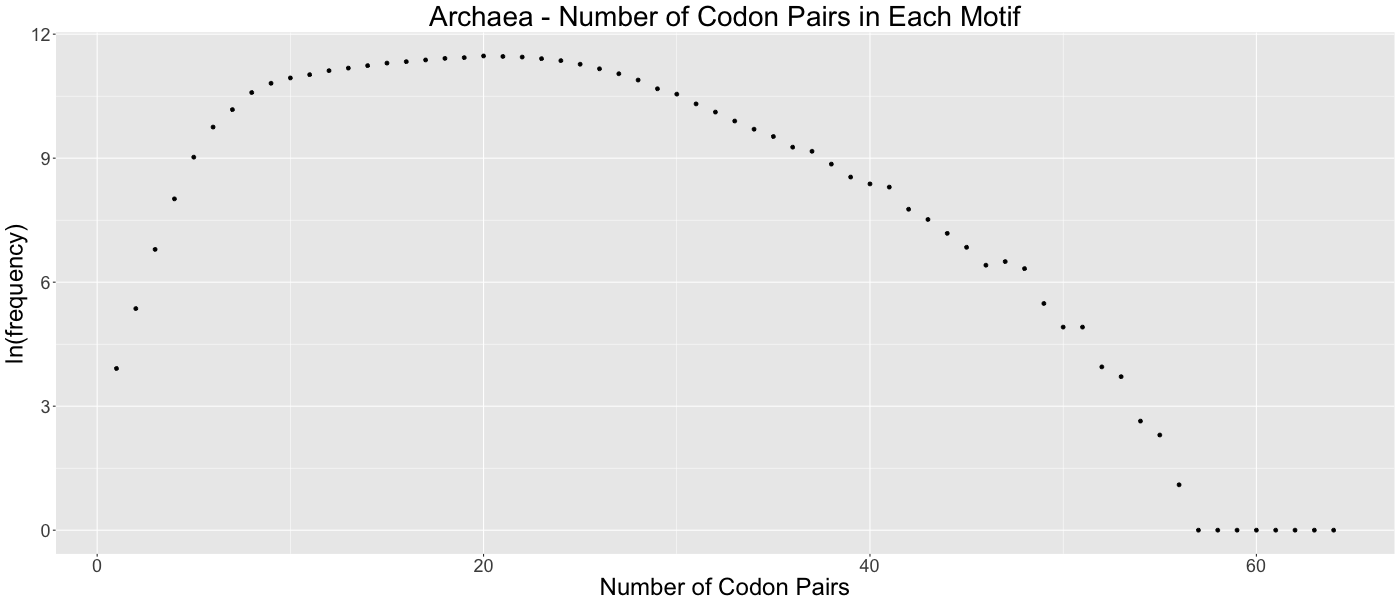


### S22 Figure: Bacteria


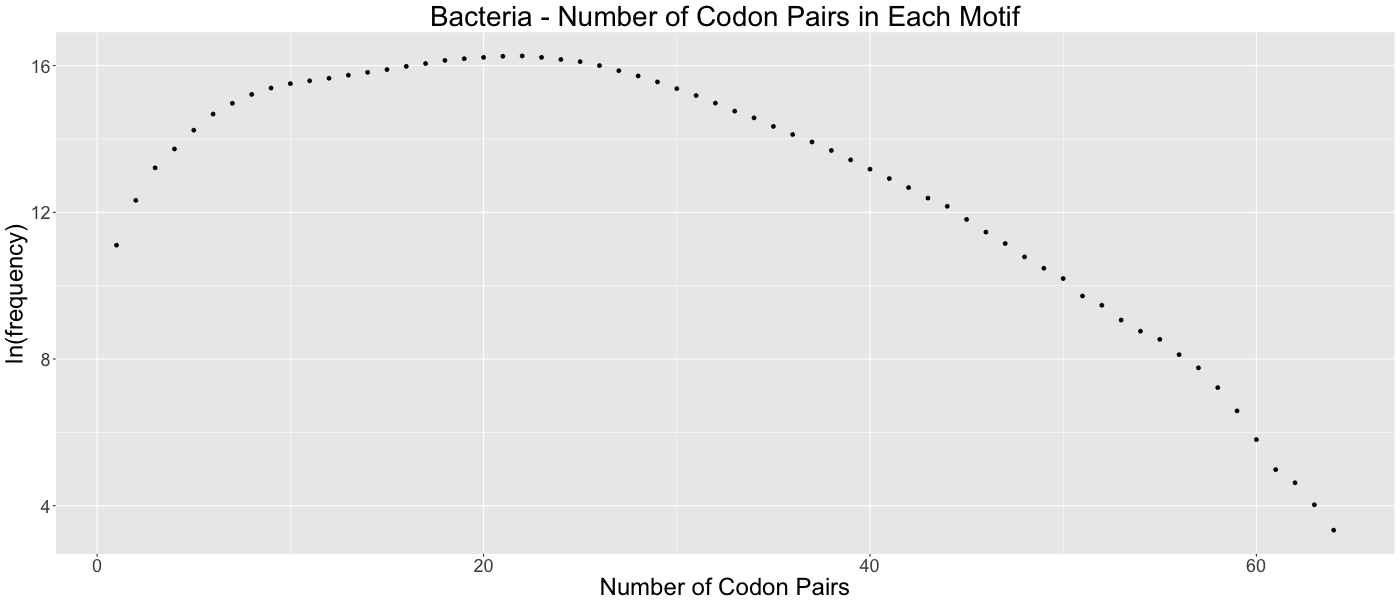


### S23 Figure: Fungi


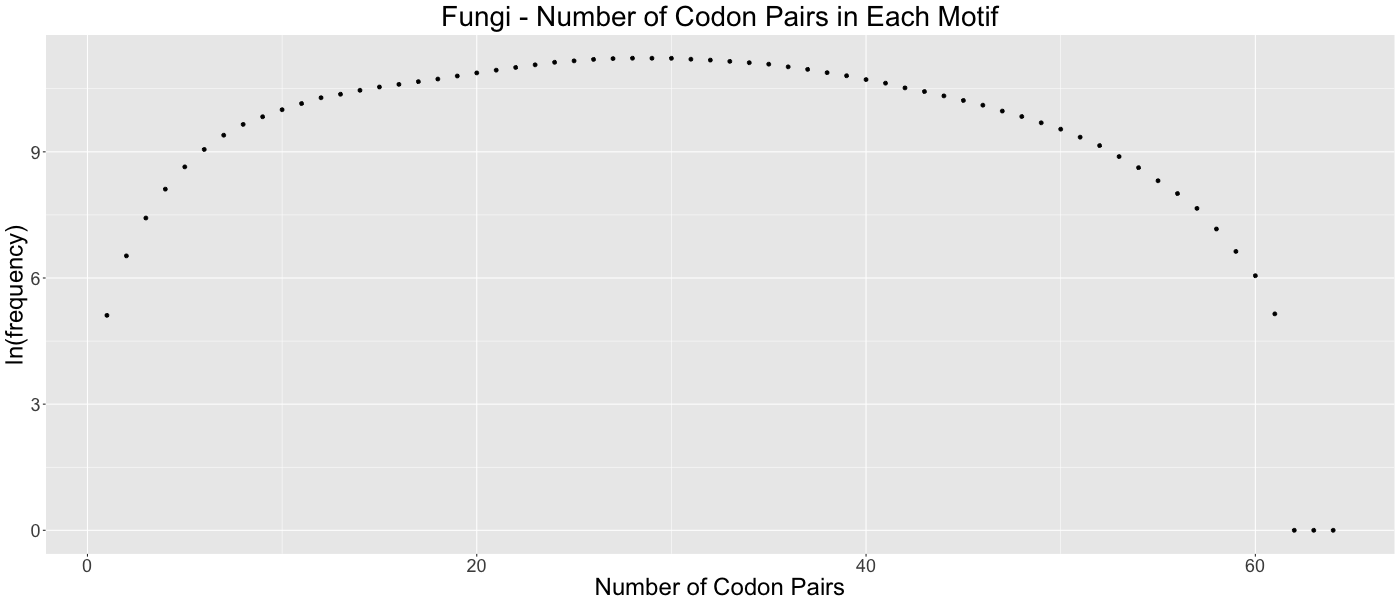


### S24 Figure: Invertebrates


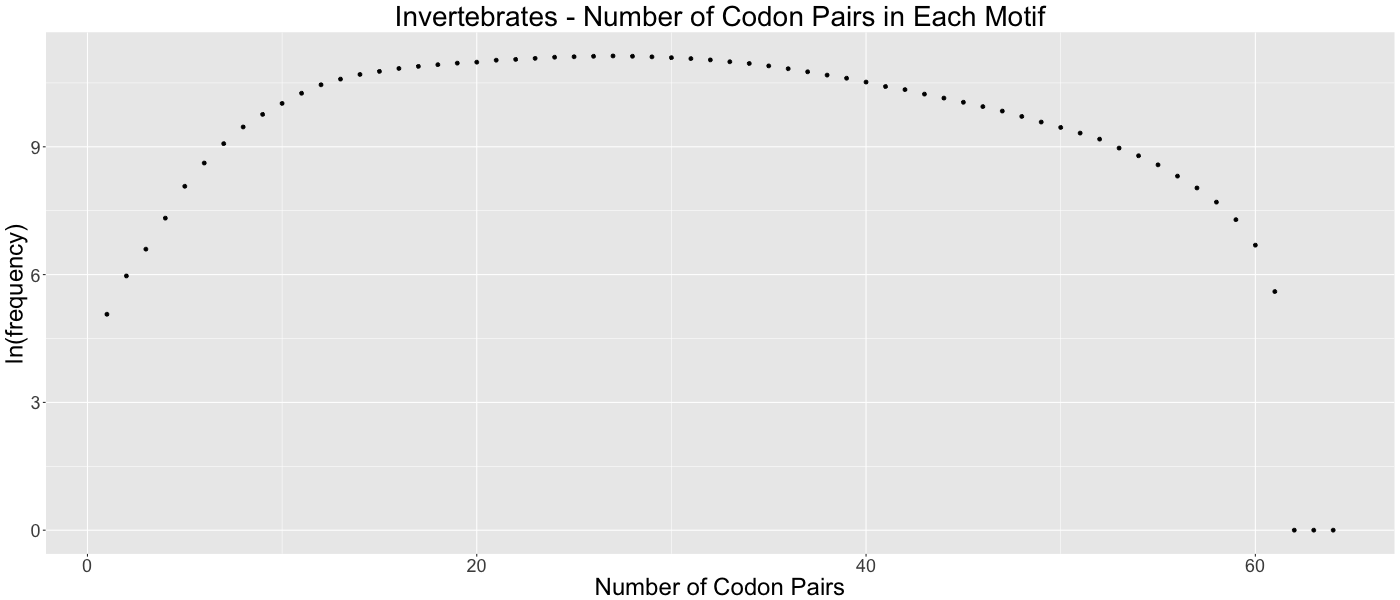


### S25 Figure: Mammals


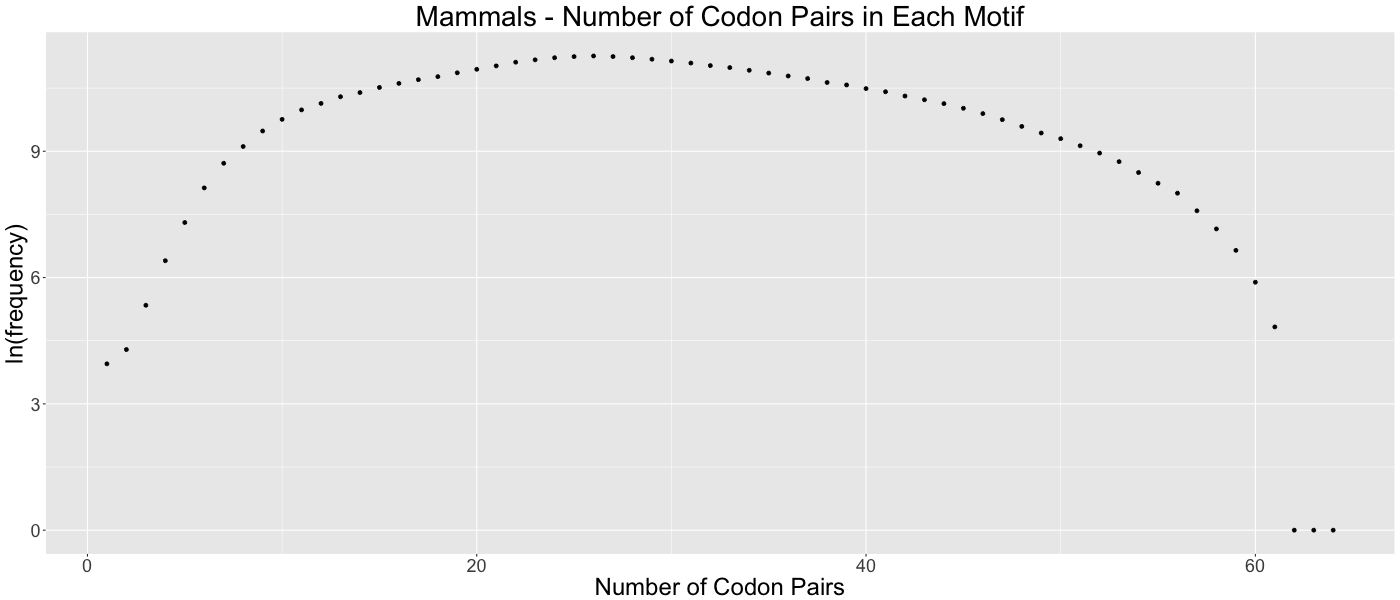


### S26 Figure: Other Vertebrates


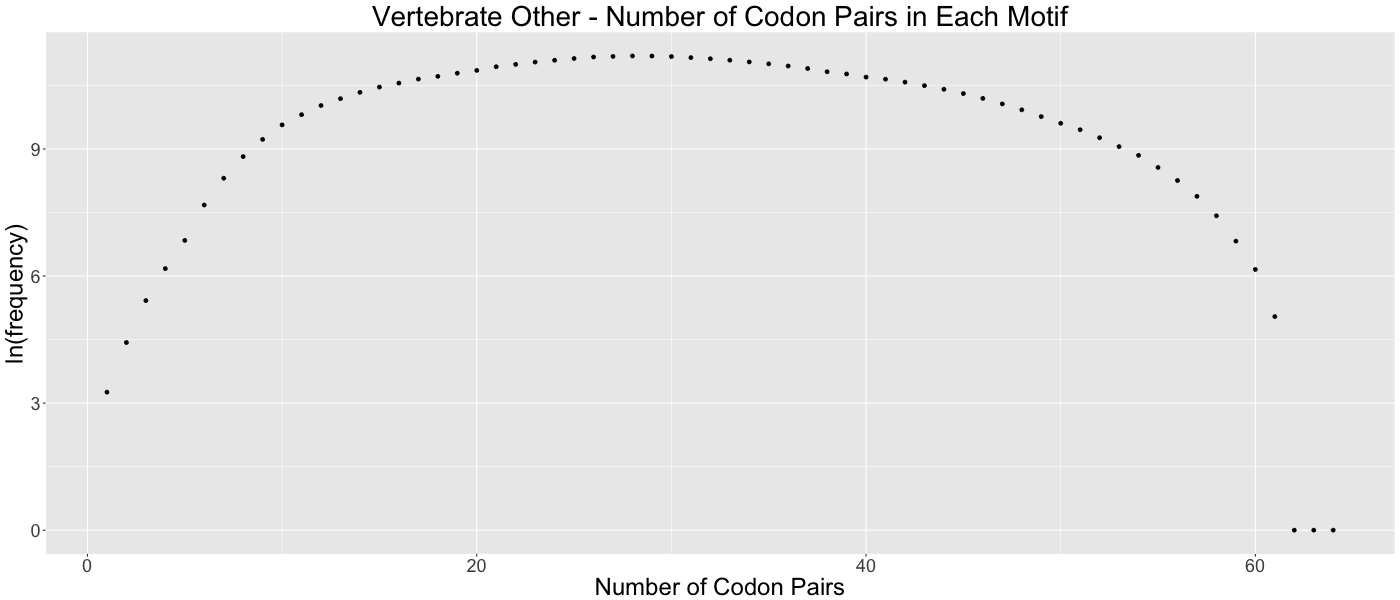


### S27 Figure: Plants


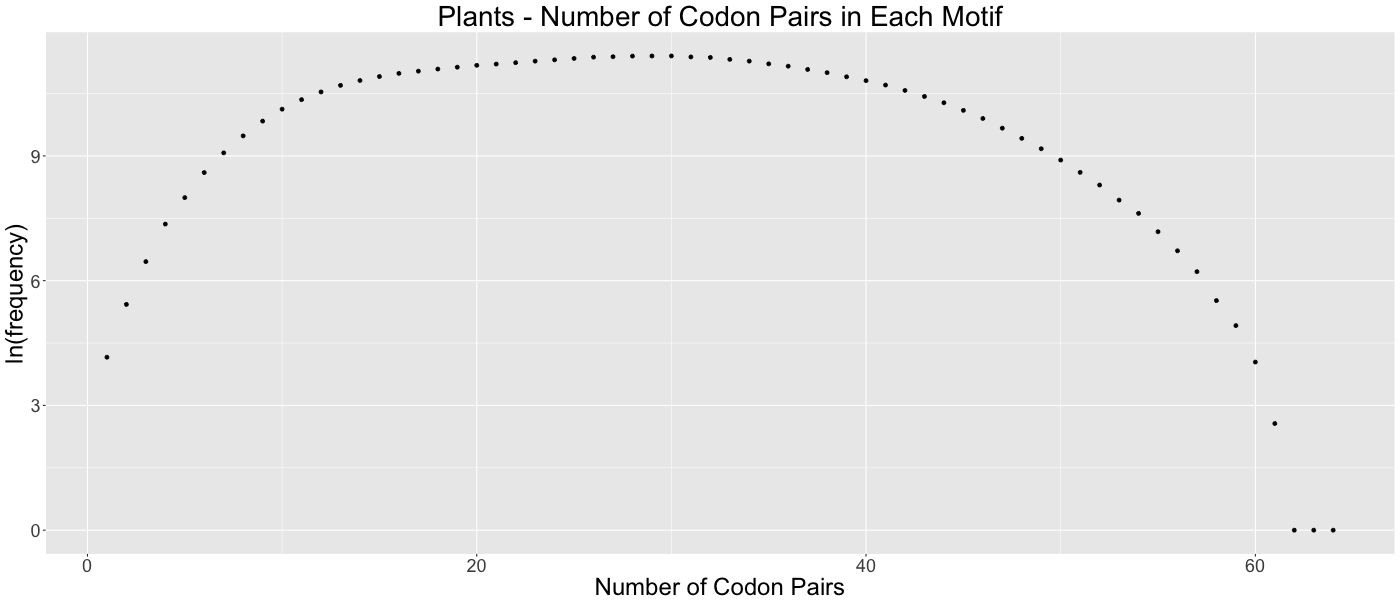


### S28 Figure: Protozoa


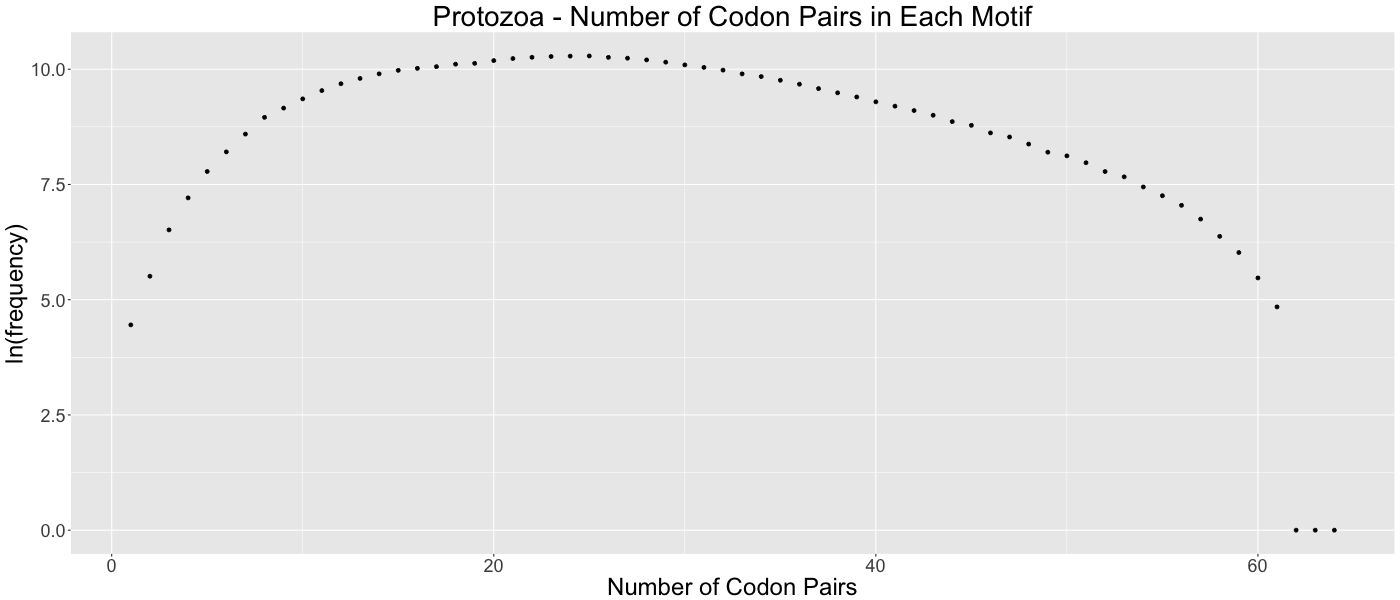


### S29 Figure: Viruses


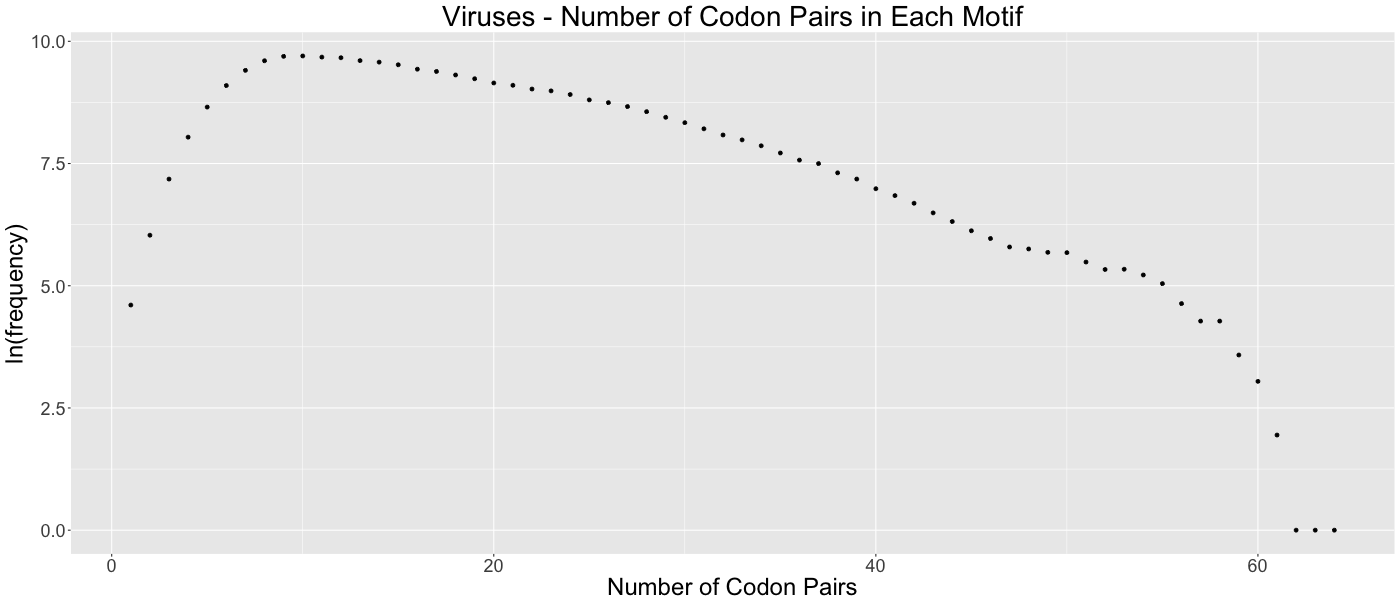


## Number of Times Motifs are Repeated

### S30 Figure: All Species


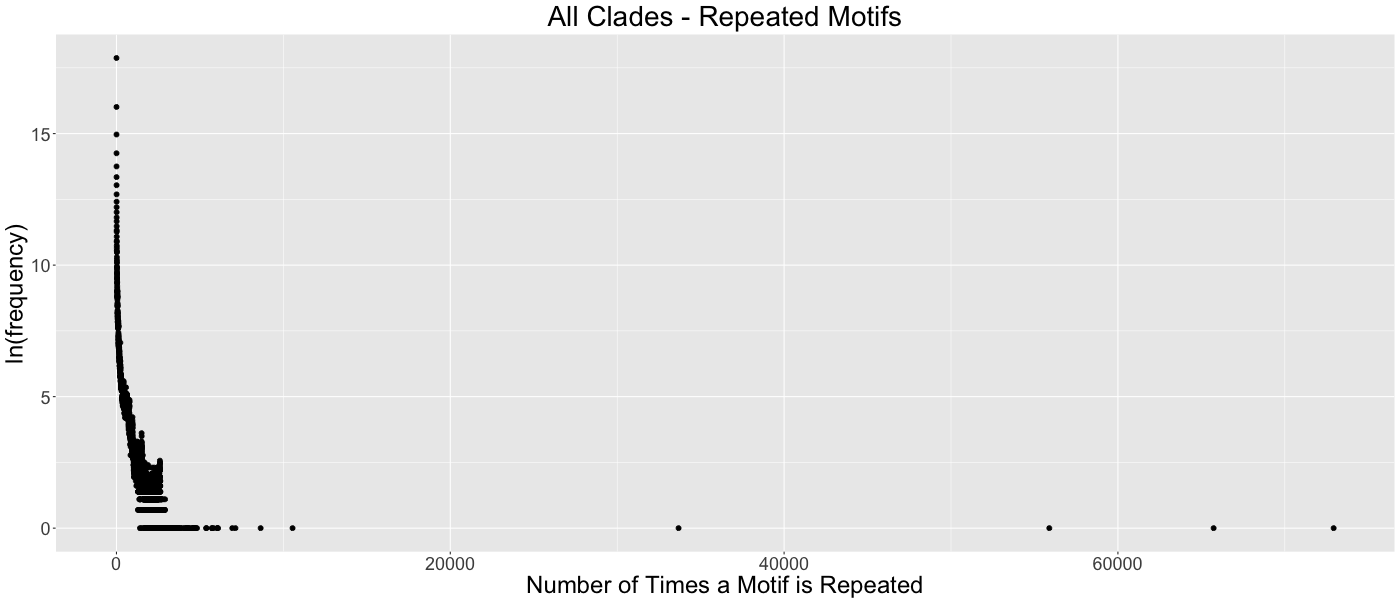


### S31 Figure: Archaea


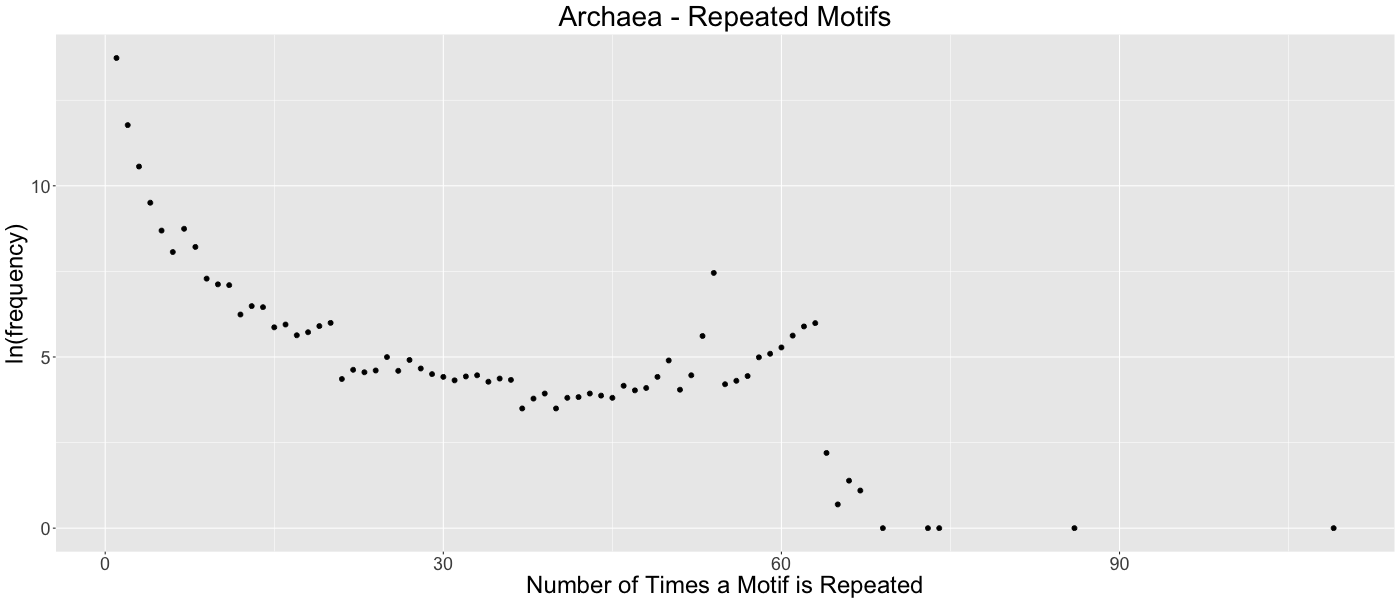


### S32 Figure: Bacteria


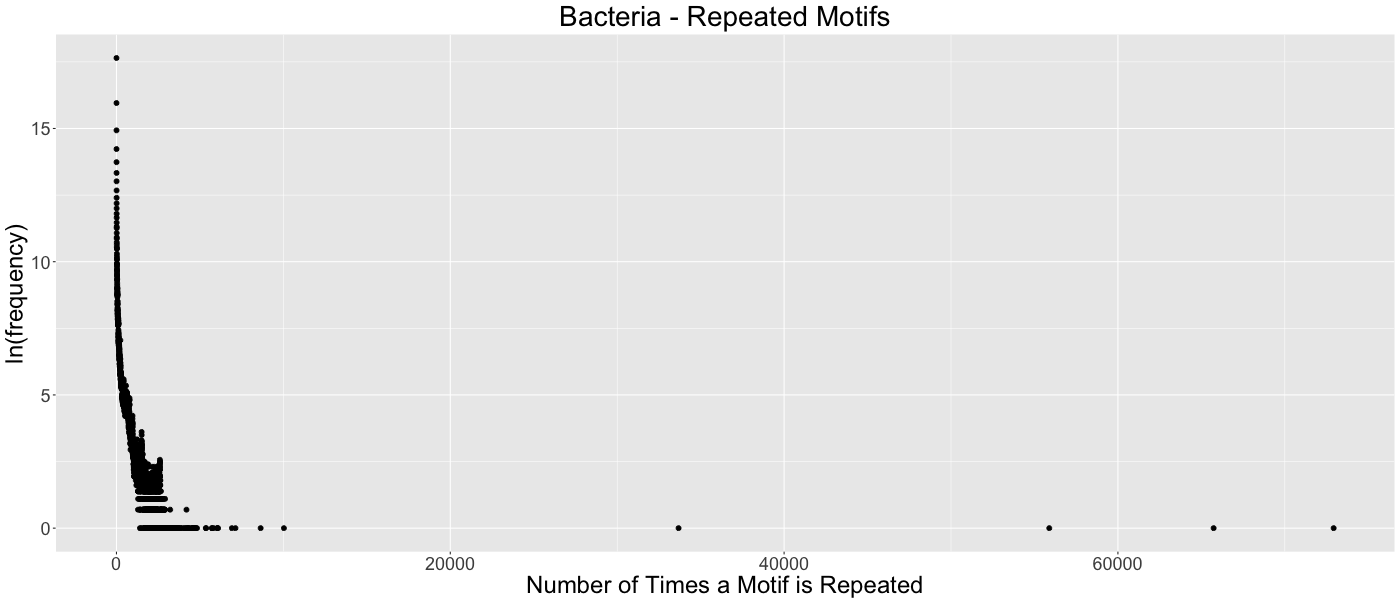


### S33 Figure: Fungi


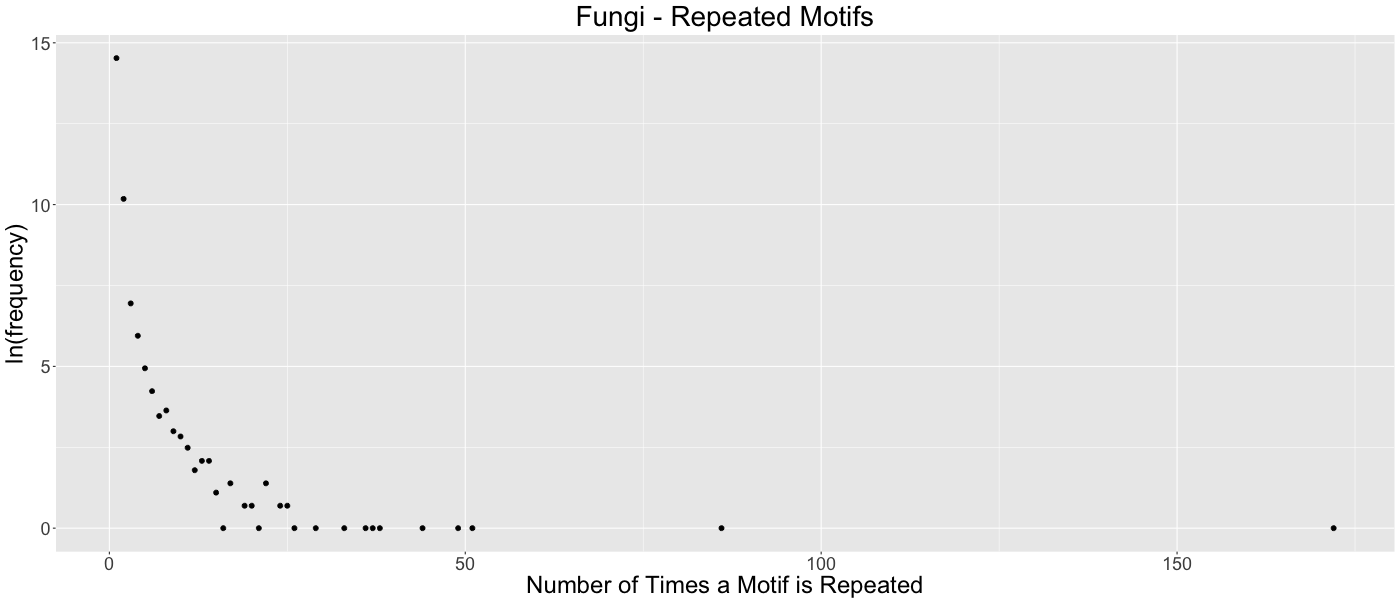


### S34 Figure: Invertebrates


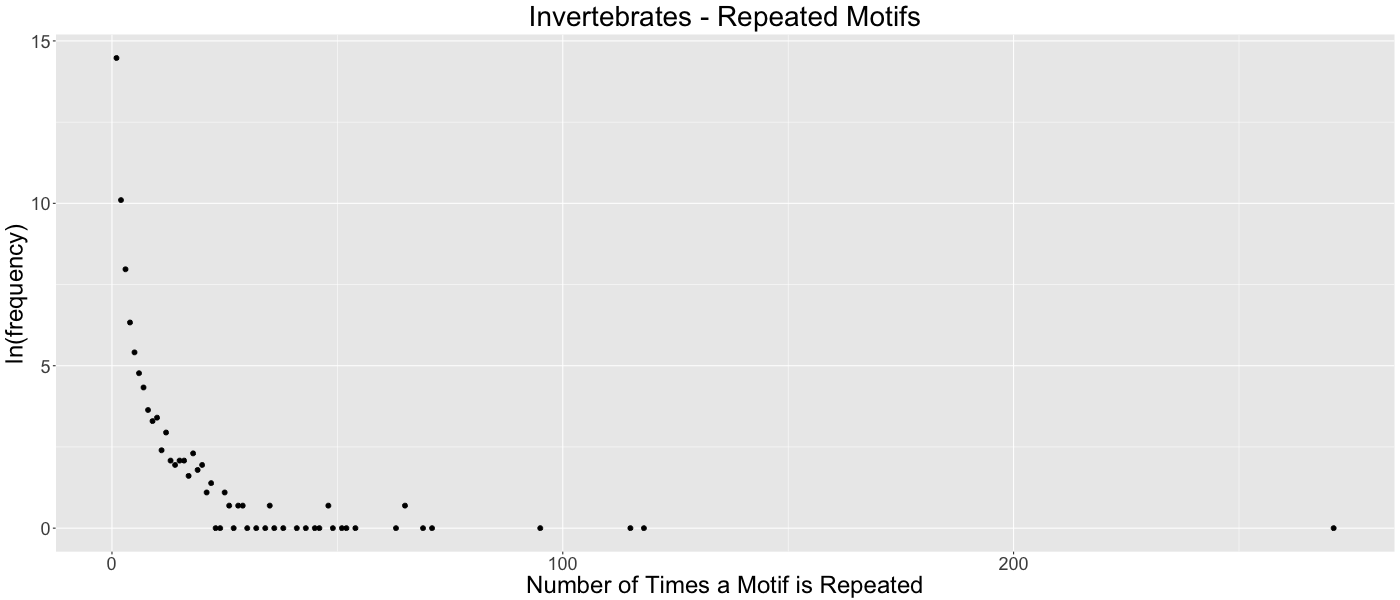


### S35 Figure: Mammals


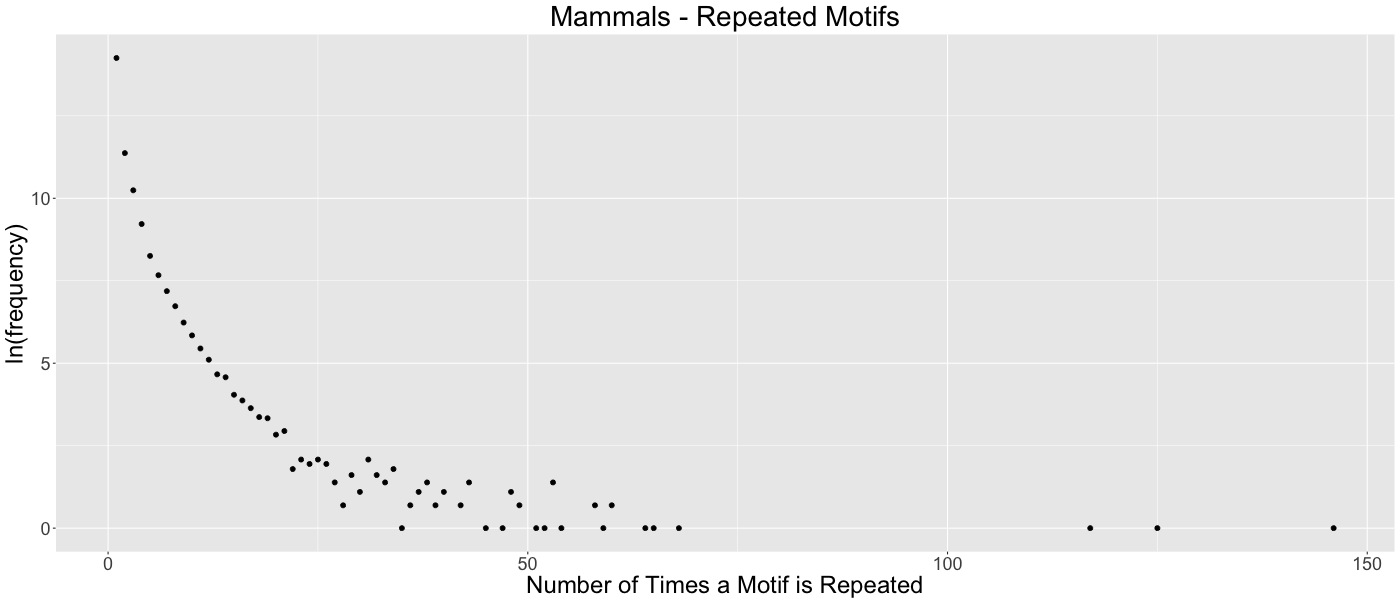


### S36 Figure: Other Vertebrates


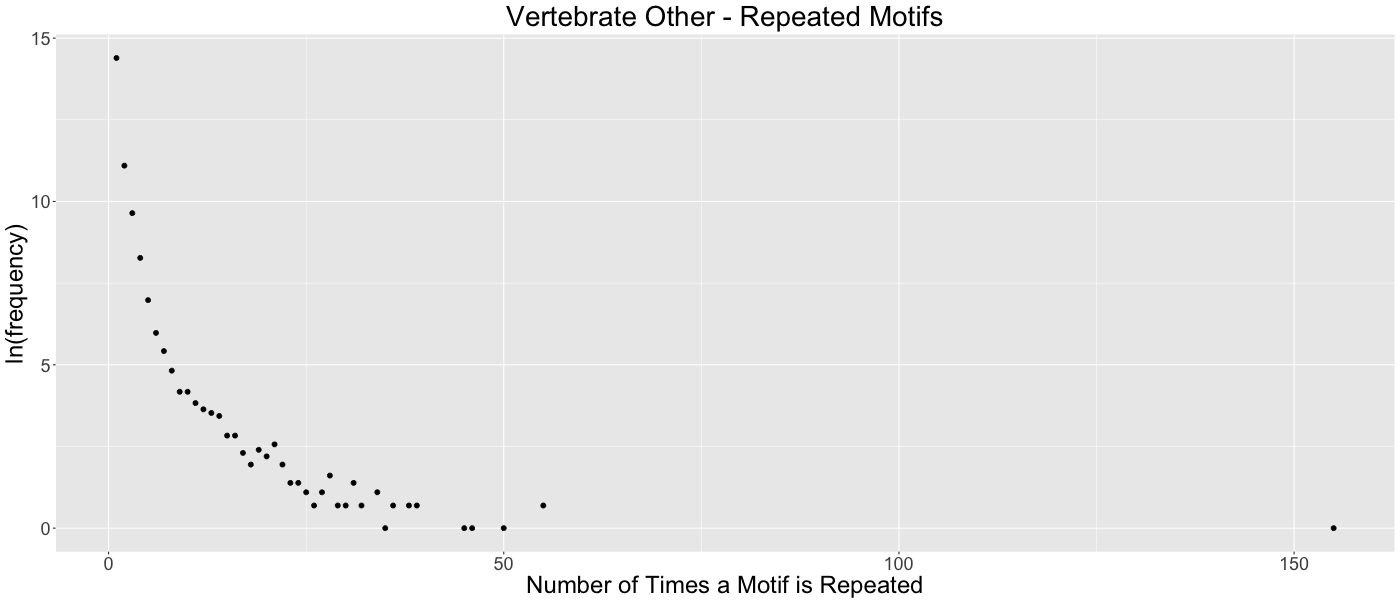


### S37 Figure: Plants


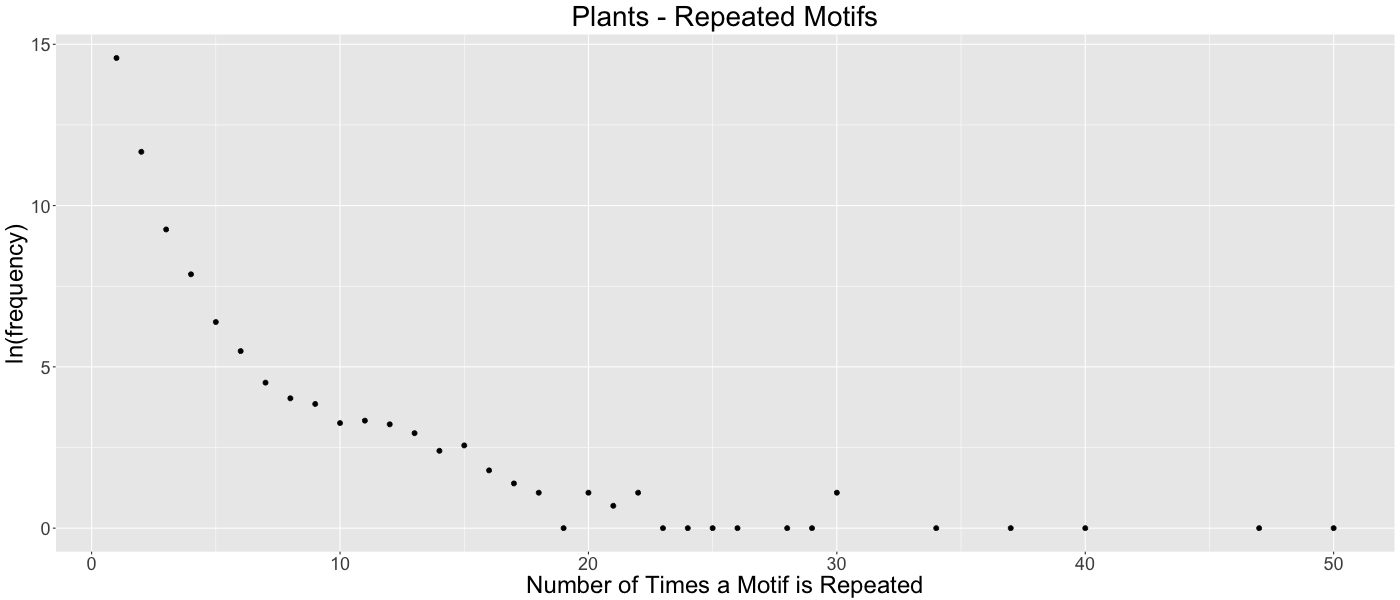


### S38 Figure: Protozoa


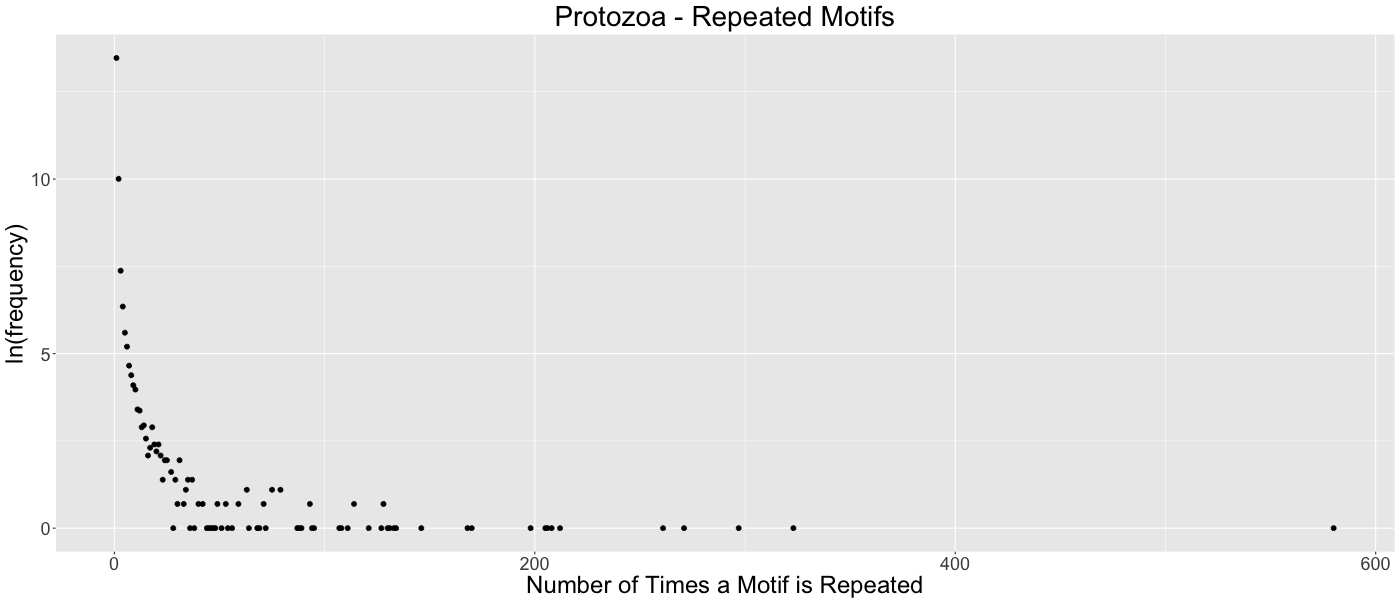


### S39 Figure: Viruses


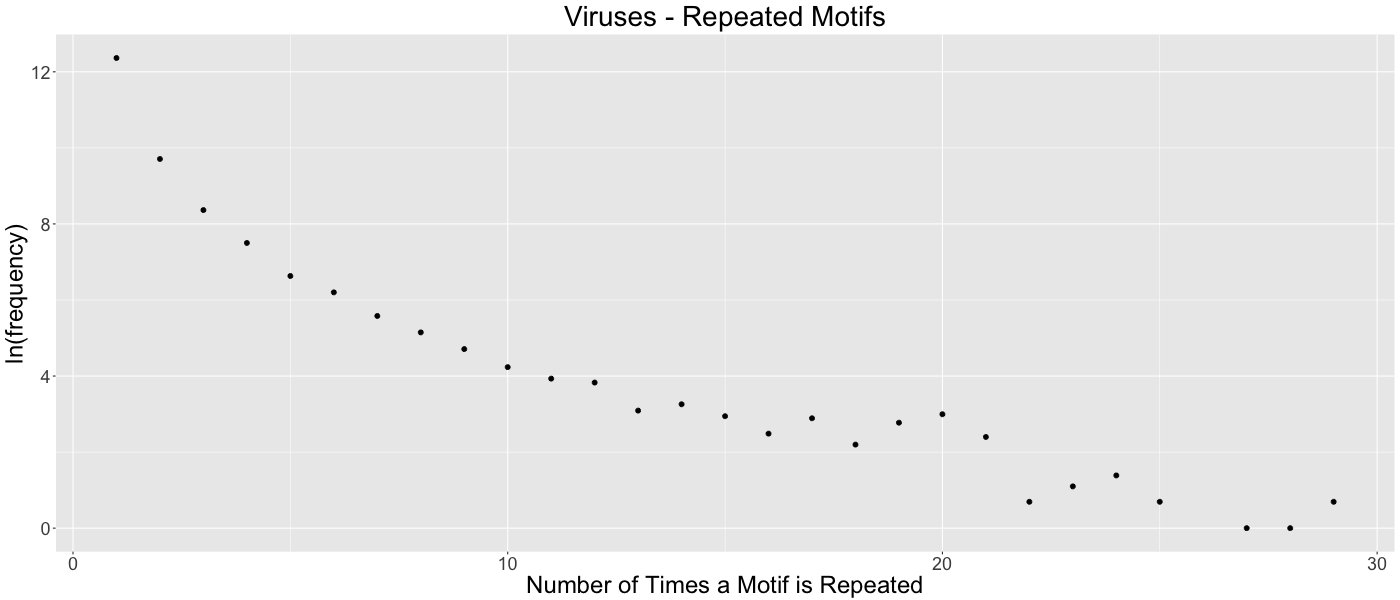


## Number of Codon Pairs versus Gene Length

### S40 Figure: Archaea


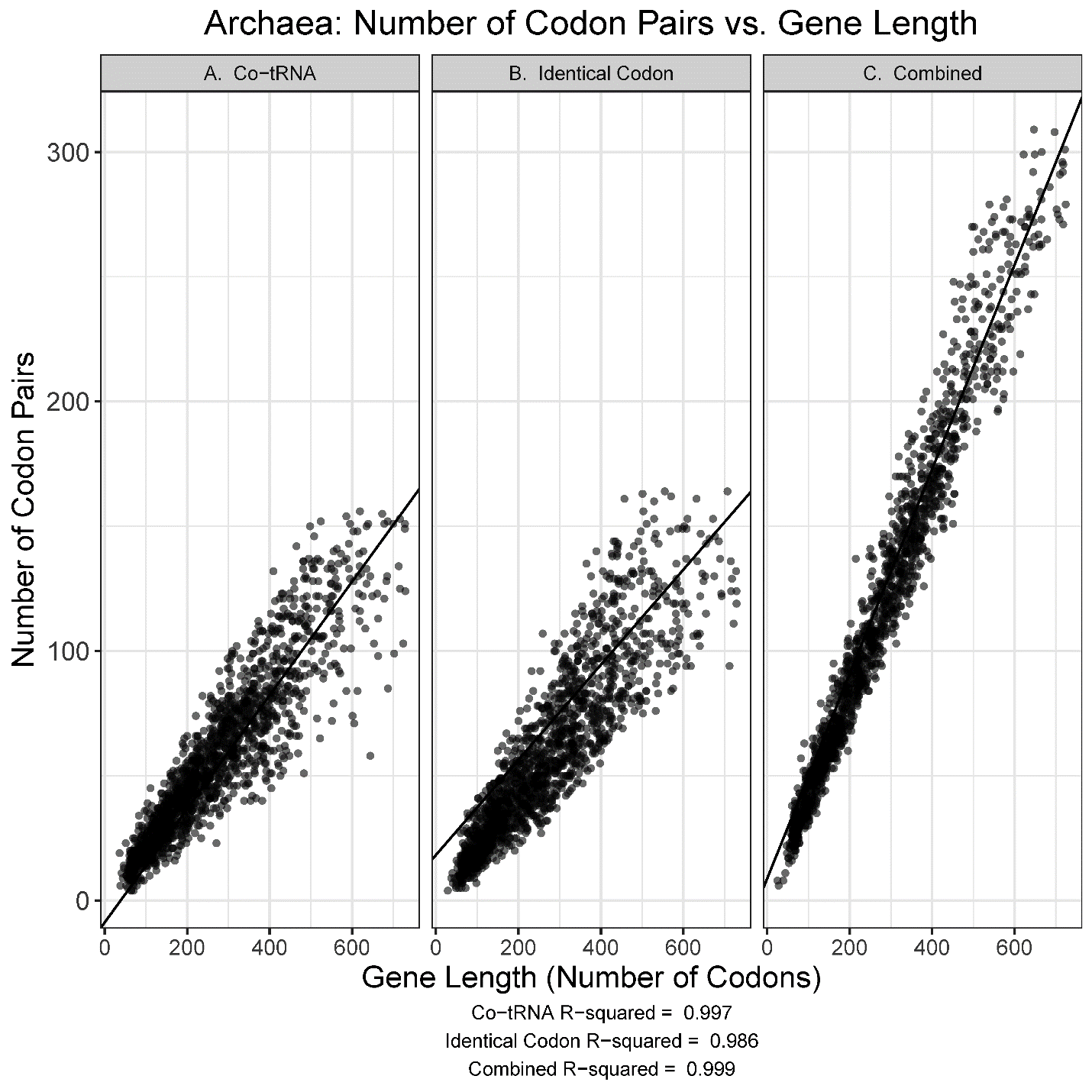


### S41 Figure: Bacteria


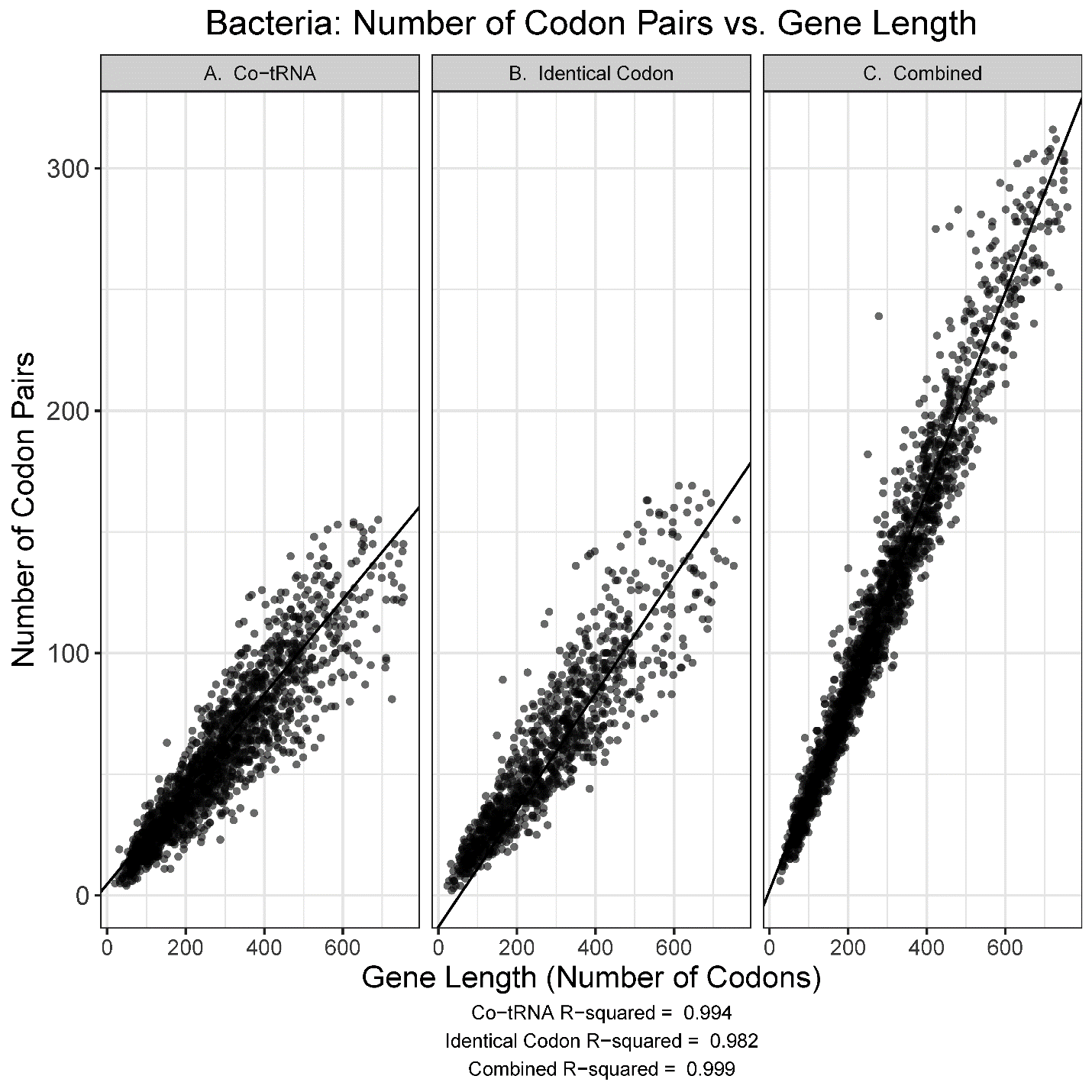


### S42 Figure: Fungi


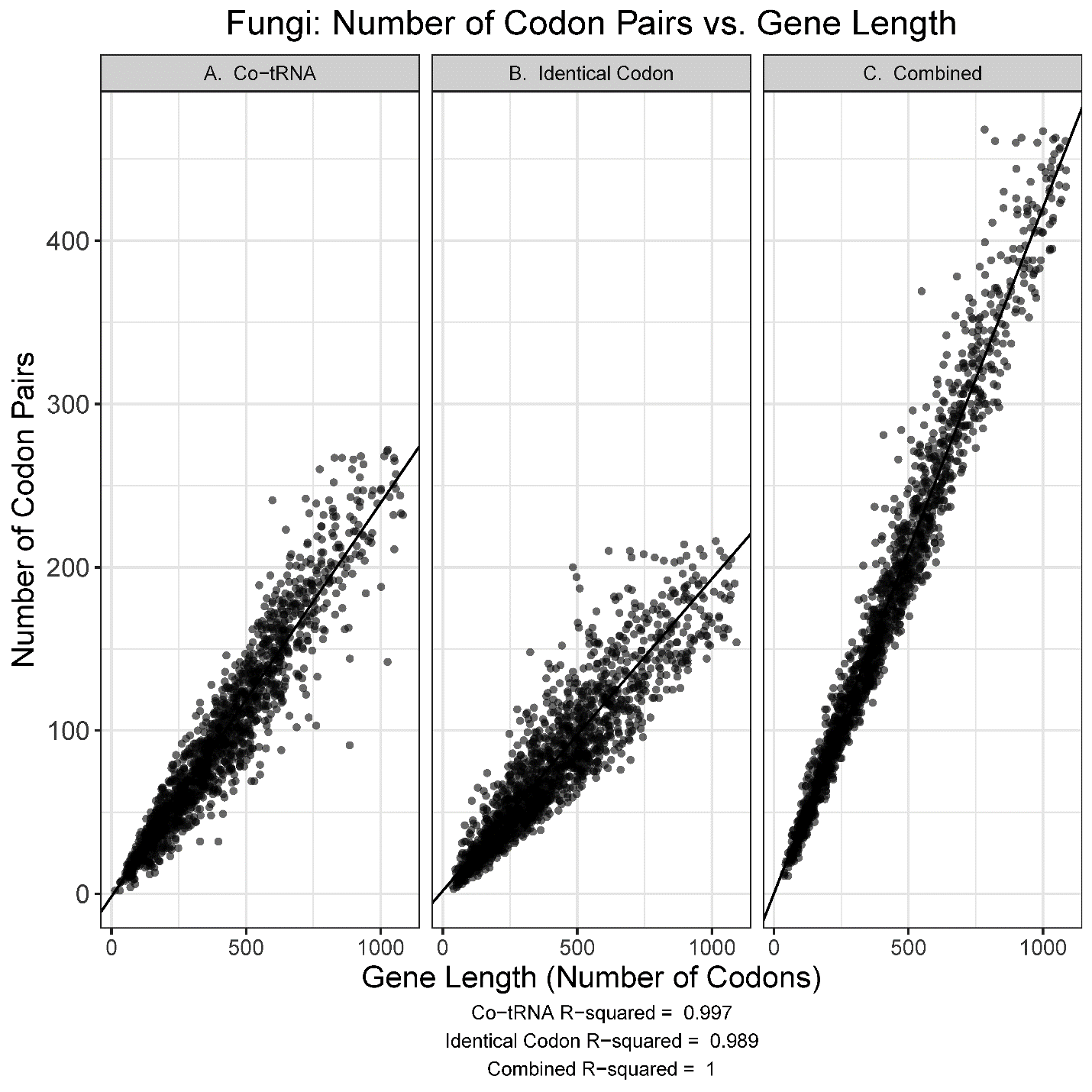


### S43 Figure: Invertebrates


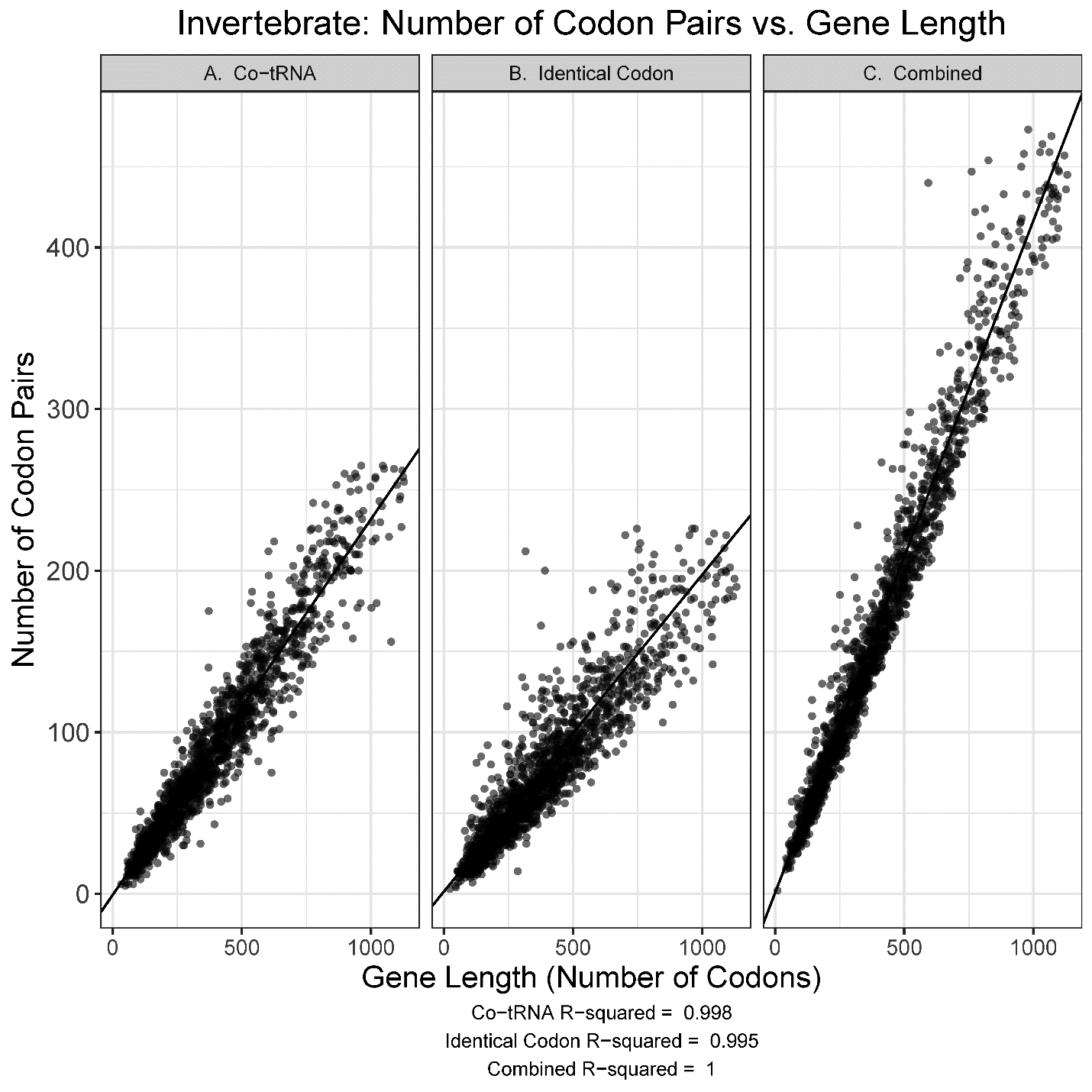


### S44 Figure: Mammals


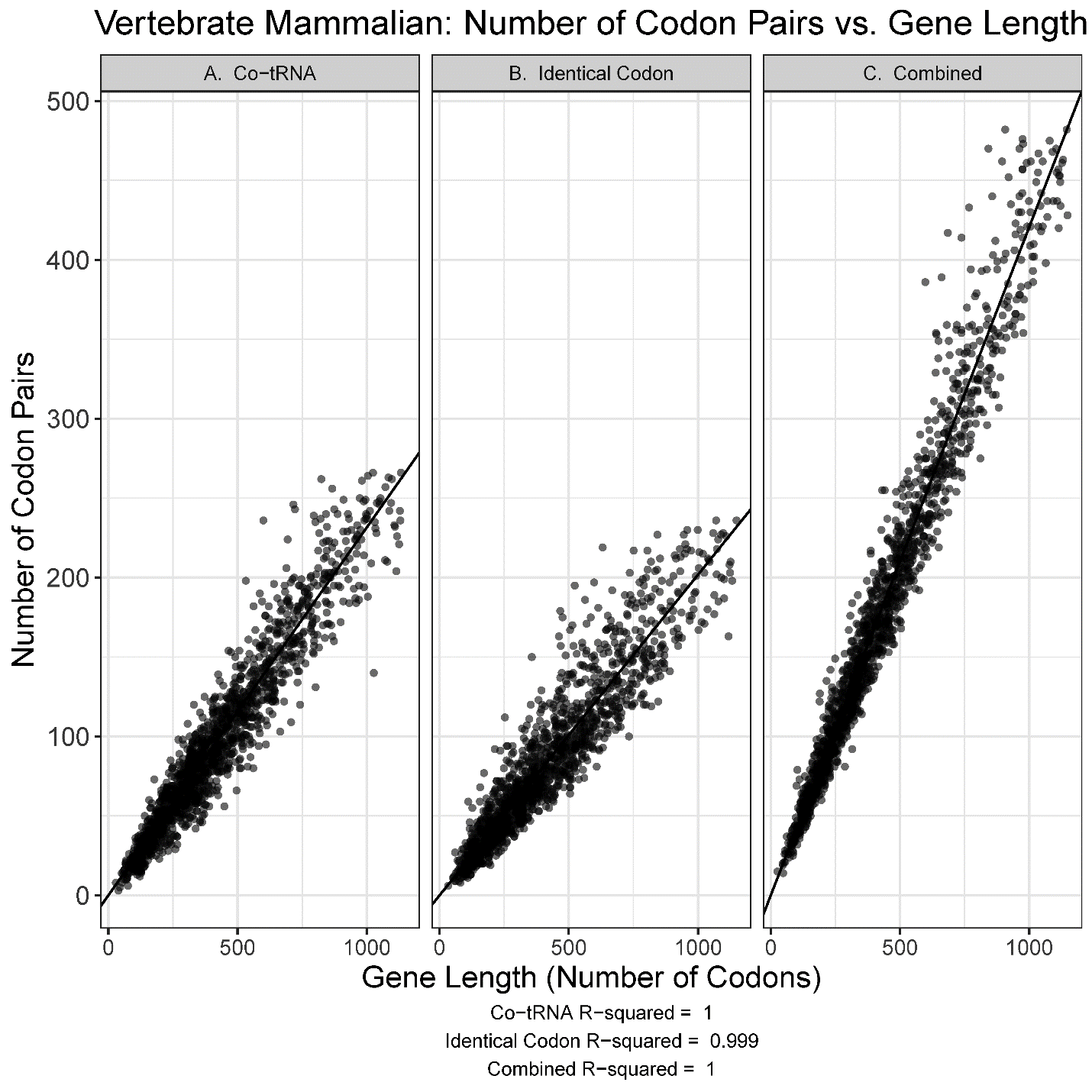


### S45 Figure: Other Vertebrates


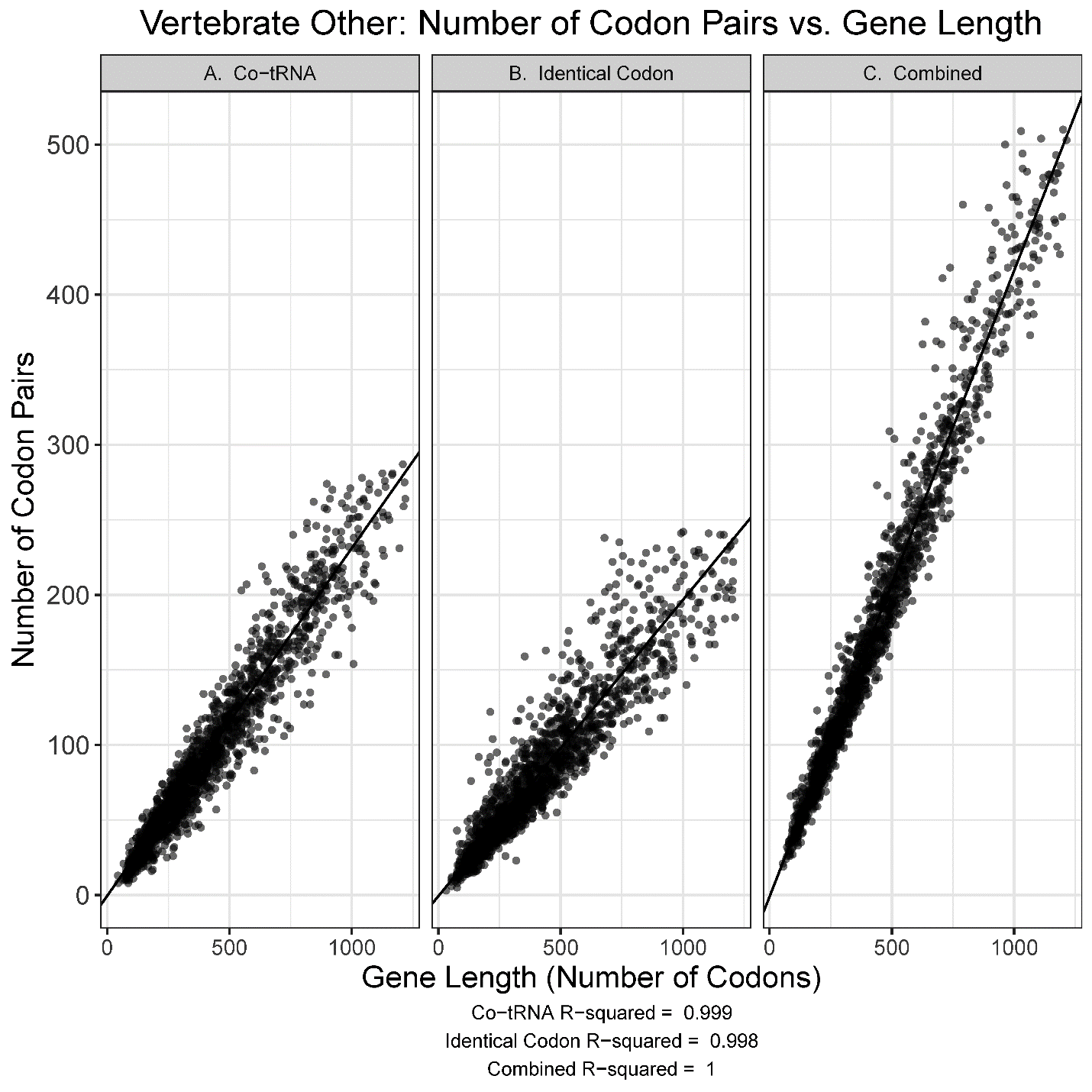


### S46 Figure: Plants


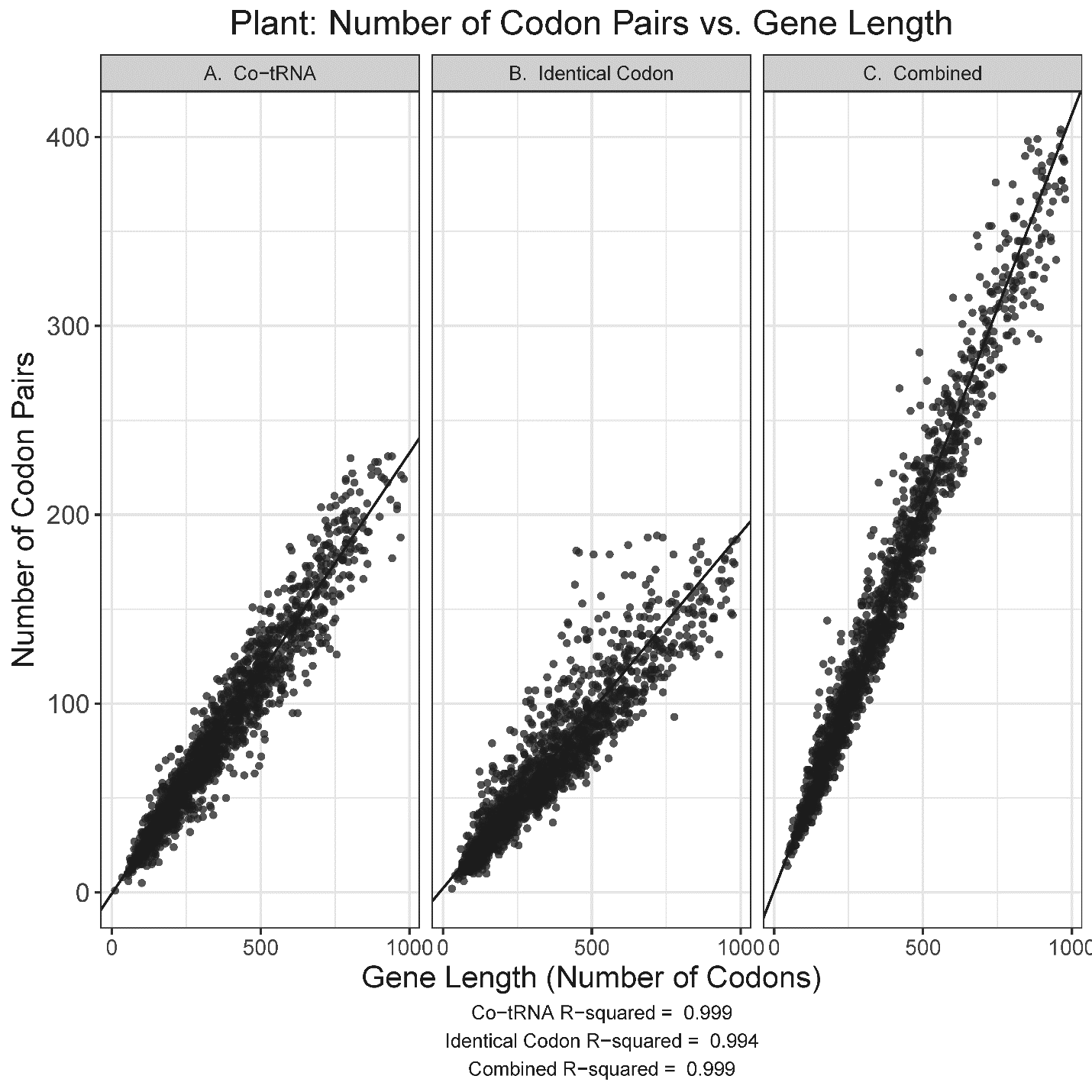


### S47 Figure: Protozoa


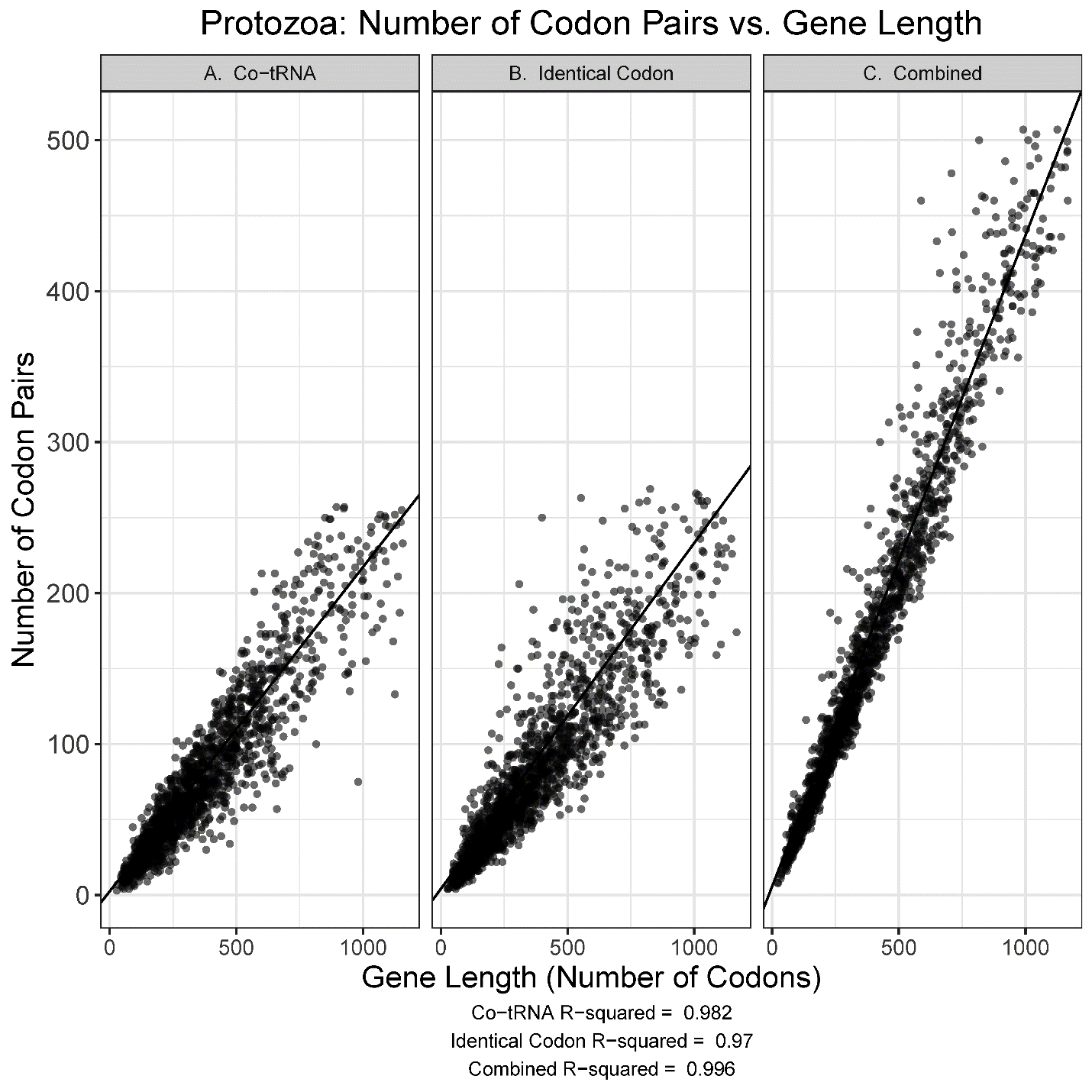


### S48 Figure: Viruses


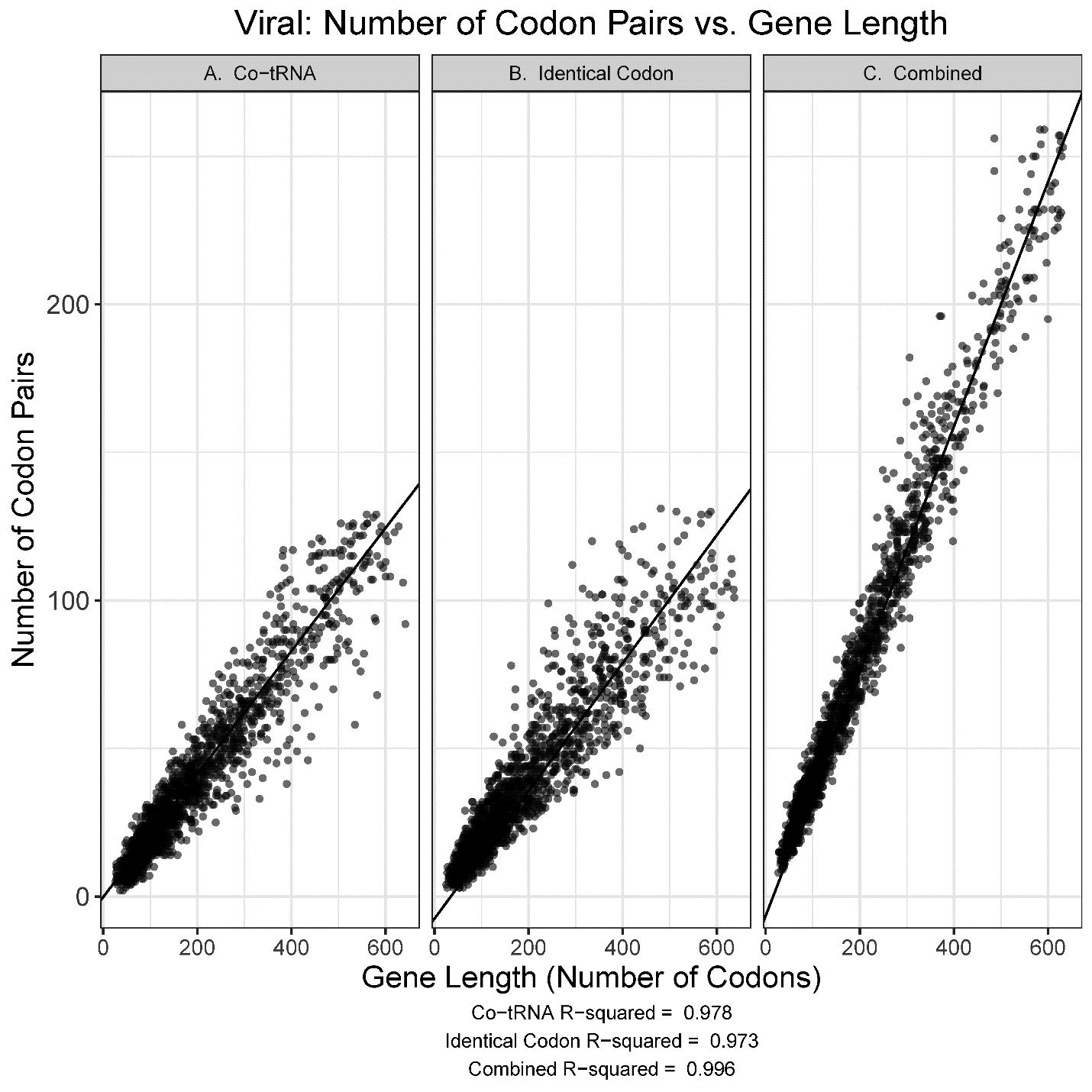


## Number of Codons in Pairing Motif versus Gene Length

### S49 Figure: Archaea


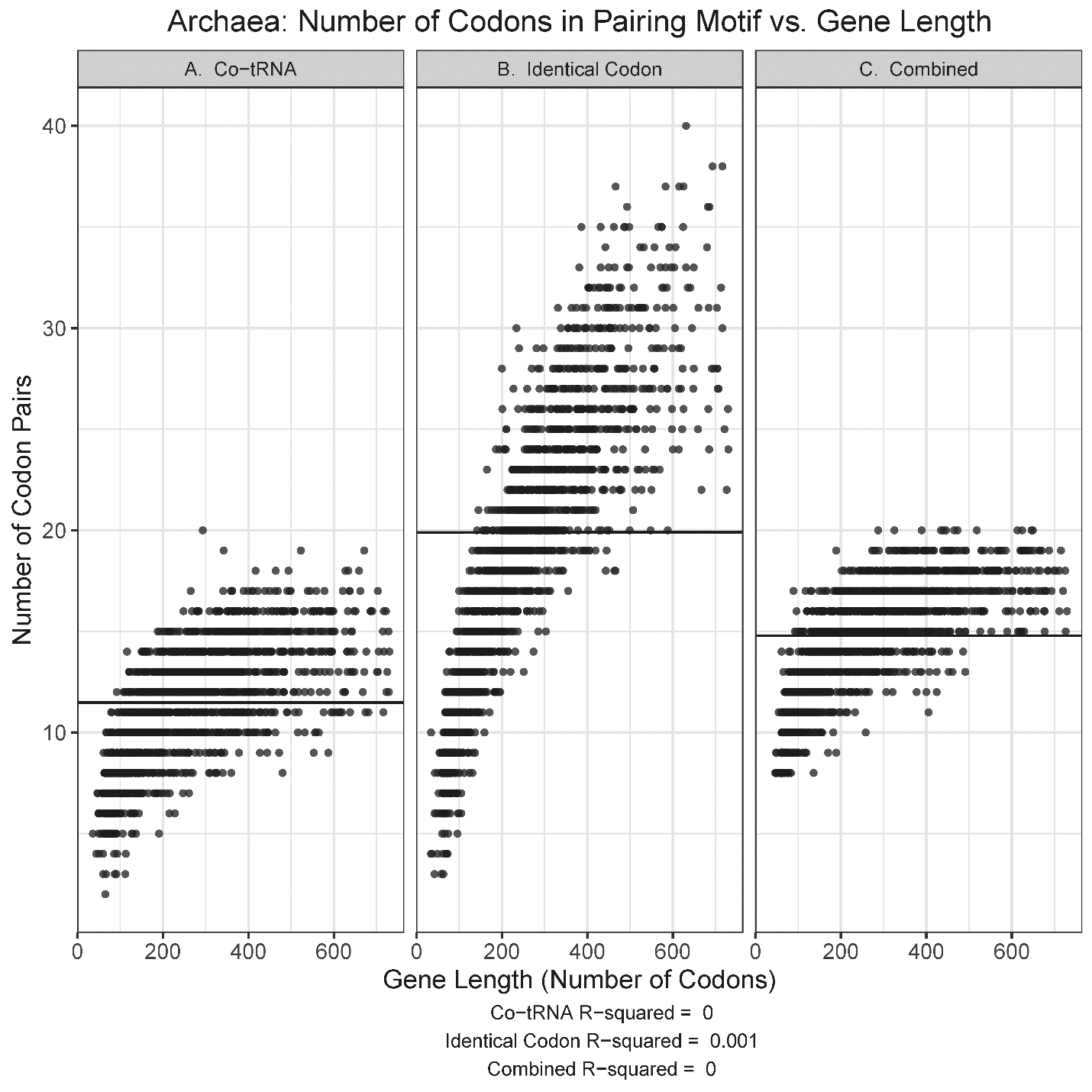


### S50 Figure: Bacteria


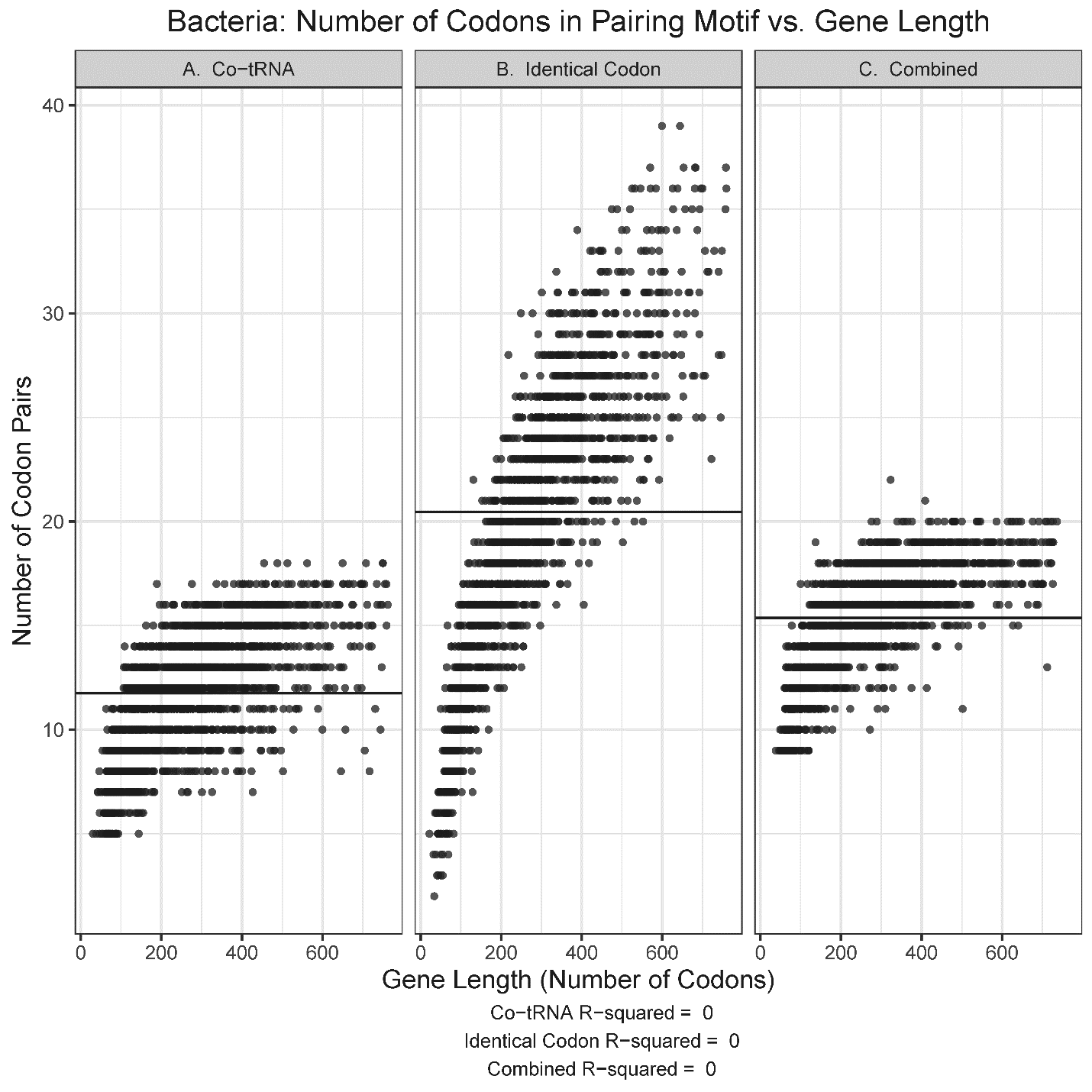


### S51 Figure: Fungi


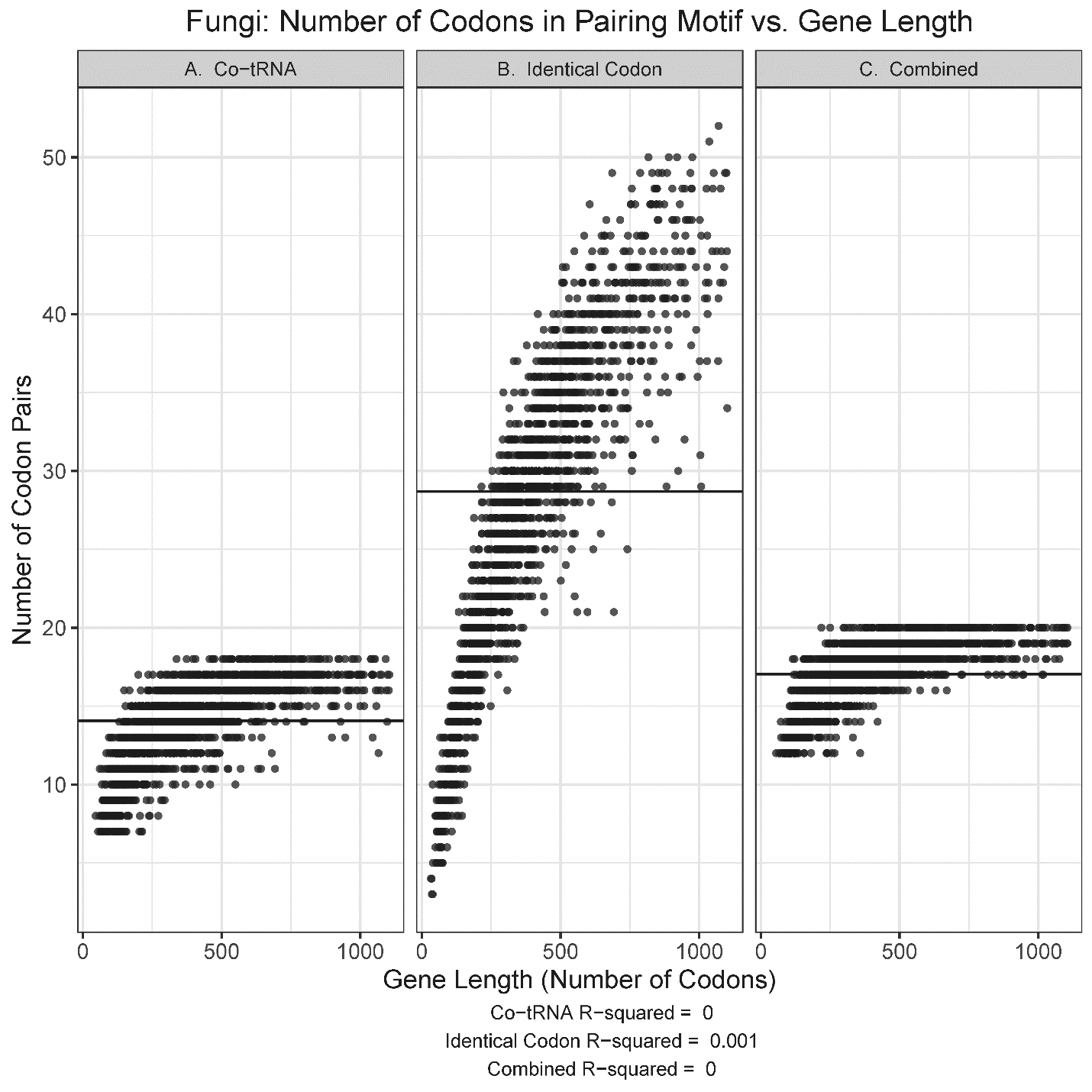


### S52 Figure: Invertebrates


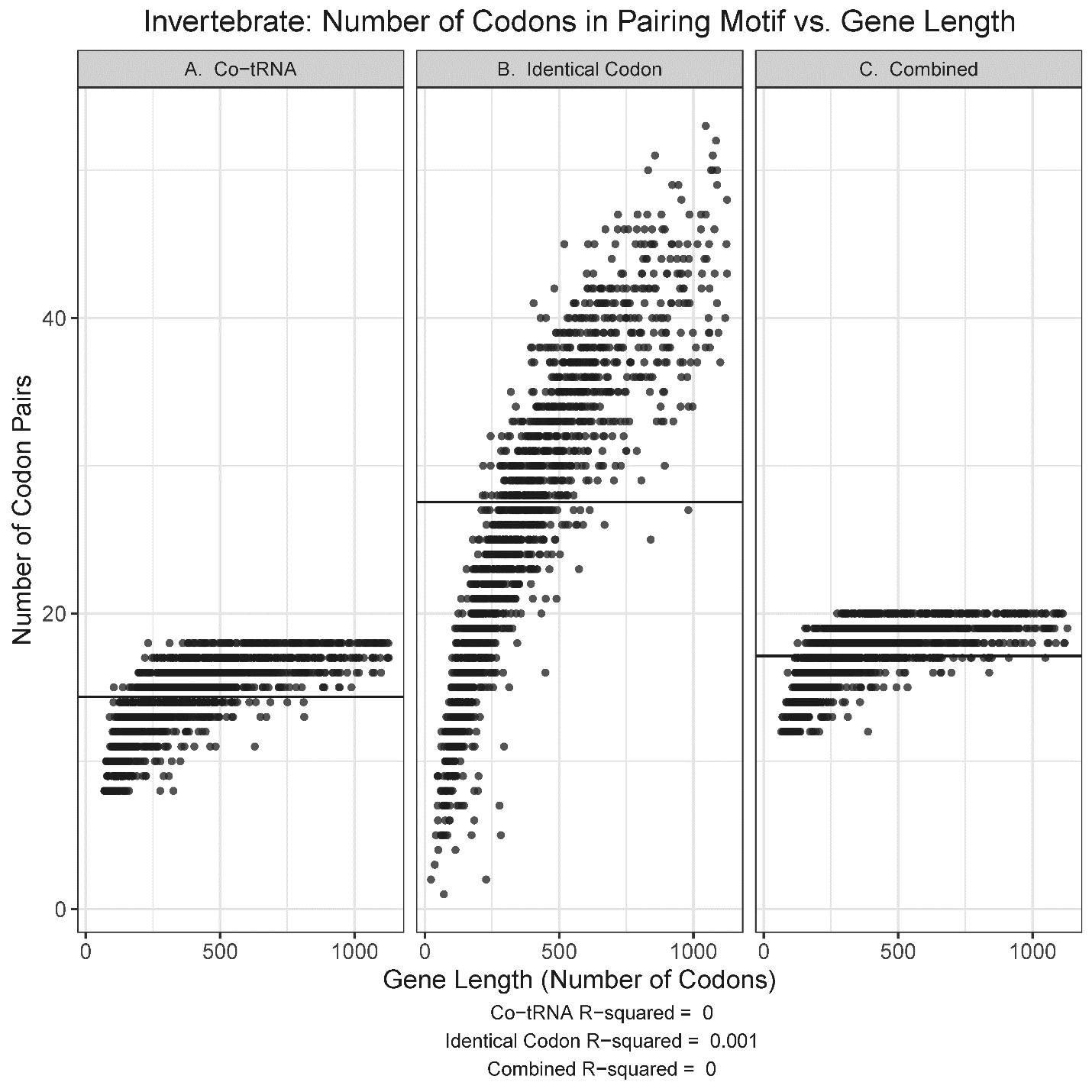


### S53 Figure: Mammals


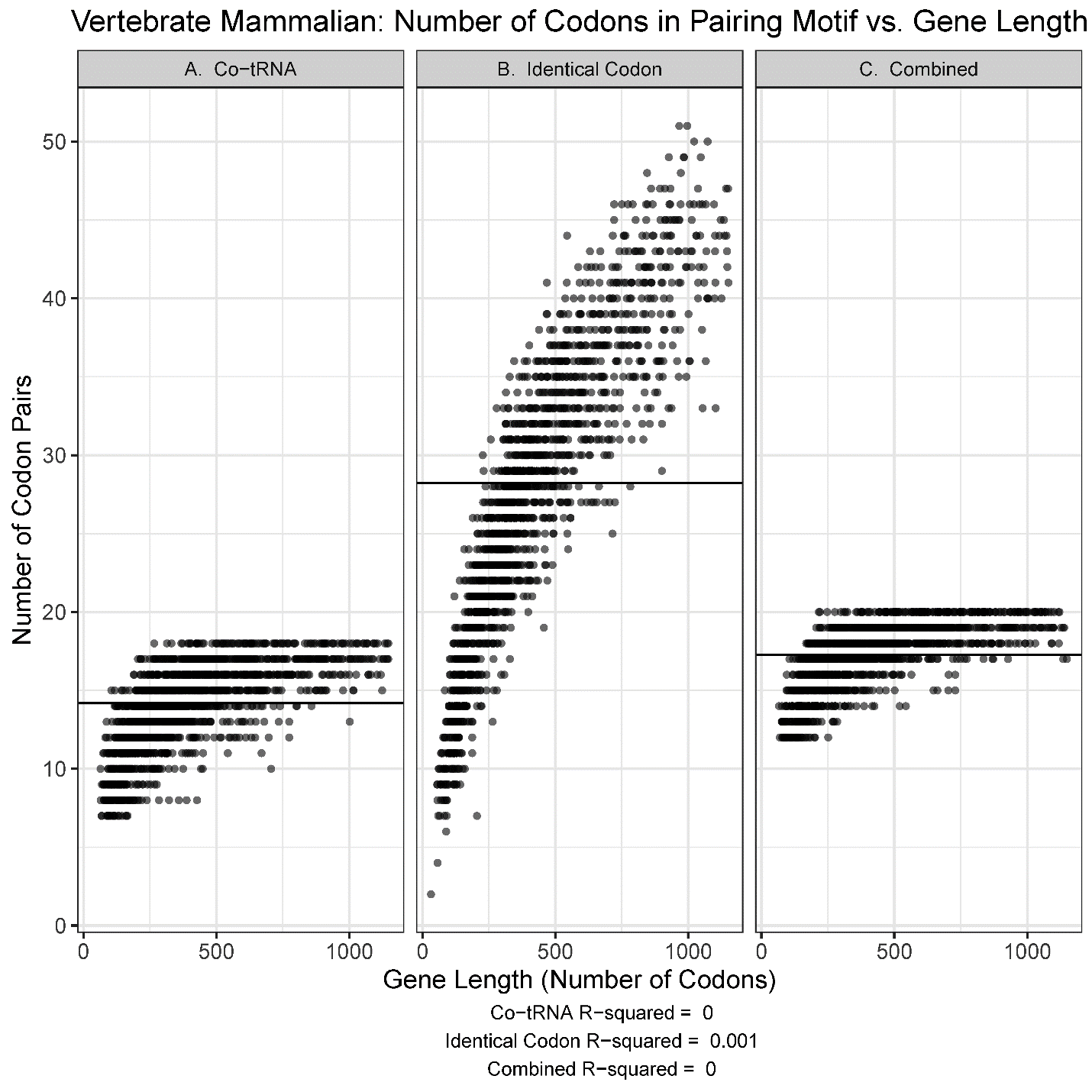


### S54 Figure: Other Vertebrates


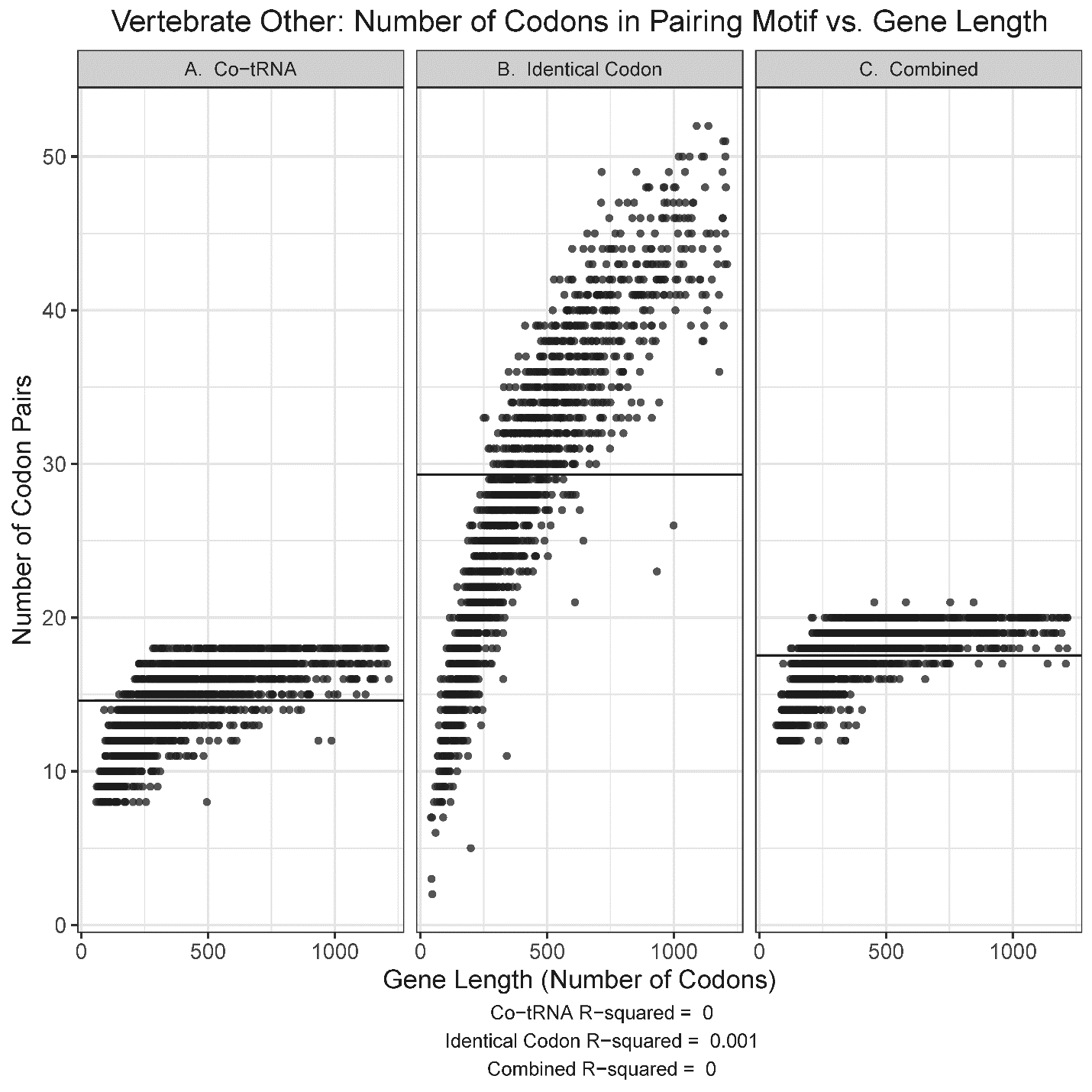


### S55 Figure: Plants


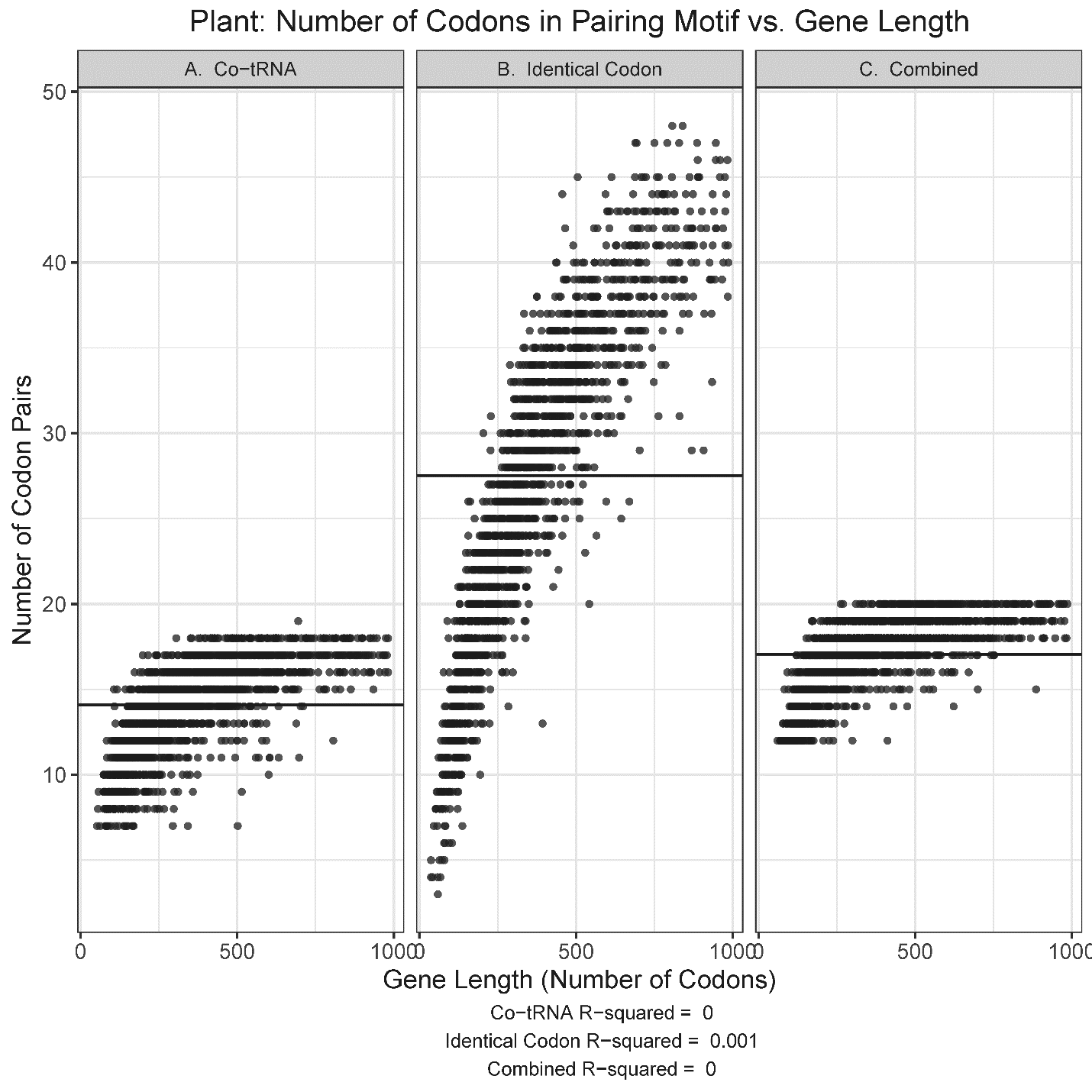


### S56 Figure: Protozoa


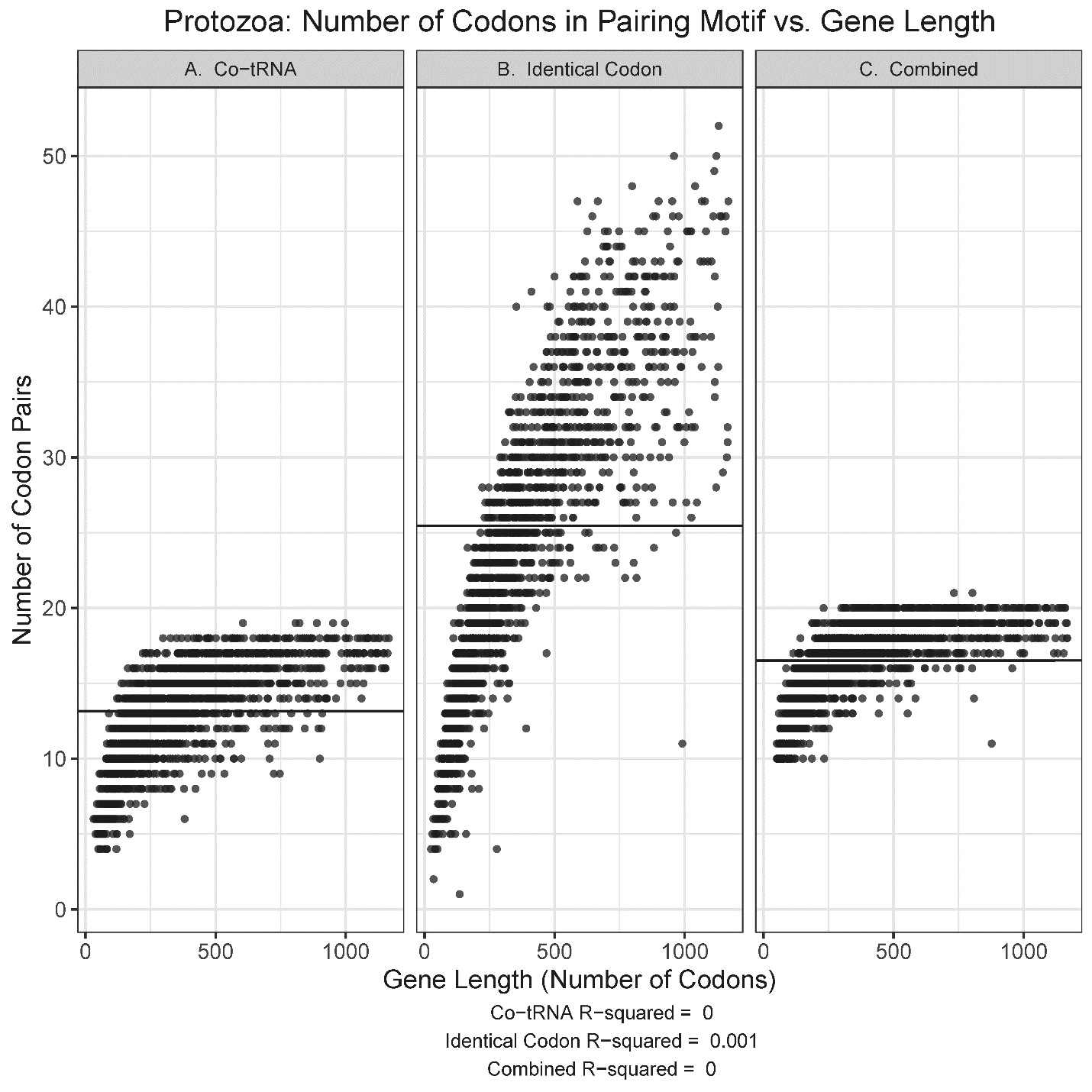


### S57 Figure: Viruses


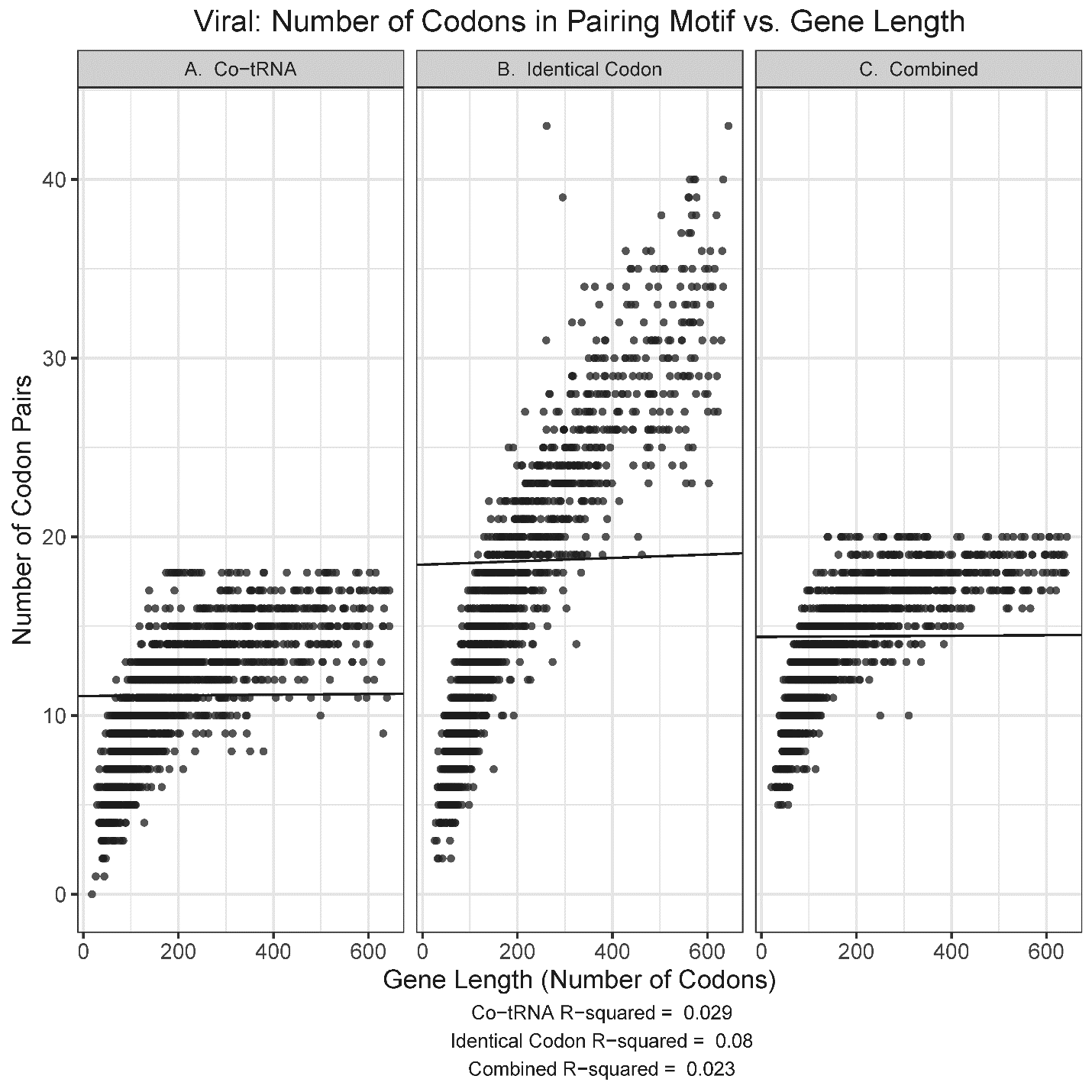


## Saturation Analysis

### S58 Figure: Archaea


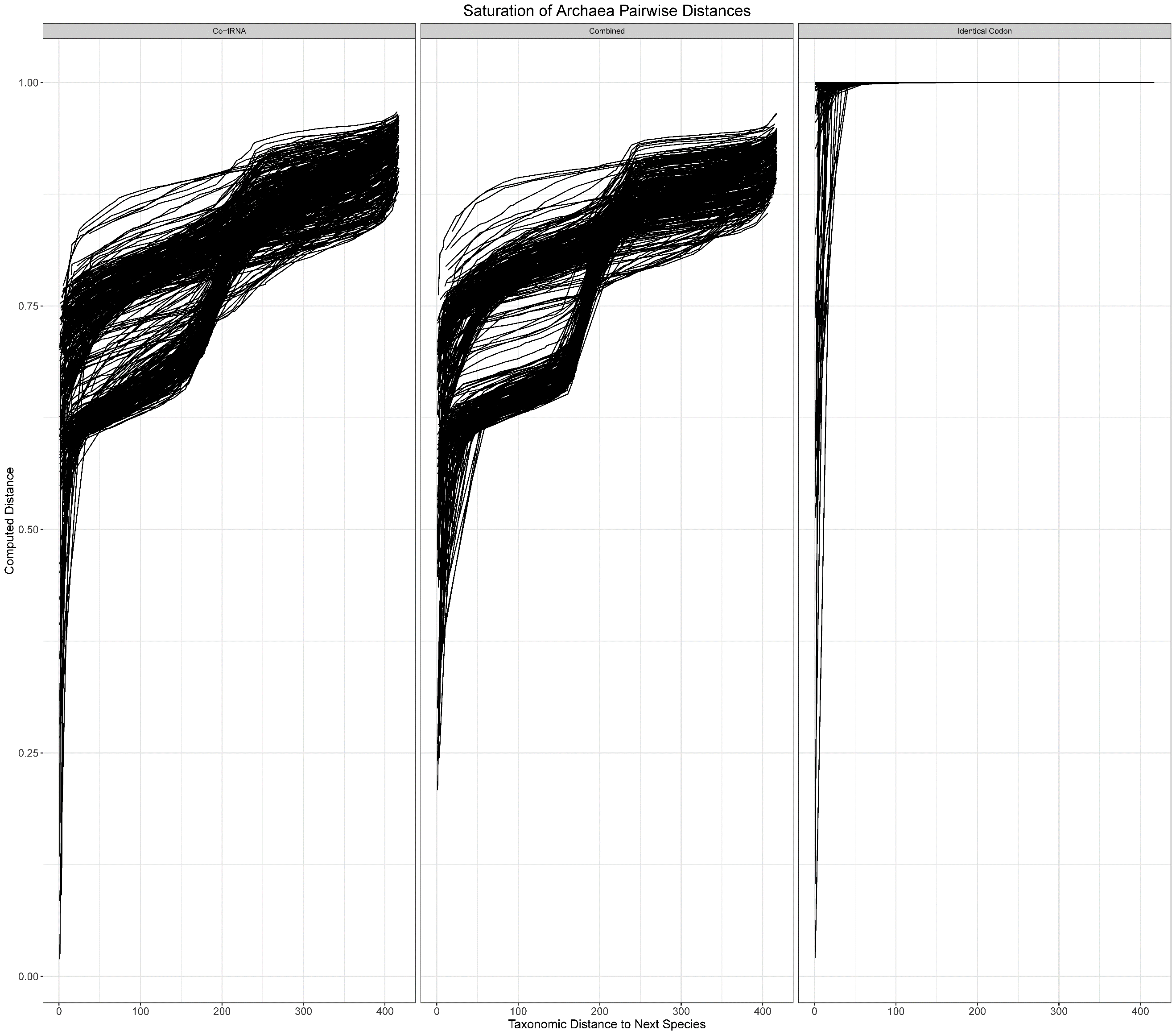


### S59 Figure: Bacteria


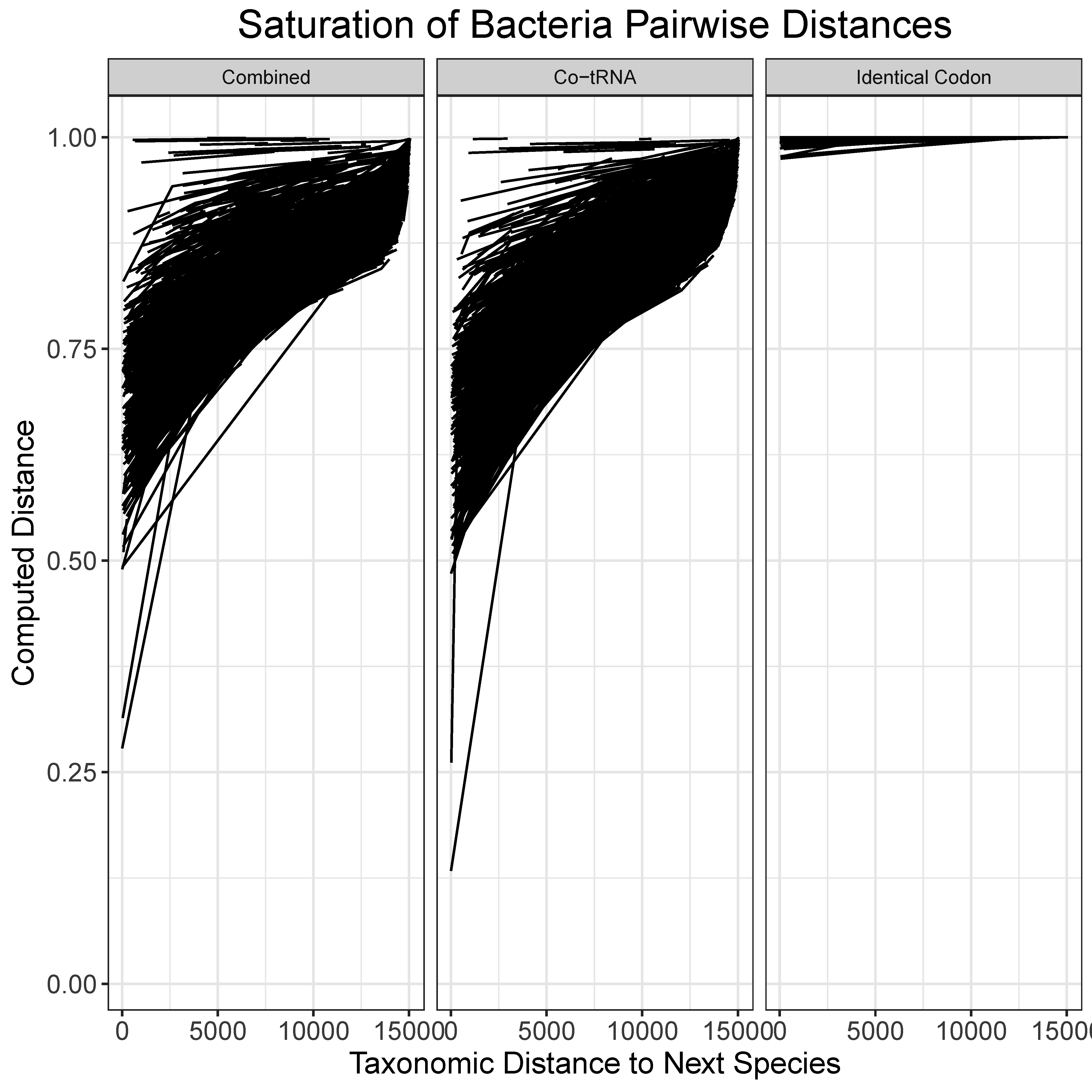


### S60 Figure: Fungi


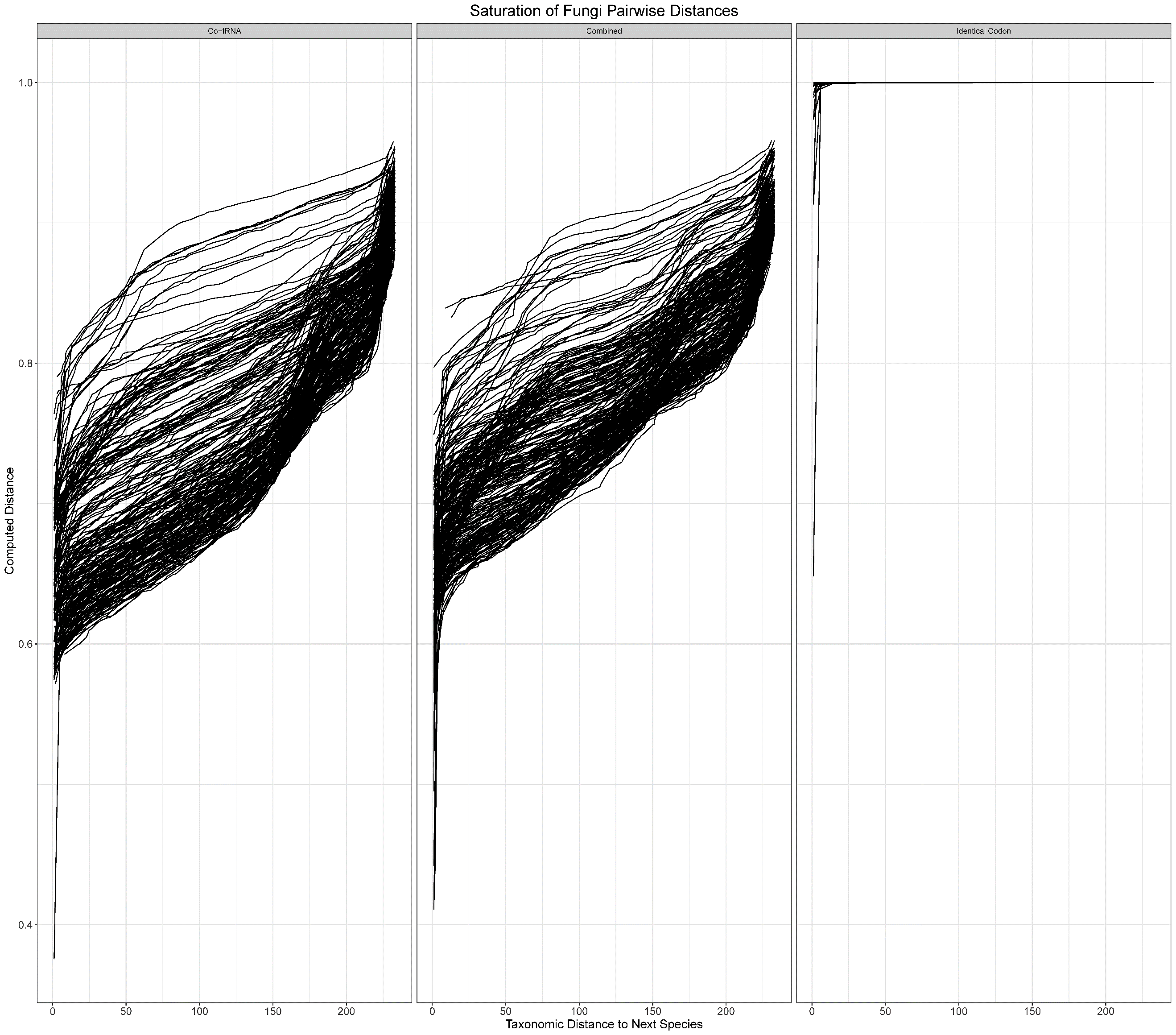


### S61 Figure: Invertebrates


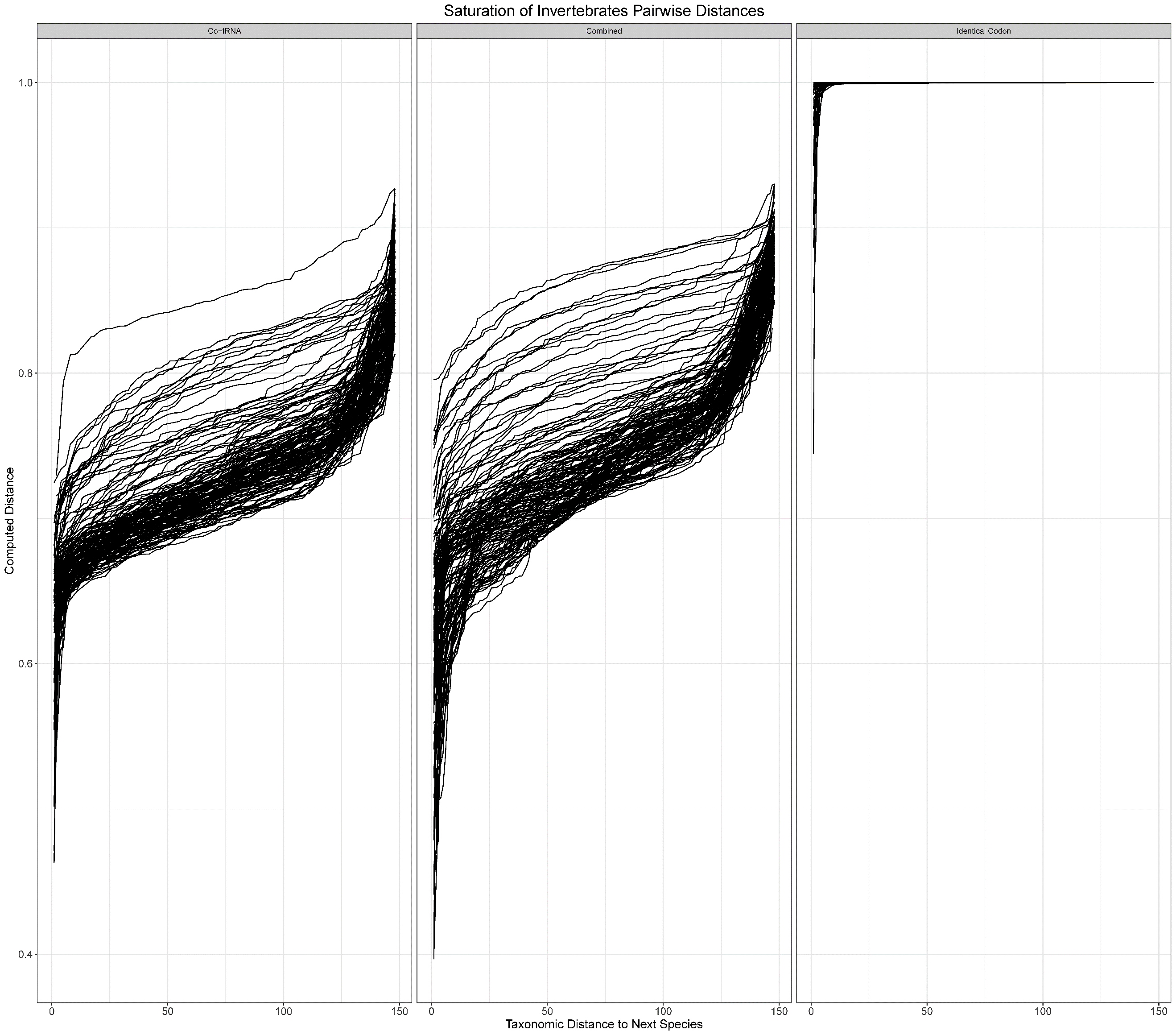


### S62 Figure: Mammals


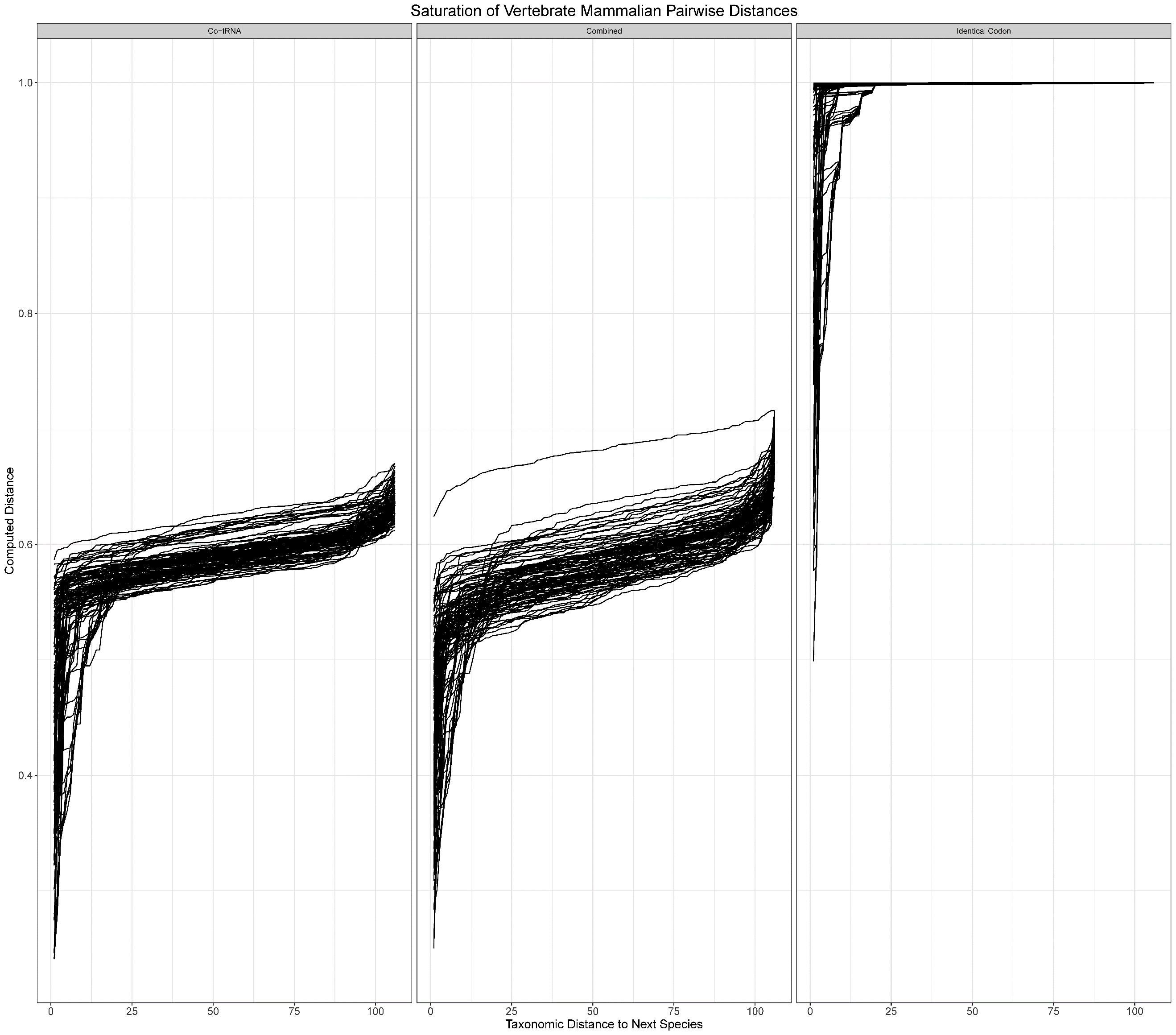


### S63 Figure: Other Vertebrates


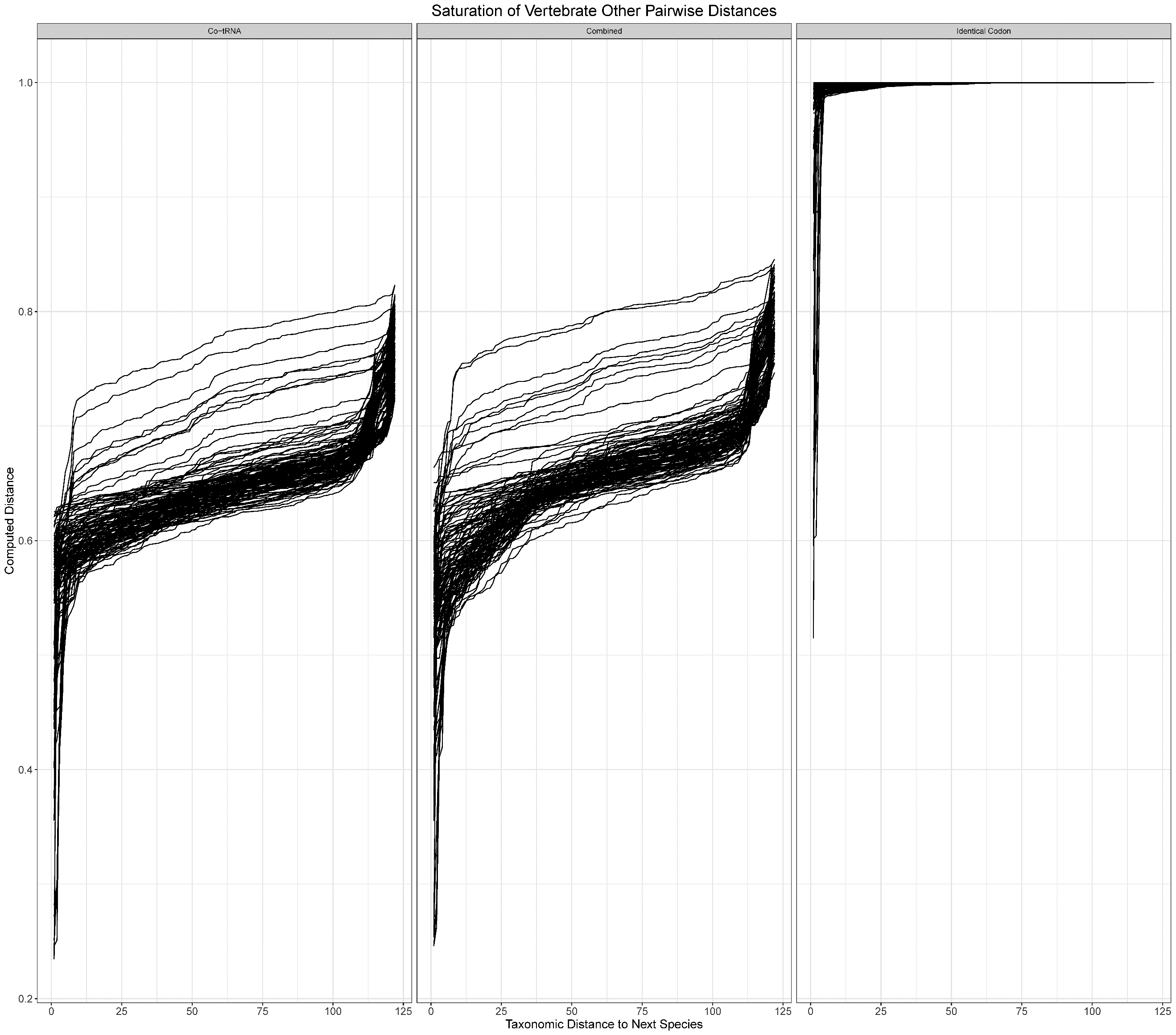


### S64 Figure: Plants


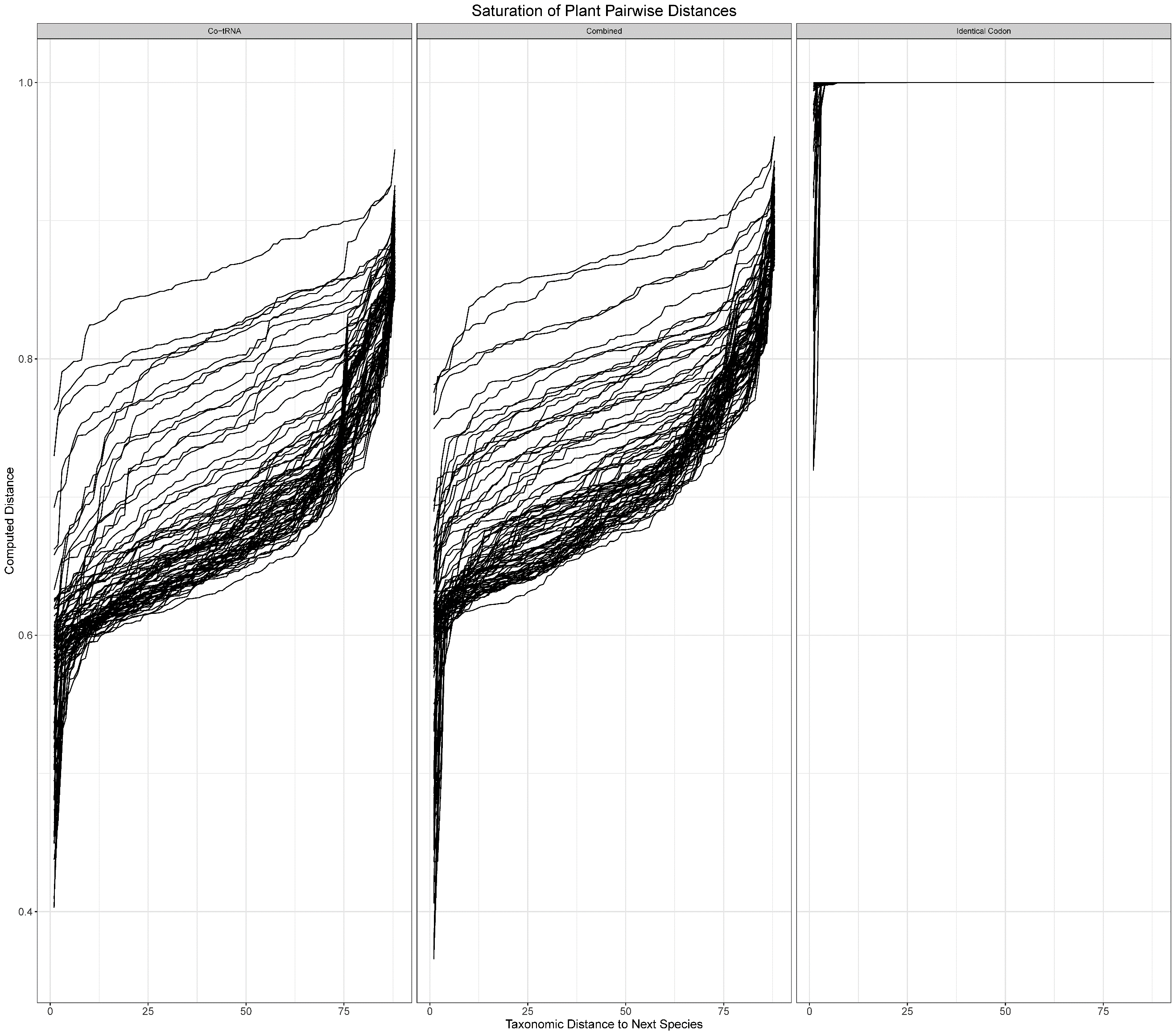


### S65 Figure: Protozoa


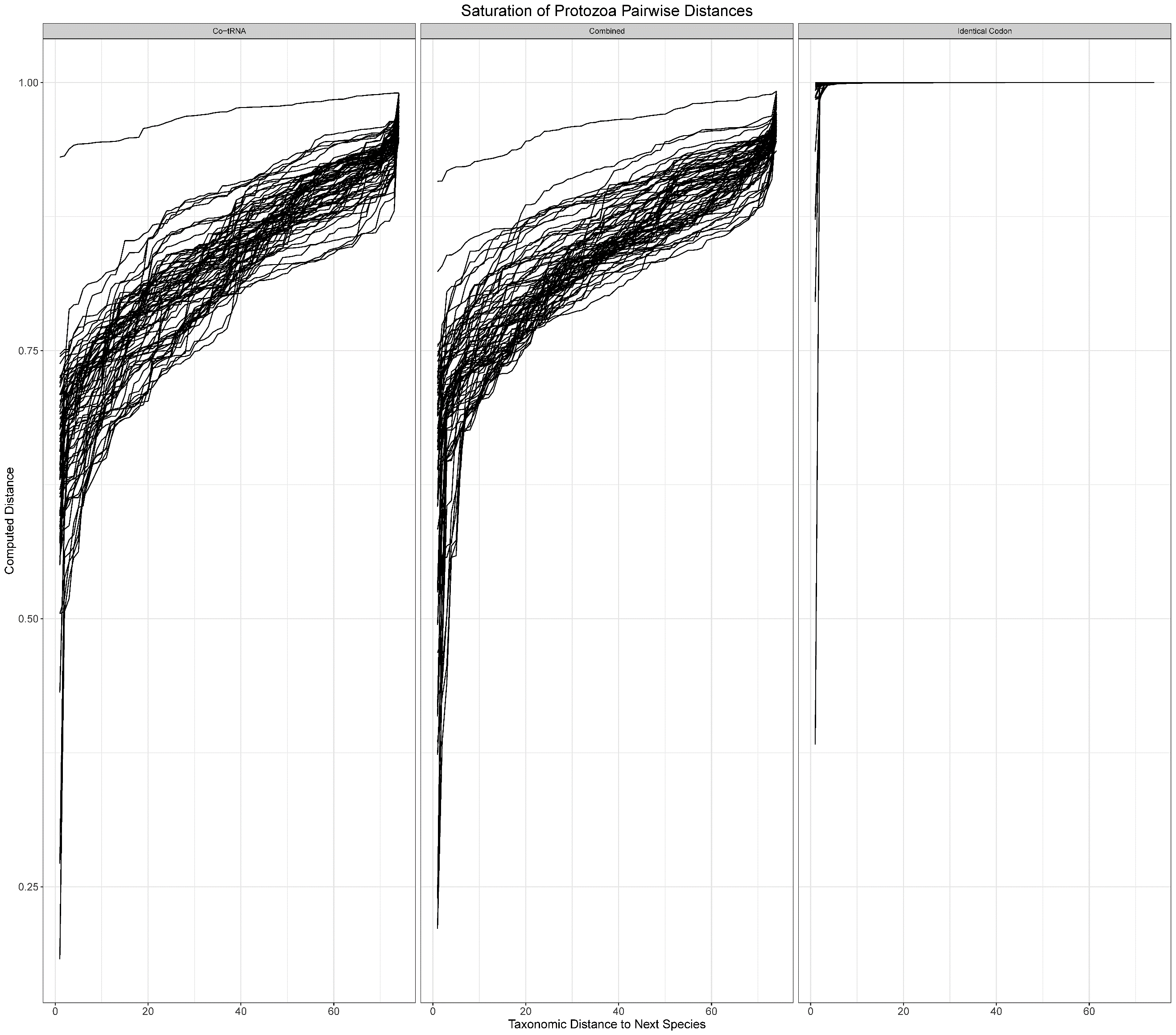


### S66 Figure: Viruses


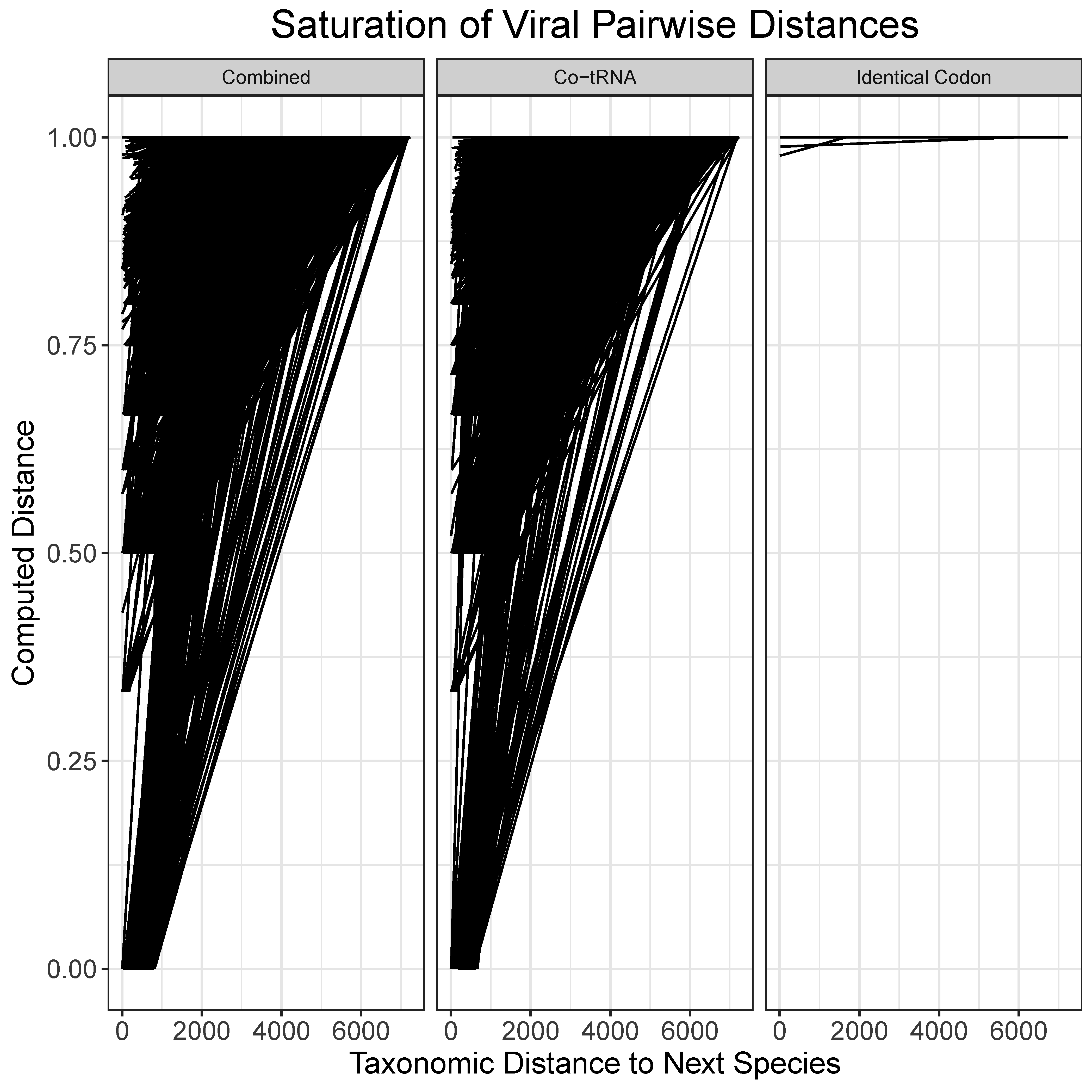


## Retention Index Analysis

### S67 Figure: Archaea


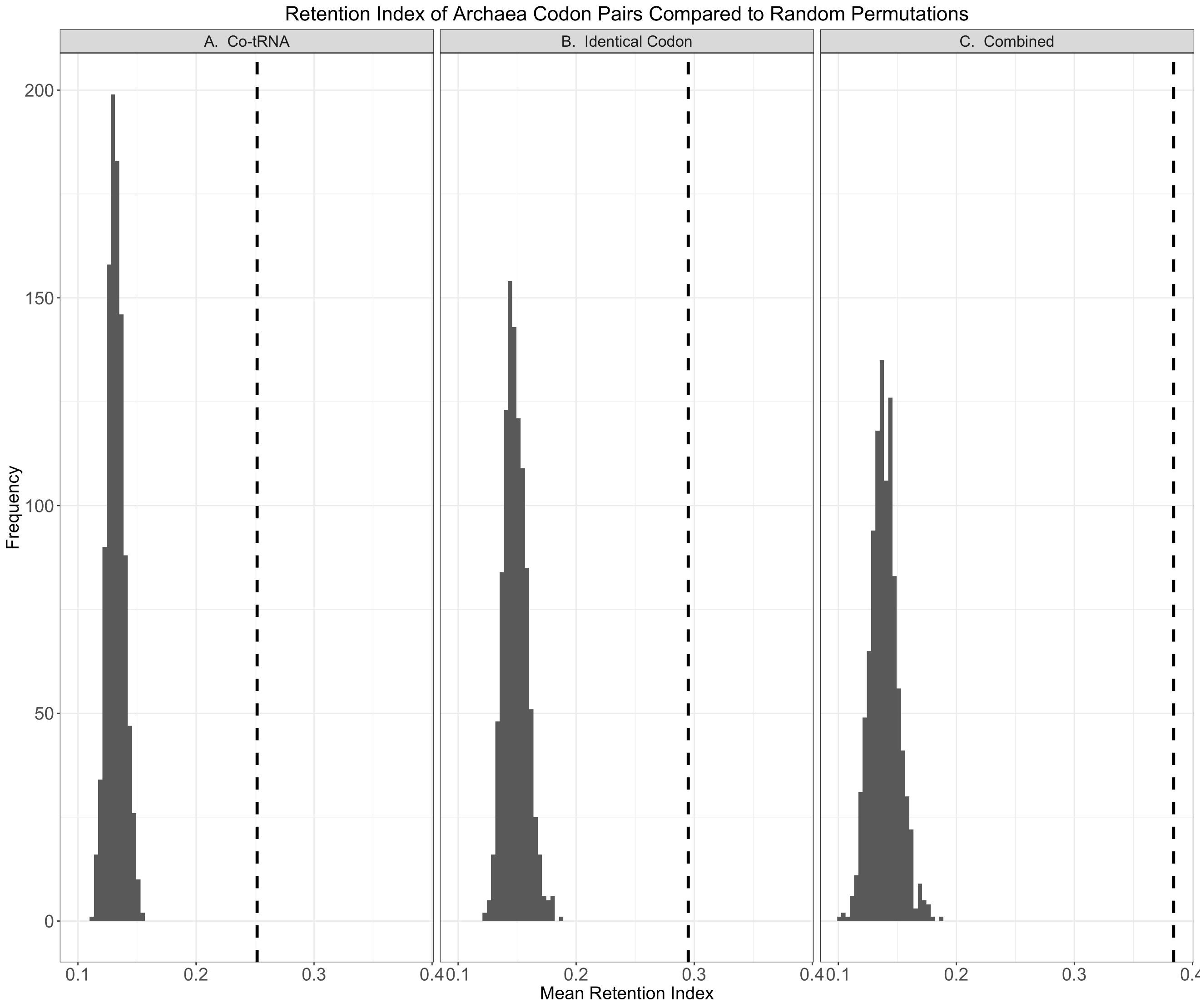


### S68 Figure: Invertebrates


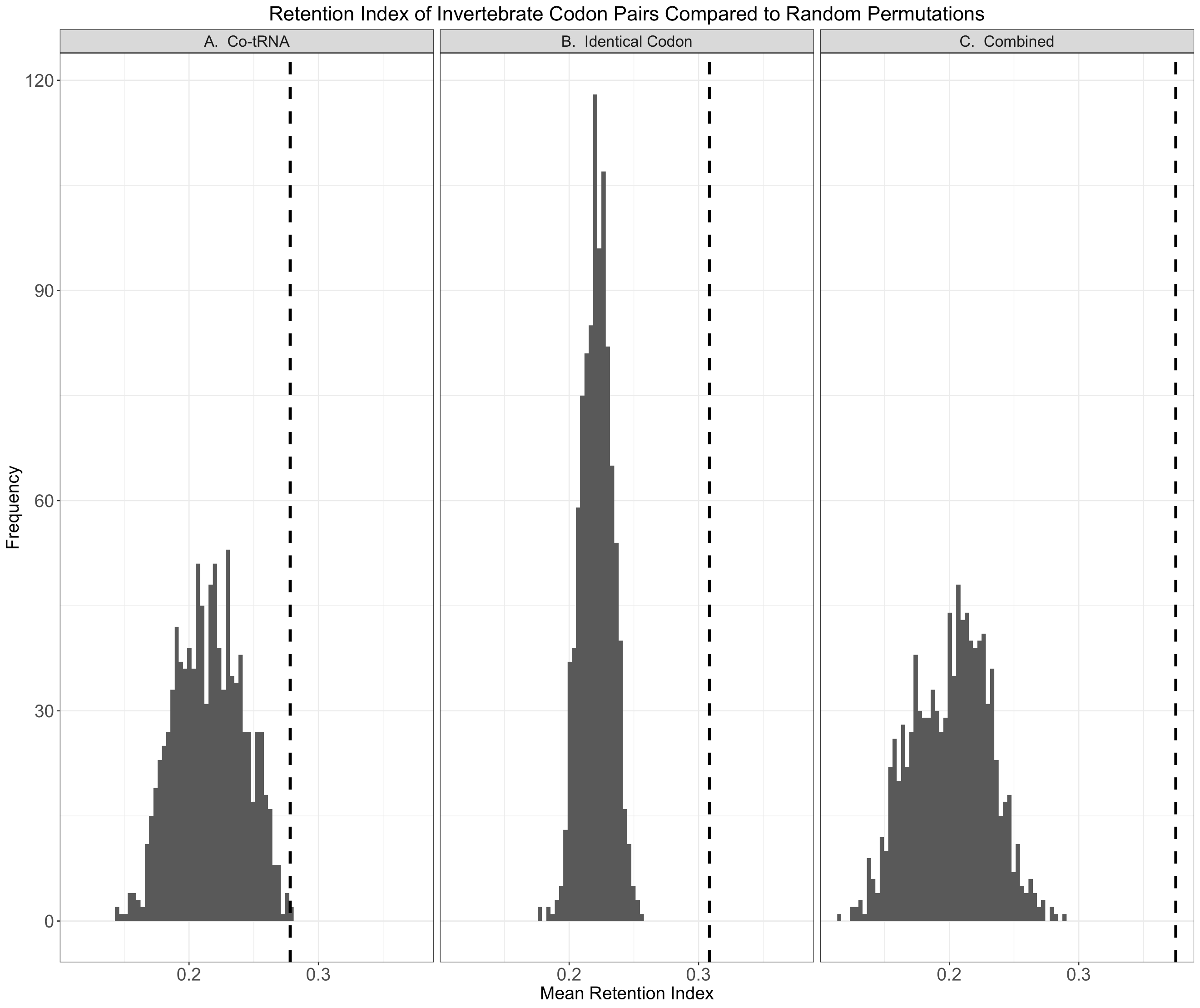


### S69 Figure: Mammals


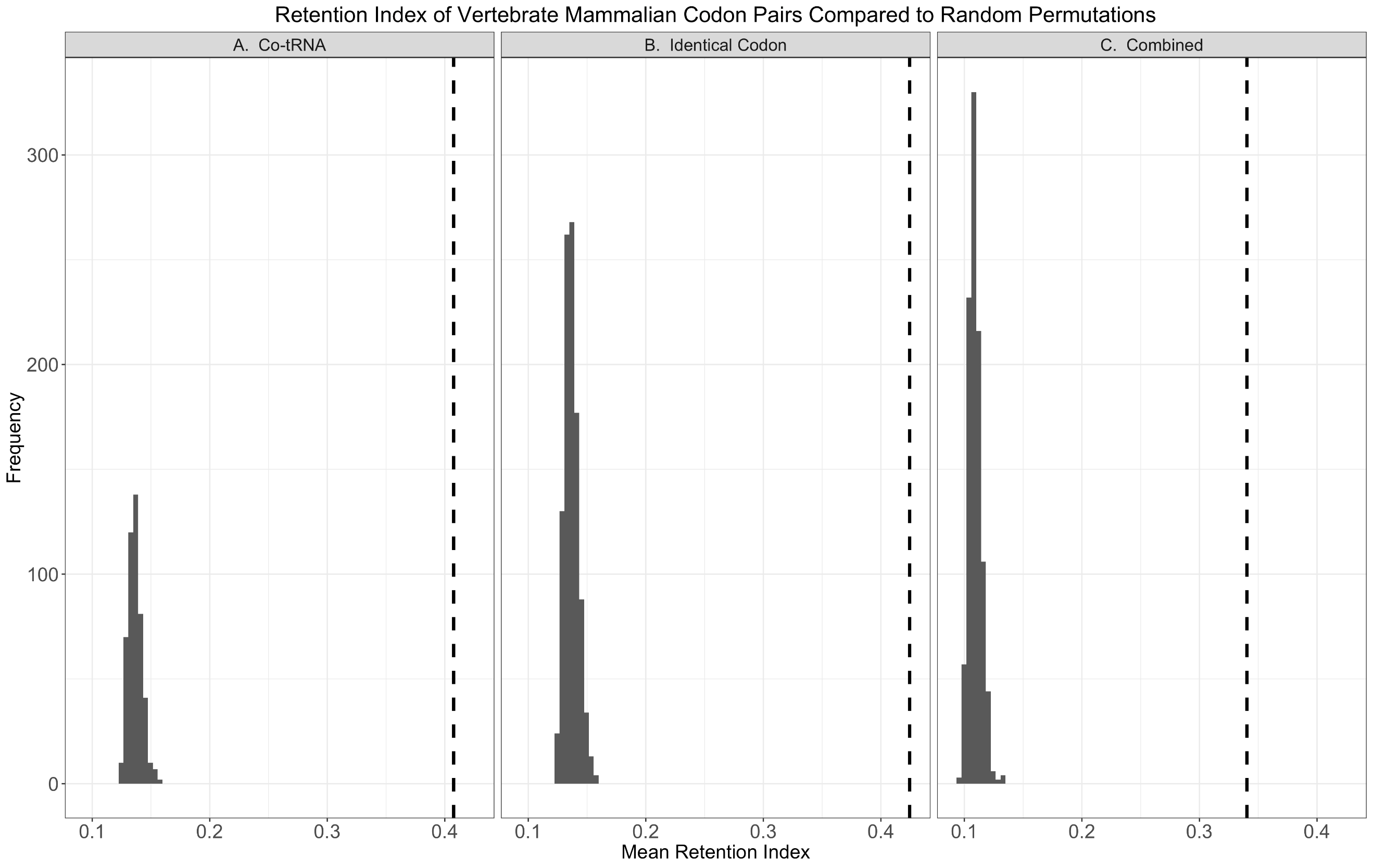


### S70 Figure: Other Vertebrates


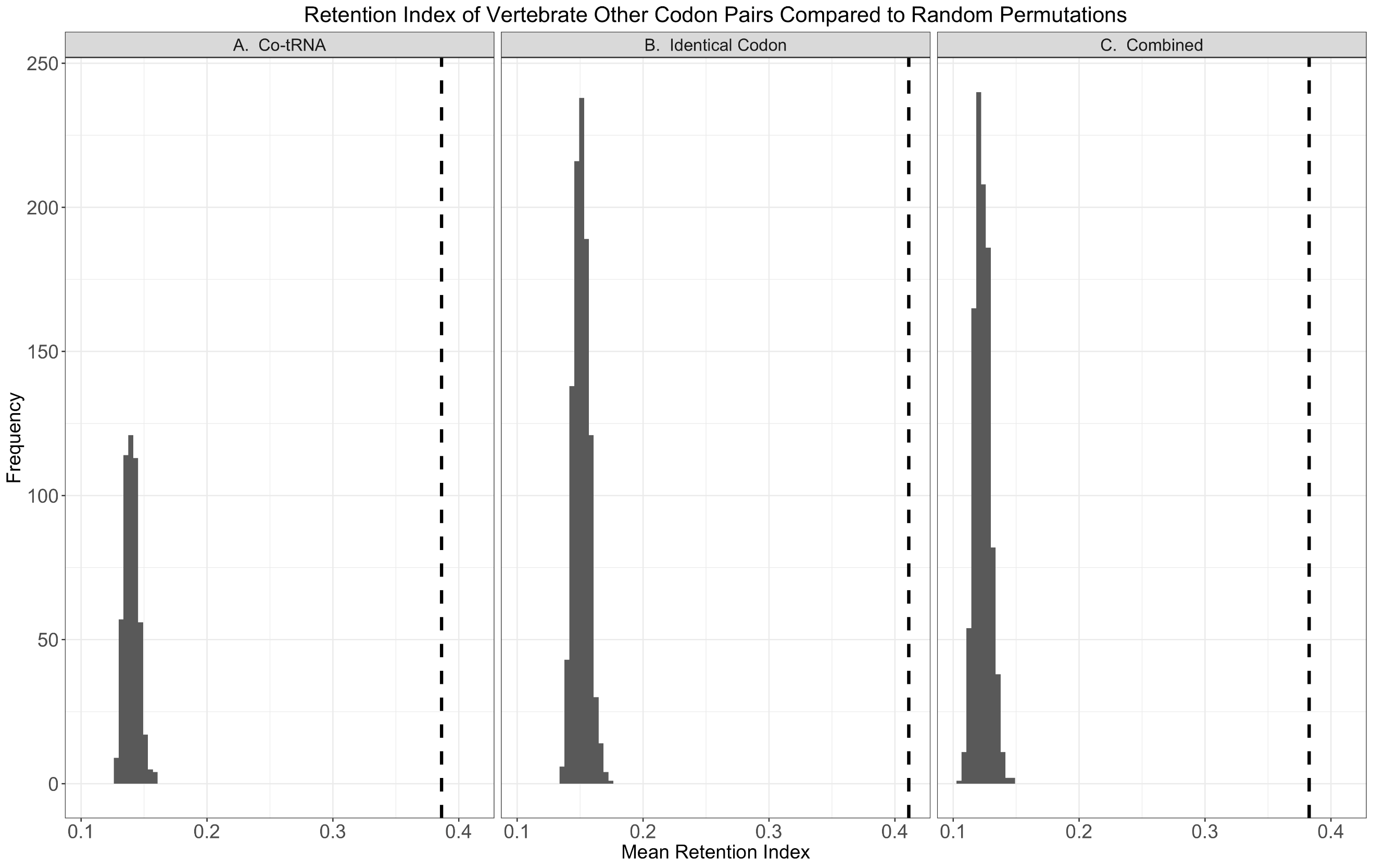


### S71 Figure: Plants


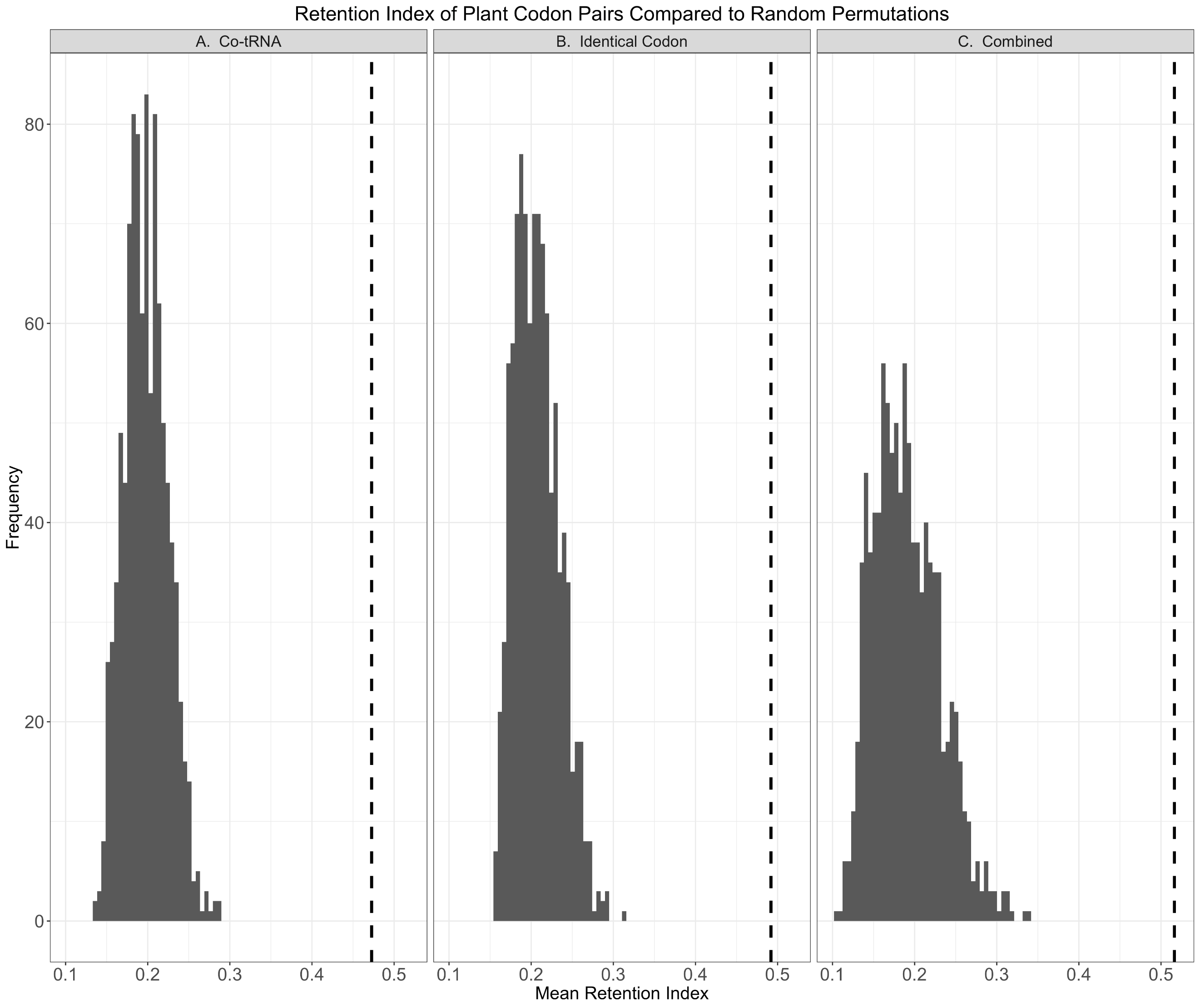


### S72 Figure: Protozoa


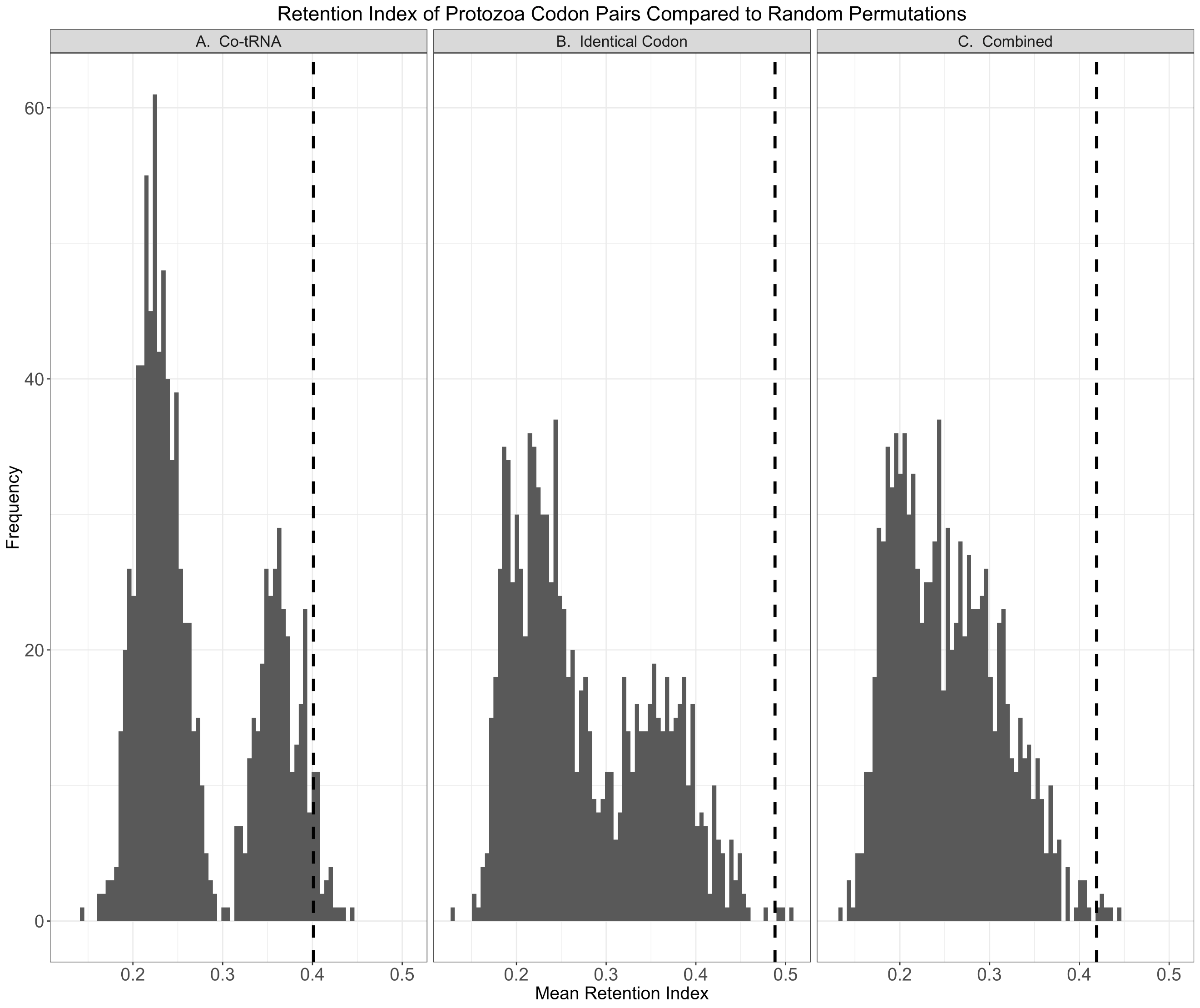


# Phylogenies

## S1 Phylogeny: Phylogeny with falcons as sister taxa to Neoaves in other vertebrates

This tree is not the current phylogeny on the OTL.


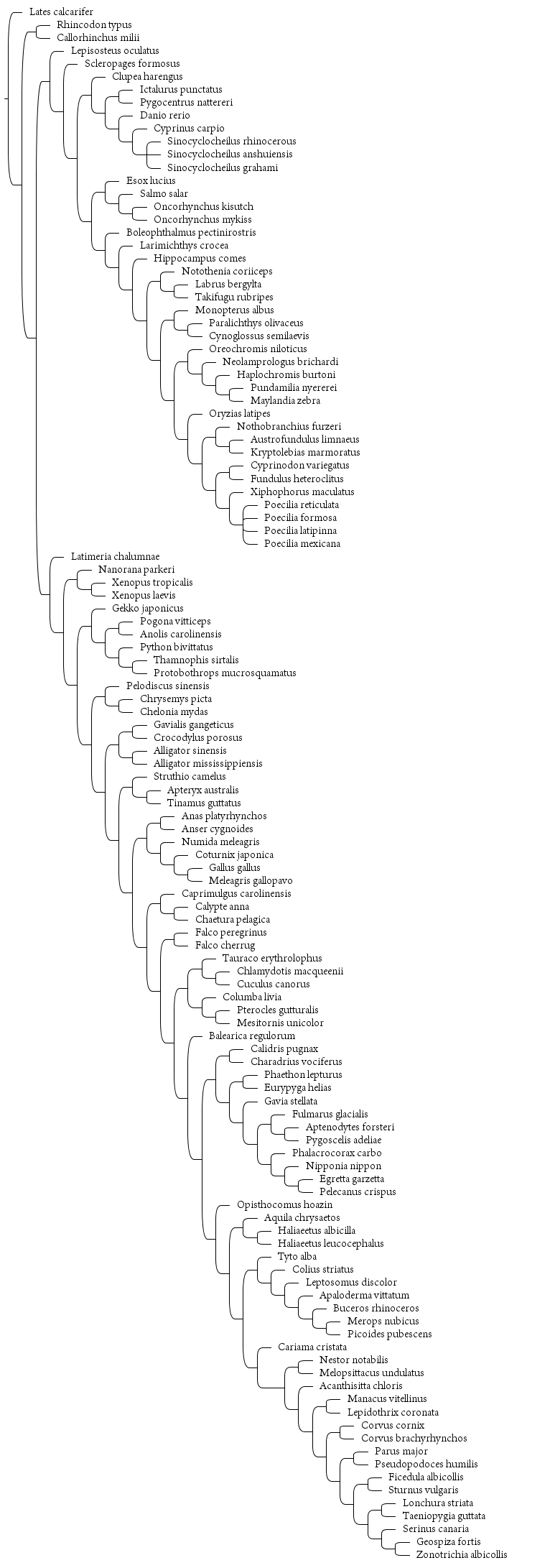


## S2 Phylogeny: Phylogeny with pigeons as sister taxa to Neoaves in other vertebrates

This tree is currently on the OTL.


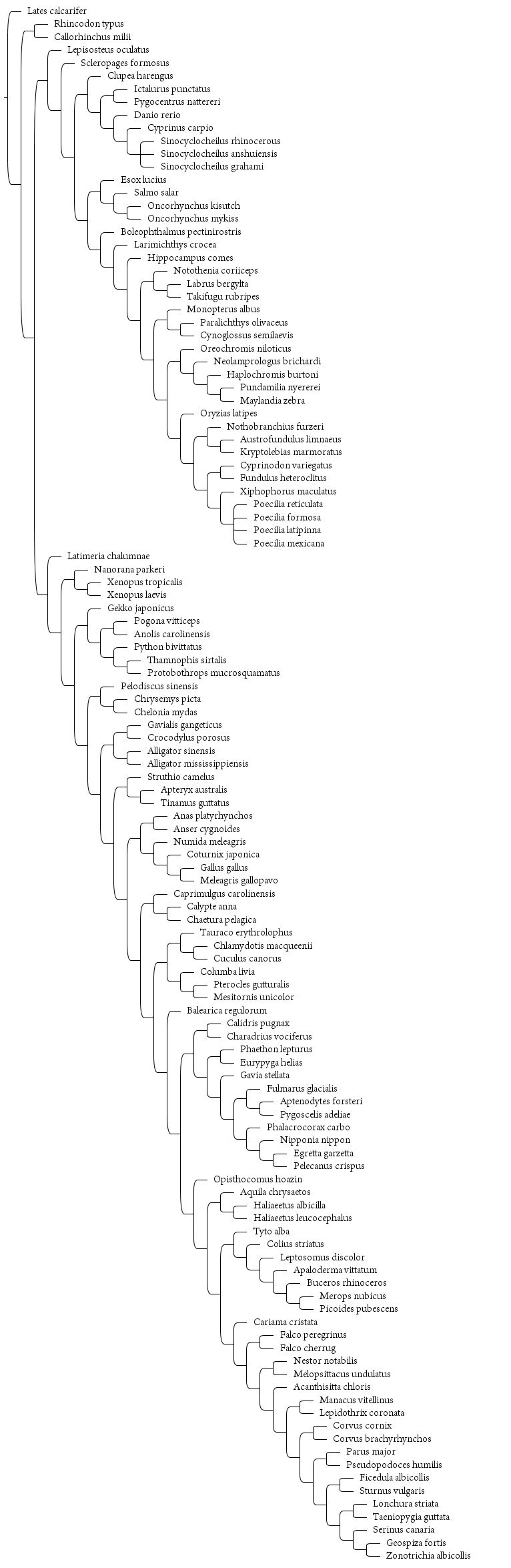


# Supplementary References

1. Miller JB, McKinnon LM, Whiting MF, Ridge PG. CAM: An alignment-free method to recover phylogenies using codon aversion motifs. PeerJ Preprints. 2019;7:e27756v1. doi: 10.7287/peerj.preprints.27756v1.

2. Sievers F, Higgins DG. Clustal Omega for making accurate alignments of many protein sequences. Protein Sci. 2018;27(1):135-45. Epub 2017/09/09. doi: 10.1002/pro.3290. PubMed PMID: 28884485; PubMed Central PMCID: PMCPMC5734385.

3. Nguyen LT, Schmidt HA, von Haeseler A, Minh BQ. IQ-TREE: a fast and effective stochastic algorithm for estimating maximum-likelihood phylogenies. Mol Biol Evol. 2015;32(1):268-74. Epub 2014/11/06. doi: 10.1093/molbev/msu300. PubMed PMID: 25371430; PubMed Central PMCID: PMCPMC4271533.

4. Jun S-R, Sims GE, Wu GA, Kim S-H. Whole-proteome phylogeny of prokaryotes by feature frequency profiles: An alignment-free method with optimal feature resolution. Proceedings of the National Academy of Sciences. 2010;107(1):133-8. doi: 10.1073/pnas.0913033107.

5. Sims GE, Jun SR, Wu GA, Kim SH. Alignment-free genome comparison with feature frequency profiles (FFP) and optimal resolutions. Proc Natl Acad Sci U S A. 2009;106(8):2677-82. Epub 2009/02/04. doi: 10.1073/pnas.0813249106. PubMed PMID: 19188606; PubMed Central PMCID: PMCPMC2634796.
